# The impact of opioid prescribing guidelines on opioid dispensation: A systematic review and meta-analysis

**DOI:** 10.1080/24740527.2026.2650299

**Published:** 2026-05-22

**Authors:** Jihoon Lim, Parker Tope, Maximilian Schaefer, Isaiah Williams, Imen Farhat, Andrea Benedetti, Dimitra Panagiotoglou

**Affiliations:** a Department of Epidemiology, Biostatistics and Occupational Health, McGill University, Montréal, QC, Canada; b Department of Epidemiology, Harvard T.H. Chan School of Public Health, Boston, MA, USA; c Research Institute, McGill University Health Centre, Montréal, QC, Canada

**Keywords:** Opioids, prescriptions, practice guidelines, interrupted time series analysis, meta-analysis

## Abstract

**Background:**

Opioid prescribing guidelines for chronic noncancer pain (CNCP) aim to reduce opioid volume and dosage while addressing risks such as misuse, poisoning, and death. However, these guidelines may have spillover effects on patient populations beyond the intended scope.

**Aims:**

This study aims to summarize the impact of prescribing guidelines on opioid use prevalence, incidence, and dosage across multiple jurisdictions and patient populations.

**Methods:**

In October 2025, we systematically searched MEDLINE, Scopus, Web of Science, and PsycInfo for studies on the effects of opioid prescribing guidelines in adult populations. Primary outcomes included opioid use prevalence, incidence, dosage, and duration. We calculated study-level effect estimates using interrupted time series analyses and conducted meta-analyses with random effects models to estimate the pooled effect of the guidelines on the aforesaid outcomes.

**Results:**

Of 190 records, 71 studies met inclusion criteria for meta-analysis. After guideline implementation, morphine milligram equivalent (MME) dosage per person and prescription duration decreased annually by 10.4% (95% confidence interval [CI] −19.2% to −0.6%) and 5.6% (95% CI −8.1% to −3.0%), respectively, among patients with CNCP. In the general population, MME dosage per person decreased annually by 7.6% (95% CI −11.3% to −3.7%), whereas duration remained stable over time (−1.4%; 95% CI −6.5% to 4.0%). Following guideline implementation, declines in prevalence, dosage, and duration were also observed in patients living with cancer or treated for acute pain.

**Conclusion:**

Guidelines reduced opioid prescribing for CNCP. Future research is needed to examine whether and to what extent similar changes occurred among other patient populations.

## RÉSUMÉ

**Contexte :** Les lignes directrices sur la prescription d’opioïdes pour la douleur chronique non cancéreuse (DCNC) visent à réduire le volume et le dosage des opioïdes, tout en abordant les risques tels que l’usage abusif, l’intoxication et le décès. Cependant, ces directives peuvent avoir des effets secondaires sur des populations de patients au-delà de la portée prévue.

**Objectifs :** Cette étude vise à résumer l’impact des lignes directrices de prescription sur la prévalence, l’incidence et le dosage de l’utilisation d’opioïdes à travers plusieurs juridictions et populations de patients.

**Méthodes :** En octobre 2025, nous avons effectué une recherche systématique dans MEDLINE, Scopus, Web of Science et PsycInfo pour des études portant sur les effets des lignes directrices de prescription d’opioïdes chez les populations adultes. Les principaux critères d’évaluation comprenaient la prévalence, l’incidence, le dosage et la durée de l’utilisation d’opioïdes. Nous avons calculé les estimations d’effet au niveau de l’étude à l’aide d’analyses de séries temporelles interrompues et avons réalisé des méta-analyses avec des modèles à effets aléatoires pour estimer l’effet regroupé des lignes directrices sur les résultats susmentionnés.

**Résultats :** Sur 190 enregistrements, 71 études répondaient aux critères d’inclusion pour la méta-analyse. Après la mise en œuvre des lignes directrices, le dosage en équivalent milligramme de morphine (EMM) par personne et la durée de la prescription ont diminué annuellement de 10,4 % (intervalle de confiance [IC] à 95 % : -19,2 % à -0,6 %) et de 5,6 % (IC à 95 % : -8,1 % à -3,0 %), respectivement, chez les patients atteints de DCNC. Dans la population générale, le dosage EMM par personne a diminué annuellement de 7,6 % (IC à 95 % : -11,3 % à -3,7 %), tandis que la durée est restée stable dans le temps (-1,4 %, IC à 95 % : -6,5 % à 4,0 %). À la suite de la mise en œuvre des lignes directrices, des baisses de la prévalence, du dosage et de la durée ont également été observées chez les patients vivant avec un cancer ou traités pour une douleur aiguë.

**Conclusions :** Les lignes directrices ont réduit la prescription d’opioïdes pour la DCNC. Des recherches futures sont nécessaires pour examiner si et dans quelle mesure des changements similaires se sont produits parmi d’autres populations de patients.

## Introduction

The early 2000s witnessed a global surge in opioid consumption,^1^ accompanied by rising rates of opioid use disorder, overdose, and mortality,^2–4^ culminating in epidemics in some countries.^5,6^ These crises prompted strategies to curb opioid use, including interventions targeting opioid supply, such as drug delistings, prescription monitoring programs, and prescribing guidelines for chronic noncancer pain (CNCP). Opioid prescribing for CNCP, which often leads to long-term opioid use, was particularly targeted due to evidence questioning the safety and effectiveness of long-term opioid therapy.^7^ Although opioids may relieve pain, the associated risks, including opioid use disorder, overdose, and death, frequently outweigh their benefits.^7,8^

Beginning in the mid-2000s, health institutions across the world released opioid prescribing guidelines to standardize opioid use for CNCP.^9–20^ Though intended to promote safer prescribing, these guidelines may have contributed to restricted access to adequate pain management, particularly for populations outside their scope, such as those with acute, cancer, or palliative pain. Misinterpretation by physicians also led to harmful practices like abrupt tapering or discontinuation for patients on long-term opioids, pushing some toward unregulated markets.^8^ Thus, though the guidelines reduced overprescribing for CNCP, they may have inadvertently encouraged underprescribing in other patient groups.

Existing studies evaluating the impact of opioid prescribing guidelines have reported reductions in opioid dispensations and high-dose opioid prescriptions.^21–24^ However, most studies focus on a single guideline or jurisdiction, limiting insight into how guideline effects may evolve over time or vary across geographic regions. Studies also differ in the outcomes assessed and in how effects are reported (e.g., regression coefficients versus percentage changes), hindering comparison across guidelines and settings. To date, no comprehensive synthesis has evaluated the impact of opioid prescribing guidelines across multiple prescribing metrics using a common analytical framework. Moreover, evidence on spillover effects, particularly among guideline-exempt populations including patients with acute, cancer, or palliative pain, remains limited and underexplored. Consequently, the overall population-level impact of opioid prescribing guidelines and the extent to which their effects differ across pain populations remains unclear. Therefore, this systematic review and meta-analysis aimed to assess the impact of opioid prescribing guidelines on opioid dispensation and dosage on patients living with CNCP as well as with guideline-exempt pain types.

## Methods

We report the results of our systematic review and meta-analysis in accordance with the Preferred Reporting Items for Systematic Reviews and Meta-Analyses.^25^

### Data sources and search strategy

#### Identification of studies

**Figure 1.**
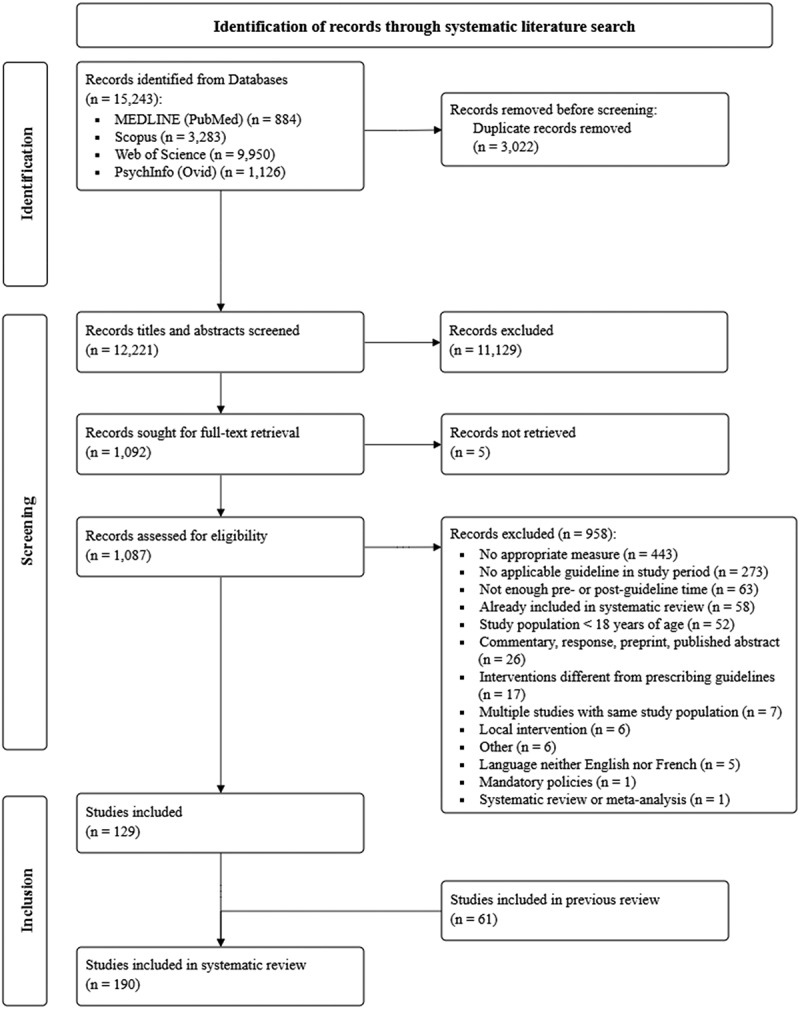
Preferred Reporting Items for Systematic Reviews and Meta-Analyses flowchart of study selection process.

This review built upon and expanded a previous review commissioned by Health Canada that mainly focused on three guidelines in Canada and the United States by the College of Physicians and Surgeons of British Columbia,^11^ the U.S. Centers for Disease Control and Prevention,^12^ and Health Canada.^9^ For internal validation purposes, we manually reviewed the reference lists of studies included in the previous review to identify records missed in our initial systematic search. We then expanded the project to include other international guidelines, prompting a repeat of the previous search. On May 21, 2024, we systematically searched MEDLINE (PubMed), Scopus, Web of Science, and PsycInfo (Ovid) for peer-reviewed articles and gray literature (e.g., government reports) on opioid prescribing trends to date. Our search strategy included keyword roots (e.g., “opioid analgesics,” “practice guidelines,” “practice patterns,” “standards,” “prescribing,” and “chronic pain”), their wildcard expansions, and associated MeSH (Medical Subject Headings) terms (Table S1). The search was updated on January 3, 2025, and October 14, 2025 (Figure 1; more details in Figure S1).

#### Identification of guidelines

We conducted an environmental scan for opioid prescribing guidelines for chronic noncancer pain by searching Google and Google Scholar, as well as reviewing the references of eligible studies. Germane opioid prescribing guidelines provided definitions of and were specific to prescribing practices for chronic noncancer pain (i.e., did not include additional recommendations on prescribing for other pain types) and explicitly provided recommendations related to dosage and/or duration of initial or continued opioid prescribing.

When multiple versions or updates of a guideline existed, earlier versions were not excluded, because our objective was to assess the impact of the guideline in effect during the study period of each included analysis. For studies with observation periods spanning multiple guideline updates, each guideline implementation was treated as a distinct intervention, and postintervention effects were evaluated separately for nonoverlapping periods. Guidelines that addressed opioid prescribing for indications beyond CNCP without providing CNCP-specific recommendations were excluded. Details regarding guideline exclusion decisions are provided in Table S2.

### Eligibility criteria and definitions

#### Population and pain type

We categorized records by pain type: (1) general/nonspecific, (2) CNCP, (3) chronic, (4) acute, (5) cancer related, and (6) palliative. General/nonspecific studies reported opioid prescribing outcomes without specifying pain type. CNCP studies focused on pain lasting 3 or more months unrelated to recent cancer diagnoses, consistent with guideline definitions. Chronic pain studies captured dispensations to patients for >3 months but did not exclude patients living with cancer. Acute pain studies assessed prescribing for short-term pain (<1 month) due to sudden health events (e.g., trauma, surgery) or in emergency department settings. Cancer-related pain studies examined prescribing for patients living with cancer or oncologists’ prescribing practices, whereas palliative pain studies focused on terminal pain excluding end-stage cancer cases.

#### Intervention

The intervention of interest was the implementation of opioid prescribing guidelines or clinical practice standards at the regional, national, or jurisdictional level. We classified studies as guideline specific if they explicitly estimated the impact of a guideline or non-guideline specific if they reported prescribing trends during periods of guideline implementation.

#### Outcomes

We grouped study outcomes into five types: (1) prevalence, (2) incidence, (3) dosage, (4) duration, and (5) tapering/discontinuation, reflecting guideline goals to reduce the frequency, dosage, and duration of opioid use. Prevalence outcomes described the proportion of individuals dispensed opioids at a specific time, and incidence outcomes measured the proportion of opioid-naïve individuals dispensed an initial opioid prescription within a time interval. Dosage outcomes assessed opioid potency per prescription, population, or time unit (e.g., morphine milligram equivalent [MME] units).^26^ Duration outcomes described the length of opioid supply per prescription or over a given time interval (e.g., number of days of supply in a year). Tapering/discontinuation outcomes measured dosage reductions, with qualitative review reserved for tapering due to variability in definitions across studies.

#### Study design

We included studies that employed an observational or quasi-experimental design (e.g., retrospective or prospective cohort studies, ecological studies, interrupted time series analyses, controlled or uncontrolled before–after studies, and repeated cross-sectional time series analyses), Eligible studies were required to provide repeated measures of these outcomes over time (i.e., time series data) sufficient to assess temporal trends before and after an intervention or policy change. We included studies that reported data as aggregate measures (e.g., prescription counts or rates) or individual-level summaries, provided they allowed for the reconstruction or extraction of time series information suitable for interrupted time series analysis.

#### Eligibility for qualitative systematic review

Studies were included in the qualitative systematic review if they (1) were published before October 14, 2025, and either (2a) estimated the effects of opioid prescribing guidelines (“guideline-specific studies”) or (2b) reported prescribing trends during guideline implementation (“non-guideline-specific studies”). We excluded preprints, abstracts, commentaries, systematic reviews, modeling studies, qualitative studies without quantitative data, case series studies, animal studies, and those focused on local interventions, prescription drug monitoring programs, opioid scheduling changes, drug take-back programs, or legal mandates limiting opioid dispensing. We excluded abstracts and articles at the full-text review stage published in a language other than English or French due to limited resources.

#### Eligibility for meta-analyses

Studies were eligible for inclusion in the meta-analysis if they reported an opioid prescribing outcome (e.g., prevalence, incidence, dosage, duration, or tapering/discontinuation) measured at eight or more distinct time points in a time series, with at least two measurements before and two after the implementation of the intervention (excluding the implementation date itself). For articles with overlapping study populations, we applied a predefined selection rule prioritizing studies with longer time series, unless insufficient pre- or postintervention observations warranted selection of an alternative study with an adequate number of time points. Studies from the same region were retained if they used different, nonoverlapping databases. Studies drawing on the same underlying databases were retained if they examined distinct outcomes, pain types, underlying conditions, or nonoverlapping time windows.

### Screening

Search records were imported into Covidence for duplicate removal and title and abstract screening. Two of six reviewers (M.S., I.W., J.L., I.F., P.T., and D.P.) independently screened titles and abstracts in Covidence,^27^ with conflicts resolved by consensus. Two of five reviewers (M.S., J.L., I.F., P.T., and D.P.) subsequently assessed full texts for inclusion based on predefined eligibility criteria. Records without full texts were excluded if authors could not be reached or were unable to provide full text.

### Data extraction

Two of four reviewers (M.S., J.L., I.F., and P.T.) independently extracted predefined qualitative information (e.g., study population, period, data sources, and design) and validated results. Two reviewers (P.T. and J.L.) abstracted quantitative data from studies (i.e., outcomes reported and measures of effect) and one of three reviewers (I.W., M.S., and D.P.) validated the extracted data. For meta-analysis, time series data were extracted directly from studies or requested from authors. If unavailable, data were digitized from figures using PlotDigitizer,^28^ with extracted points independently reviewed to minimize error.

### Quality assessment

We assessed the study quality using an adapted version of the “Quality Appraisal Checklist for Case Series Studies” from the Institute of Health Economics (see Table S3).^29^ Two of four reviewers (M.S., P.T., J.L., and D.P.) evaluated the quality of each study and resolved disagreements through consensus or adjudication by a third reviewer.

### Data analysis

#### Systematic review

For the systematic review, we visually assessed the extracted outcome measures to determine whether post-guideline trends were positive, negative, or unchanged relative to preintervention trends.

#### Interrupted time series analyses

For articles eligible for meta-analysis, we used interrupted time series (ITS) analysis to estimate immediate level and long-term trend changes in opioid prescribing outcomes after guideline implementation relative to the preintervention period. We applied segmented regression with outcome-specific distributions: negative binomial for counts (e.g., prescriptions dispensed), beta for proportions (e.g., percentage of patients dispensed opioids), and log-linear for dosage rates (e.g., MME per day). To account for autocorrelation, we calculated 95% confidence intervals using Newey-West standard errors,^30–32^ with a lag order equal to the number of time series measurements to the power of ¼, and otherwise specified lag orders based on assessment of residual autocorrelation.^33^

To standardize time, we centered the calendar time variable on the guideline implementation date, defined as the first day of the implementation year, quarter, or month. For studies that had intervals that exceeded 1 year, we conducted linear interpolation to estimate values at yearly time points. The intervention level was coded as 0 prior to and including the guideline implementation date and 1 post-guideline implementation. For each time series, we fit the following regression model:

$$\log\left(Y_{t}\right)=\beta_{0}+\beta_{1}\mathrm{tim}e_{t}+\beta_{2}\mathrm{leve}l_{t}+\beta_{3}\mathrm{tim}e_{t}\cdot \mathrm{leve}l_{t}+\varepsilon_{t}$$

Our outcomes of interest (*Y*) included annual measures of prevalence, incidence, dosage, and duration. In each model, β_1_ estimated the percentage change in the outcome over time before the intervention (preintervention trend). β_2_ estimated the immediate percentage change in the outcome level following the intervention (level effect), representing an abrupt increase or decrease occurring directly after the guideline implementation. β_3_ estimated the change in the postintervention trend relative to the preintervention trend (trend effect). When exponentiated, β_2_ and β_3_ represent percentage changes in the outcome level and trend, respectively. In other words, the level effect reflects an immediate shift in the outcome at the time of the implementation, whereas the trend effect reflects a gradual change in the trajectory of the outcome following the intervention.

#### Meta-analysis

We conducted meta-analyses by pain type (e.g., general/nonspecific, chronic, CNCP, acute, and cancer) if at least three studies reported the same outcome. Using study-specific ITS estimates, we calculated pooled immediate percentage changes (level effects) and percentage changes per year (trend effects) attributable to guidelines. Heterogeneity was assessed using the *I*^2^ and τ^2^ statistic. We reported random effects estimates derived from the restricted maximum likelihood method to account for the potentially high level of between-study heterogeneity (*I*^2^ > 70). We conducted all statistical analyses using R statistical software v4.3.2.^34^

## Results

### Guideline selection and characteristics

We identified 27 pertinent opioid prescribing guidelines for CNCP implemented between March 2007 and January 2022 (Table 1).^9–20,34–48^ These guidelines provided recommendations for the duration and dosage of opioid prescriptions as well as criteria for initiating, monitoring, and continuing therapy. Across all guidelines, there was a consistent emphasis on prioritizing nonopioid treatments and considering opioids only when other options have failed, basing opioid initiation on objective clinical evidence rather than continuation of previous prescriptions, implementing rigorous patient monitoring throughout therapy, and seeking specialist consultation or providing clear justification for high-dose regimens. Twenty-five out of 27 included guidelines reported a hard threshold (e.g., 90 MME/day, 200 MME/day, etc.) for reassessment, tapering, or discontinuation of opioid therapy.^9–20,34–37,39–41,43,45–48^ Sixteen guidelines were disseminated at the national level,^9,10,12–16,19,34,37,39,41–43,46,47^ whereas 11 were administered by authority groups at the provincial or state levels.^11,17,18,20,35,36,38,40,44,45,48^ Overall, the included guidelines originated from the United States (*n* = 9), Europe (*n* = 10), Canada (*n* = 4), and Australia/Asia (*n* = 4).

**Table 1.** Characteristics of included opioid prescribing guidelines for chronic noncancer pain.

Continent	Opioid prescribing guideline	Authority/author group	Abbreviation	Geographic jurisdiction	Implementation month	Key recommendations
North America	Canadian Guideline for Safe and Effective Use of Opioids for Chronic Non-Cancer Pain	National Opioid Use Guideline Group	NOUGG (2010)	Canada	June 2010	(1) Initiate opioid therapy only after thorough assessment and goal‑setting 2) <200 MME dose per day (3) Regular reevaluation of treatment and discontinuation of opioid treatment if benefit is insufficient or harms predominate
	The 2017 Canadian Guideline for Opioids for Chronic Non-Cancer Pain	Health Canada	Health Canada (2017)	Canada	May 2017	(1) Optimize nonopioid pharmacotherapies rather than immediately starting opioids (2) Maximum 90 MME/day (strong recommendation) but tapering to the lowest effective dose (or discontinuation) if already >90 MME/day (3) Advises against opioid initiation in patients with active substance use disorders or uncontrolled psychiatric comorbidity
	Douleur chronique et opioïdes: l′essentiel	Collège des médecins du Québec	CMQ (2009)	Quebec, Canada	May 2009	(1) Clear consent and patient engagement (2) Prioritization of nonopioid treatment but establishment of clear plan if opioid treatment becomes necessary (3) Rigorous monitoring and assessment of risks and benefits of opioid treatment (200 MME/day threshold)
	College of Physicians and Surgeons of British Columbia Safe Prescribing of Opioids and Sedatives Practice Standard	College of Physicians and Surgeons of British Columbia	CPSBC (2016)	British Columbia, Canada	June 2016	(1) Long-term opioid treatment based on a clear diagnosis, objective evidence, and comprehensive assessment (2) Doses >50 MME/day require caution; >90 MME/day requires strong justification along with close monitoring (3) Avoid high-risk and unsafe prescribing practices (e.g., coprescription of opioids and sedatives, prescription of large quantities of opioids)
	Clinical Guidelines for the Use of Chronic Opioid Therapy in Chronic Noncancer Pain	American Pain Society, American Academy of Pain Medicine	APS (2009)	United States	February 2009	(1) Rigorous patient selection with risks vs. benefits assessment (2) Treat opioid therapy as a trial, with informed consent and monitoring plan (3) Ongoing monitoring, reassessment, and action on escalation or risk (200 MME/day threshold)
	CDC Guideline for Prescribing Opioids for Chronic Pain	Centers for Disease Control and Prevention	CDC (2016)	United States	March 2016	(1) Prioritization of nonopioid and nonpharmacologic treatments (2) Special caution at or above 50 MME/day, with strong justification required at or above 90 MME/day (3) Minimization of opioids in higher risk patients (e.g., active substance use disorders or psychiatric comorbidities) while implementing tapering when needed
	Washington State Interagency Guideline on Opioid Dosing for Chronic Non-Cancer Pain	Washington State Agency Medical Director’s Group	WA (2007)	Washington	March 2007	(1) Warning threshold of 120 MME/day; pain management consultation for patients at or above this dose whose pain and function have not substantially improved (2) Regular assessment of safety and effectiveness by tracking sustained improvement in function and pain (3) Best practices for mitigating risk (e.g., opioid agreements and assessment of mental health and substance abuse history)
	Washington State Interagency Guideline on Opioid Dosing for Chronic Non-Cancer Pain (Update)	Washington State Agency Medical Director’s Group	WA (2010)	Washington	June 2010	(1) 120 MME/day dosage threshold, not to be exceeded unless the patient shows sustained improvement in both function and pain or a consultation with a pain management specialist is obtained (2) Opioid therapy as last resort after a trial of appropriate nonopioid medications, physical therapy, and behavioral therapies has failed (3) Mandatory monitoring for compliance (via urine drug testing) and efficacy along with explicit opioid agreement with the patient
	Assessment and Management of Chronic Pain	Institute for Clinical Systems Improvement	ICSI (2011)	Minnesota	November 2011	(1) Focus on improving patients’ function rather than solely eliminating pain via the biopsychosocial model (2) Physical rehabilitation and psychosocial management with functional goals before considering specialty referrals or more complex treatments (3) Careful patient selection and close monitoring for all chronic opioid patients, with consultation with a pain specialist for doses exceeding the suggested maximum of 200 MME/day
	Opioid Prescribing Guidelines for Treatment of Chronic, Non-Terminal Pain	Ohio Academy of Family Physicians	OAFP (2013)	Ohio	October 2013	(1) Prioritization of nonpharmacologic and nonopioid therapies; if opioids are considered, obtain informed consent, define functional goals, and check the state prescription monitoring database (2) 80 MME/day threshold, above which providers should reassess the treatment plan, review functional outcomes, reconsent, and consider specialist referral or alternative treatments (3) Avoidance of high-risk combinations (e.g., opioids with sedatives) without careful review; greater caution in patients with substance use or mental health risks
	Pennsylvania Guidelines on the use of Opioids to Treat Chronic Non-Cancer Pain	Pennsylvania Medical Society	PMS (2014)	Pennsylvania	2014	(1) Multimodal treatment, where opioids are rarely the sole treatment modality and are considered within the context of multimodal therapy (e.g., physical therapy and behavioral health) (2) Careful treatment consideration and consultation for specialty care when dose above the 100 MME/day threshold (3) Mandatory monitoring for efficacy, adverse events, and adherence and discontinuation if patients show no progress toward therapeutic goals or engage in repeated aberrant behaviors
	Massachusetts Medical Society Opioid Therapy and Physician Communication Guidelines	Massachusetts Medical Society	MA (2015)	Massachusetts	August 2015	(1) A thorough initial assessment, documented risk stratification (low, moderate, or high risk) of the patient, informed consent process, and opioid treatment agreement before initiating chronic opioid therapy (2) Consultation with a pain specialist before prescribing a daily dose that exceeds 100 MME/day or if the patient is categorized as high risk (3) Integration of multidisciplinary care by incorporating nonopioid therapies, physical therapy, behavioral health, and alternative strategies to improve function
	Prescribing Guidelines for Pennsylvania. Treating Chronic Non-Cancer Pain	Commonwealth of Pennsylvania	COP (2018)	Pennsylvania	June 2018	(1) Prioritize nonopioid/nonpharmacologic approaches and establish clear goals (2) Use of opioids only when clearly indicated, at the lowest effective dose, with rigorous monitoring (90 MME/day threshold) (3) Avoidance of high-risk prescribing combinations and mitigation of overdose risk
Europe	National Clinical Guideline on Opioid Treatment of Chronic Nonmalignant Pain	Danish Health Authority	DHA (2018)	Denmark	December 2018	(1) Prioritization of nonopioid treatments and use opioids only for selected patients (2) Restriction and careful monitoring of opioid dosage (not to exceed 100 MME/day, while considering limitation to 50 MME/day) (3) Avoidance of opioids in high-risk settings, while mandating reassessment or tapering when needed
	Opioid Use for the Management of Chronic Non-Cancer Pain: French Guidelines	Société Française d’Etude et Traitement de la Douleur	SFETD (2016)	France	June 2016	(1) Multimodal assessment and nonopioid treatment options before opioids; administration of this treatment modality only if the patient has a multimodal treatment failure and the expected benefit outweighs the risks (2) Discontinuation of opioid treatment after 3 months in the absence of clinically significant improvement in pain and quality of life (3) 150 MME/day dosage threshold and expert review recommended for dosage that exceeds this threshold
	German Pain Society Guideline, first edition	German Pain Society	GPS (2009)	Germany	September 2009	(1) Consideration of long-term opioid therapy only after failure of nonopioid and nonpharmacologic treatments and in patients with well-defined pain conditions likely to respond (e.g., osteoarthritis, chronic low back pain, or neuropathic pain) (2) Treatment initiation with the lowest effective dose, typically not exceeding 120 MME/day and longer than 6 months without documented functional improvement and regular specialist review (3) Strict monitoring of pain, function, side effects, and misuse risk and discontinuation if goals are unmet, tolerance develops, or adverse effects outweigh benefits
	German Pain Society Guideline, second edition	German Pain Society	GPS (2015)	Germany	January 2015	(1) Long-term opioid therapy is recommended for specific chronic pain conditions (e.g., osteoarthritis, lower back pain) but not for primary headaches and functional/psychiatric pain disorders (2) Mandatory efficacy review: Opioid therapy should be discontinued unless the patient shows significant pain reduction or functional improvement (3) Physicians are advised to adhere to 120 MME/day threshold; doses exceeding this threshold require special justification and expert consultation
	German Pain Society Guideline, third edition	German Pain Society	GPS (2020)	Germany	September 2020	(1) Restrictive indications (e.g., osteoarthritis, diabetic polyneuropathy, postherpetic neuralgia, and low back pain) and contraindications (e.g., primary headaches, functional somatic syndromes, and mental disorders) (2) Long-term therapy (defined as >26 weeks) is only justified if there is a positive therapy response; regular assessment of both clinical response and adverse effects to justify continuation of opioid treatment (3) Conservative dosing: Maintains the 120 MME/day threshold set in the previous version of this guideline
	Scottish Intercollegiate Guideline Network Management of Chronic Pain	Scottish Intercollegiate Guidelines Network, Healthcare Improvement Scotland	SIGN 136 (2013)	Scotland	December 2013	(1) Multimodal and biopsychosocial assessment to identify the pain type and functional impact as well as to inform the selection of the most effective treatment options (2) Prioritization of nonpharmacological strategies, including exercise and exercise therapies for the management of chronic pain (3) Specialist advice if the patient needs >180 MME/day or has concerns about rapid dose escalation, with patients being reviewed at least annually
	Guía de Consenso para el Buen Uso de Analgésicos Opioides	Socidrogalcohol	S-MS (2017)	Spain	June 2017	(1) Prioritization of nonopioid and nonpharmacologic treatments as first-line therapy and consideration of opioid analgesics only when the potential benefits (pain and function) clearly outweigh the risks (2) Prior to initiation, patients must receive comprehensive information about opioid therapy (including risks such as tolerance, hyperalgesia, addiction, overdose); structured treatment with defined functional goals, frequent reassessments, and a plan for dose reduction or discontinuation if outcomes are insufficient (3) Strict monitoring of dosage and risk factors: Close monitoring above 50 MME/day and clear justification in the patients’ medical records for doses >90 MME/day
	Guía de Atención a Los Pacientes con Dolor Crónico no Oncológico Utilizando Analgésicos Opioides	Departament de Salut. Generalitat de Catalunya	DPS (2018)	Catalonia, Spain	December 2018	(1) Long‑term opioid therapy for CNCP should only be considered after thorough assessment (2) Opioid prescriptions must be based on a clinical diagnosis and supported by objective evidence, not simply continuation of previous opioid prescriptions (3) Rigorous monitoring, including documentation of discussions with the patient regarding risks (including misuse and dependence) and readiness to adjust or discontinue therapy when benefit is inadequate
	Dolor Crónico no-Oncológico: ¿Opioides? INFAC	Osakidetza-Basque Health Service, Gobierno Vasco: Departamento de Salud	O-GV (2022)	Basque Country, Spain	January 2022	(1) Opioid medications should not be routine for CNCP due to uncertainty in long-term benefit and substantial risks (e.g., dependence, hyperalgesia, and adverse effects) (2) Optimization of nonopioid pharmacologic and nonpharmacologic therapies, by assessing psychological and social factors and setting function-based goals rather than just pain relief (3) Opioid use to be considered only for highly selected patients, with clear documentation of risks and benefits, ongoing monitoring of function versus harm, and readiness to stop if benefit is not achieved (with 50 MME/day and 90 MME/day thresholds)
	Opioids for Persistent Pain: Good Practice	British Pain Society	BPS (2010)	United Kingdom	January 2010	(1) Opioids should *not* be used as first-line therapy when evidence-based nonopioid and nonpharmacologic treatments are available (2) If there is no meaningful improvement (e.g., after reaching 120–180 MME/day), referral to specialist care is strongly recommended (3) Clinicians should assess for factors such as prior or current substance misuse, mental health disorders, and social context and ensure long-term monitoring is in place
Asia/Oceania	Faculty of Pain Medicine, Australian and New Zealand College of Anaesthetists Recommendations Regarding the Use of Opioid Analgesics in Patients with Chronic Noncancer Pain	Faculty of Pain Medicine, Australian and New Zealand College of Anaesthetists	Australia/New Zealand (2015)	Australia, New Zealand	December 2015	(1) Multimodal treatment and thorough assessments required, while prioritizing nonopioid treatments as first-line therapy (2) Caution at 40 MME/day with prompt reassessment and specialist advice at doses above 100 MME/day (3) Ongoing review, monitoring, and readiness to taper or cease opioid treatment
	A Guide to the Use of Strong Opioids in Chronic Non-Cancer Pain	Malaysian Association for the Study of Pain	MASP (2015)	Malaysia	August 2015	(1) Optimize nonopioid and interventional treatments, while limiting opioid treatment to being part of a multimodal treatment plan (2) A 3- to 4-week trial period for opioid therapy and discontinuation if no significant improvement in pain and function is observed after this period (3) Detailed documentation and monitoring of pain relief, activities of daily living (function), adverse effects, and aberrant drug-related behaviors
	Pain Association of Singapore: Evidence-Based Guidelines on the Use of Opioids in Chronic Non-Cancer Pain	Pain Association of Singapore	PAS (2013)	Singapore	March 2013	(1) Opioids are not recommended as first-line treatment for CNCP, but they may be used as second- or third-line therapy, preferably within a multimodal approach (maximum of 200 MME/day) (2) Careful screening and assessment of patients prior to starting opioids, with an opioid treatment agreement that may include urine drug testing (3) Trial duration of up to 2 months is necessary to determine efficacy of opioid treatment (e.g., pain relief, improvement in function and quality of life), with regular reviews to assess efficacy, adverse effects, and aberrant behavior
	Guidelines for Prescribing Opioids for Chronic Non-Cancer Pain in Korea	Korean Pain Society	KPS (2017)	South Korea	January 2017	(1) Prioritization of nonopioid and nonpharmacologic therapies (2) Opioid use based on clear diagnosis and reassessment, by relying on objective evidence, not continuation of past prescriptions, along with regular monitoring of pain, function, and risk (3) Careful reassessment of dosage at ≥50 MME/day and justification of exceptional benefit for doses ≥90 MME/day; tapering or discontinuation recommended if functional improvement is insufficient

### Study selection and characteristics

Our systematic search yielded 15,243 records, of which 1092 were assessed for eligibility in full-text review and 129 studies were ultimately retained. Further, we added 61 studies from a previous review commissioned by Health Canada and its internal validation (Figure 1). In total, we included 190 studies reporting opioid prescribing time series related to prescribing guidelines for the qualitative systematic review (Table S4) and 71 studies for the meta-analysis (Table 2). Studies included for systematic review reported on opioid prescribing in the United States (*n* = 146), Europe (*n* = 16), Canada (*n* = 13), and Australia/Asia (*n* = 12), with 3 studies focusing on multiple countries.

**Table 2.** Characteristics of studies included in the meta-analysis (*n* = 71).

Study	Location	Period of observation	Guideline^a^	Pain type	Data source	Outcomes	Time series length (pre, post)	Unit of measurement
Adalbert et al.^165^	Pennsylvania	2016–2020	COP (2018)	General	Pennsylvania prescription drug monitoring programs	Number of prescriptions	15 (7, 7)	Quarter
Adewumi et al.^59^	Queensland, Australia	1997–2018	ANZCA (2015)	General	Monitoring of Drugs of Dependence system	Number of prescriptions, prevalence (%)	22 (18, 3)	Year
Ahn et al.^137^	South Korea	2010–2022	KPS (2017)	Chronic	National Health Insurance Sharing Service–National Health Information Database of South Korea	Prevalence (%)	13 (7, 5)	Year
Ali et al.^168^	United States	2005–2015	APS-AAPM (2009)	General	IBM MarketScan Commercial Claims and Encounters Database, Multi-State Medicaid Database	Prevalence (*N*)	11 (4, 6)	Year
Aubry et al.^75^	United States	2010–2019	CDC (2016)	General	Drug Overdose Deaths (National); Total Overdose Deaths, Any Opioid Overdose Deaths and Prescription Opioid Overdose Deaths	MME per person	10 (6, 3)	Year
Bandara et al.^49^	United States	2012–2019	CDC (2016)	CNCP, cancer	IBM MarketScan Research Databases	Prevalence (%)	8 (4, 3)	Year
Bedson et al.^216^	United Kingdom	2002–2012	BPS (2010)	General	CPRD	Number of prescriptions	11 (8, 2)	Year
Bhattacharya et al.^84^	United States	2012–2020	CDC (2016)	General	Medicare	MME per day, duration, incidence (%)	8 (3, 4)	Year
Bohnert et al.^21^	United States	2012–2017	CDC (2016)	General	IQVIA transactional data warehouse	MME per person, duration	72 (50, 21)	Month
Brett et al.^72^	Australia, New Zealand, United States, Canada, United Kingdom, Denmark, South Korea	2000–2020	ANZCA (2015); APS-AAPM (2009); CDC (2016); Health Canada (2017); BPS (2010); DHA (2018); KPS (2017)	General, CNCP	Australia: Administrative claims data, Pharmaceutical Benefits Scheme data New Zealand: Ministry of Health National Collections, Pharmaceutical Collection United States: Merative Marketscan Commericial Claims and Encounters, Medicaid Canada: Institute for Clinical Evaluative Sciences database United Kingdom: CPRD GOLD database Denmark: Central Registration of Medication Use South Korea: Health Insurance Review and Assessment Database	Prevalence (%)	Australia: 7 (2, 4) New Zealand: 16 (11, 4) U.S. private: 10 (6, 3); 8 (3, 4) U.S. public: 19 (9, 9) Canada: 8 (4, 3) UK: 16 (5, 10) Denmark: 20 (17, 2) South Korea: 11 (7, 3)	Year
Canizares et al.^153^	Ontario, Canada	2004–2017	NOUGG (2010)	Acute	Ontario administrative data	Incidence (%)	14 (6, 7)	Year
Casagrande et al.^60^	United States	2008–2019	CDC (2016)	General	Medicare	Prevalence (%)	12 (8, 3)	Year
Chai et al.^76^	United States	1997–2015	APS-AAPM (2009)	General	IQVIA National Prescription Audit	Number of prescriptions, MME per person	19 (12, 6)	Year
Champagne-Langabeer et al.^173^	United States	2015–2017	CDC (2016)	General	Texas Prescription Monitoring Program	Number of prescriptions	12 (5, 6)	Quarter
Chen et al.^50^	United States	2003–2014	APS-AAPM (2009)	CNCP	Truven MarketScan database	Prevalence (%)	10 (5, 4)	Year
Cohen et al.^51^	United States	2000–2019	APS-AAPM (2009)	CNCP	Women’s Interagency HIV Study	Prevalence (%)	20 (9, 10)	Year
Crabtree et al.^61^	British Columbia, Canada	2015–2017	CPSBC (2016)	General	British Columbia administrative data	Number of prescriptions, prevalence (%), incidence (%), MME per day	30 (17, 12)	Month
Curtis et al.^77^	United Kingdom	1998–2017	BPS (2010)	General	NHS Digital	MME per person	20 (12, 7)	Year
Dart et al.^176^	United States	2002–2013	APS-AAPM (2009)	General	RADARS System Programs	Number of prescriptions	32 (12, 19)	Quarter
Davies et al.^62^	Wales	2005–2015	BPS (2010)	General	SAIL Databank	Prevalence (%)	11 (5, 5)	Year
Day et al.^161^	United States	2010–2020	CDC (2016)	Acute	PearlDiver Mariner91 national administrative claims database	MME per day	11 (6, 4)	Year
DiPrete et al.^214^	United States	2010–2018	CDC (2016)	General	MarketScan	Incidence (*N*)	9 (6, 2)	Year
Franklin et al.^180^	United States	1996–2010	WA (2007)	General	Washington State Department of Labor Industries Medical Information Payment System	MME per day	178 (134, 43)	Month
Garcia et al.^213^	Massachusetts	2002–2017	APS-AAPM (2009)	General	MassHealth pharmacy claims	MME per day	61 (28, 32)	Quarter
Garg et al.^64^	Washington	2004–2010	WA (2007)	General	Washington State Department of Labor Industries Medical Information Payment System	Prevalence (%)	27 (12, 14)	Quarter
Gomes et al.^78^	Canada	2008–2016	NOUGG (2010)	General	IQVIA Canada Compuscript	MME per person	95 (25, 69)	Month
Goudman et al.^128^	United States	2012–2022	CDC (2016)	Chronic	Electronic health records from TriNetX	Prevalence (%), incidence (%)	11 (4, 6)	Year
Gupta et al.^183^	United States	2015–2017	CDC (2016)	General	UnityPoint Health Records System	Number of prescriptions	36 (14, 21)	Month
Guy et al.^82^	United States	2006–2015	APS-AAPM (2009)	General	QuintilesIMS Transactional Data Warehouse	MME per day, duration	10 (3, 6)	Year
Han et al.^129^	United States	2006–2015	APS-AAPM (2009)	Chronic	Medicare	Prevalence (%)	10 (3, 6)	Year
Hauser et al.^57^	United States, Canada, Germany	1980–2014	APS-AAPM (2009); NOUGG (2010); GPS (2009)	CNCP	International Narcotics Control Board	MME per person	35 (30, 4)	Year
Hebert et al.^22^	United Kingdom	2005–2020	SIGN (2013)	General	Public Health Scotland Prescribing Information System	Number of prescriptions	184 (107, 76)	Month
Hechter et al.^185^	California	2013–2020	CDC (2016)	General	Kaiser Permanente Southern California electronic health record databases	Prevalence (*N*), incidence (*N*)	8 (3, 4)	Year
Hedenmalm et al.^79^	Germany	2006–2016	GPS (2009)	General	IMS Disease Analyzer	MME per person, duration	11 (3, 7)	Year
Holmer et al.^117^	United States	2012–2020	CDC (2016)	Chronic	VA Corporate Data Warehouse	Prevalence (%)	9 (4, 4)	Year
Hu et al.^81^	United States	2011–2021	CDC (2016)	General	U.S. DEA Automated Reports and Consolidated Ordering System	MME per person	44 (20, 23)	Quarter
Kang et al.^23^	United States	2011–2019	CDC (2016)	CNCP	Merative MarketScan Commercial Database	MME per person, duration	108 (62, 45)	Month
Kazanis et al.^189^	United States	2006–2013	APS-AAPM (2009)	General	IMS Health National Prescription Audit and Vector One: National	Number of prescriptions	8 (3, 4)	Year
Kimmel et al.^121^	United States	2011–2020	CDC (2016)	Chronic	U.S. Renal Data System, Medicare	Prevalence (%)	10 (5, 4)	Year
Lee et al.^130^	United States	2002–2015	APS-AAPM (2009)	Chronic	Corrona RA Registry	Prevalence (%)	14 (7, 6)	Year
Leja et al.^138^	Michigan	2015–2018	CDC (2016)	Chronic	Saint Joseph Mercy hospital HER	Incidence (%)	41 (14, 26)	Month
Liu et al.^65^	United States	2013–2021	CDC (2016)	General	Medicare	Prevalence (%)	9 (3, 5)	Year
Ly et al.^73^	Quebec, Canada	1997–2018	CMQ (2009)	General	RAMQ, MED-ECHO	Prevalence (%)	11 (6, 4)	Year
Maierhofer et al.^58^	United States	2012–2018	CDC (2016)	CNCP, acute	Private health insurance claims data	MME per day, duration	72 (50, 21)	Month
Mauck et al.^151^	United States	2001–2020	APS-AAPM (2009)	Acute	IBM MarketScan	Incidence (%)	19 (8, 10)	Year
Mojtabai^66^	United States	1999–2014	APS-AAPM (2009)	General	NHANES	Prevalence (%)	15 (10, 4)	Year
Nahin et al.^67^	United States	1997–2014	APS-AAPM (2009)	General	Medical Expenditure Panel Survey	Prevalence (%)	17 (12, 4)	Year
Panagiotoglou et al.^24^	British Columbia, Canada	2012–2020	CPSBC (2016)	CNCP, cancer, palliative	BC administrative data	MME per person	84 (41, 42)	Month
Pensa et al.^68^	United States	2003–2013	APS-AAPM (2009)	General	Claims data on an industrial cohort from a specialty metals corporation with multiple manufacturing facilities geographically dispersed throughout the United States	Prevalence (%)	11 (6, 4)	Year
Pritchard et al.^52^	United States	2011–2019	CDC (2016)	CNCP, acute	Medical Expenditure Panel Survey	Prevalence (%)	9 (5, 3)	Year
Rancu et al.^147^	United States	2011–2021	CDC (2016)	Acute	PearlDiver Mariner 161 Insurance Database	Prevalence (%), MME per person	11 (5, 5)	Year
Rancu et al.^148^	United States	2011–2021	CDC (2016)	Acute	PearlDiver Mariner 161 Insurance Database	Prevalence (%), MME per person	11 (5, 5)	Year
Richter et al.^136^	Minnesota	2005–2015	APS-AAPM (2009)	Chronic	Retrospective data from Rochester Epidemiology Project	Prevalence (%)	11 (4, 6)	Year
Rogal et al.^124^	United States	2005–2014	APS-AAPM (2009)	Chronic	VA Corporate Data Warehouse	Prevalence (%)	10 (4, 5)	Year
Rolova et al.^74^	Denmark	2010–2023	DHA (2018)	General	Public Prescription Drug Statistics	Prevalence (%), MME per day	14 (8, 5)	Year
Salvatore et al.^200^	United States	2015–2019	CDC (2016)	General	IQVIA Longitudinal Prescription database	Number of prescriptions, MME per day	60 (14, 45)	Month
Schieber et al.^83^	United States	2006–2017	APS-AAPM (2009)	General	IQVIA Xponent database	Number of prescriptions, duration	12 (3, 8)	Year
Schieber et al.^91^	United States	2008–2018	CDC (2016)	General	IQVIA Total Patient Tracker	Prevalence (%)	11 (8, 2)	Year
Scott et al.^125^	North Staffordshire, UK	2000–2015	BPS (2010)	Chronic	Consultations in Primary Care Archive	Prevalence (%)	16 (10, 5)	Year
Sears et al.^70^	Washington	2008–2015	WA (2010)	General	Workers’ Compensation data	Prevalence (%)	48 (29, 18)	Month
Sears et al.^69^	Washington	2012–2017	CDC (2016)	General	Washington State Prescription Monitoring Program data	Prevalence (%, *N*), incidence (%, *N*)	24 (17, 6)	Quarter
Shen et al.^53^	United States	2006–2013	APS-AAPM (2009)	CNCP	Medicare Current Beneficiary Survey	Prevalence (%)	8 (3, 4)	Year
Smith et al.^203^	Illinois	2009–2018	CDC (2016)	General	Northwestern Medicine Enterprise Data Warehouse	Prevalence (%)	10 (7, 2)	Year
Smolina et al.^80^	British Columbia, Canada	2005–2013	NOUGG (2010)	General	British Columbia administrative data	MME per person	9 (5, 3)	Year
Song et al.^89^	South Korea	2010–2019	KPS (2017)	CNCP	National Health Insurance Service (NHIS) database	Prevalence (%)	10 (7, 2)	Year
Stokes et al.^54^	United States	1999–2016	APS-AAPM (2009)	CNCP	NHANES	Prevalence (%)	17 (10, 6)	Year
Surbhi et al.^105^	United States	2004–2018	APS-AAPM (2009); CDC (2016)	CNCP	U.S. Renal Data System	Incidence (%)	15 (12, 2)	Year
Taqi et al.^3449^	United Kingdom	2000–2014	BPS (2010)	General	CPRD	MME per person	15 (10, 4)	Year
Whitney et al.^127^	United States	2011–2017	CDC (2016)	Chronic	Optum Clinformatics Data Mart Database	Prevalence (%)	81 (59, 21)	Month
Zamora-Legoff et al.^133^	Minnesota	2005–2014	APS-AAPM (2009)	Chronic	Rochester Epidemiology Project	Prevalence (%)	10 (4, 5)	Year
Zhu et al.^71^	United States	2012–2017	CDC (2016)	General	Blue Cross Blue Shield Axis claims database	Prevalence (%), incidence (%), MME per day	66 (44, 21)	Month

^a^See Table 1, Abbreviation: ANZCA = Australian and New Zealand College of Anaesthetists; APS-AAPM = American Pain Society-American Academy of Pain Medicine; BPS = British Pain Society; CDC = Centers for Disease Control and Prevention; CNCP = Chronic non-cancer pain; COP = Commonwealth of Pennsylvania; GPS = German Pain Society; KPS = Korean Pain Society; MME = Morphine milligram equivalent; NOUGG = National Opioid Use Guideline Group; SIGN = Scottish Intercollegiate Guideline Network; S-MS = Socidrogalcohol-Ministerio de Sanidad ; UK = United Kingdom; USA = United States of America.

Most reported time series overlapping with the CDC12 (*n* = 90) and the American Pain Society-American Association of Pain Medicine (APS-AAPM)^10^ guideline implementation dates (*n* = 59), with additional studies reporting time series overlapping with the Canadian National Opioid Use Guideline Group (NOUGG; *n* = 9)^14^ and Australian/New Zealand (*n* = 8).^13^ Among the articles included for the qualitative systematic review (Table S4), the majority of studies examined opioid dispensation in the general population (*n* = 97), followed by CNCP (*n* = 34), acute pain (*n* = 29), chronic pain (*n* = 26), and cancer pain (*n* = 9) populations.

### Quality assessment

Of the 190 studies included for the systematic review, 26 were guideline-specific studies and 164 were non-guideline-specific studies. Upon quality assessment, we observed that 159 studies had a clearly stated objective, 144 had a clearly defined study population, 185 defined outcome measures a priori (i.e., prespecified), and 163 presented study conclusions that were consistent with the results. Through examination of disclosure and ethics statements, we identified 147 studies that explicitly stated both competing interests and financial support and 143 studies that explicitly stated ethics approval or exemption (Supplementary Materials Table S5).

### Meta-analyses

#### Chronic noncancer pain

**Figure 2.**
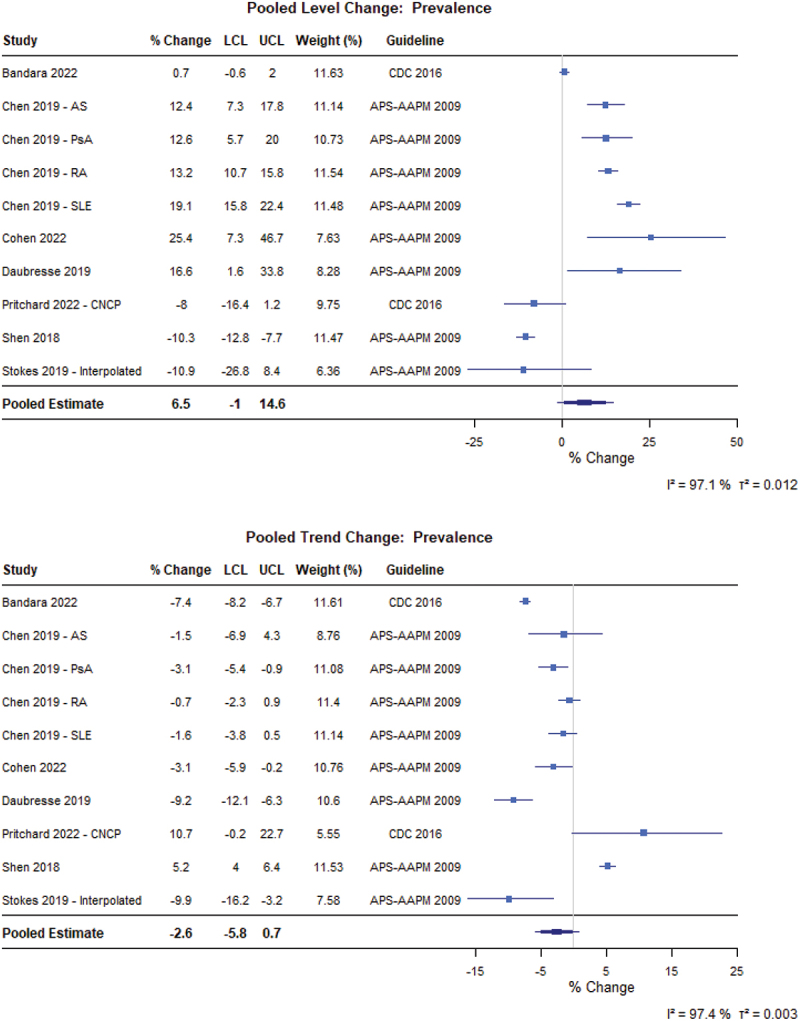
Forest plot of the percentage changes in level and trend for the prevalence of patients with CNCP prescribed opioids following guideline implementation. APS-AAPM = American Pain Society-American Association of Pain Medicine; AS = ankylosing spondylitis; CDC = Centers for Disease Control and Prevention; CNCP = Chronic non-cancer pain; LCL = Lower confidence limit; PsA = psoriatic arthritis; RA = rheumatoid arthritis; SLE = systemic lupus erythematosus; UCL = Upper confidence limit.

**Figure 3.**
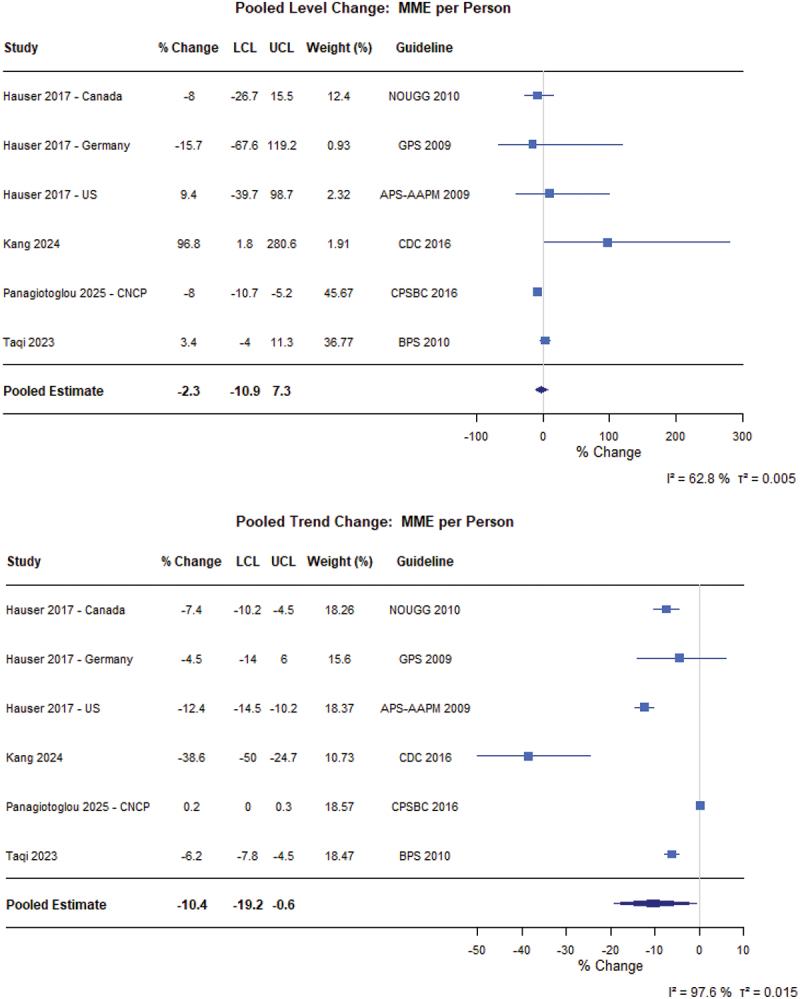
Forest plot of the percentage changes in level and trend for the MME dose per person in the chronic noncancer pain population following guideline implementation. APS-AAPM = American Pain Society-American Association of Pain Medicine; BPS = British Pain Society; CDC = Centers for Disease Control and Prevention; GPS = German Pain Society; LCL = Lower confidence limit; MME = Morphine milligram equivalent dose; NOUGG = National Opioid Use Guideline Group; UCL = Upper confidence limit; US = United States.

**Figure 4.**
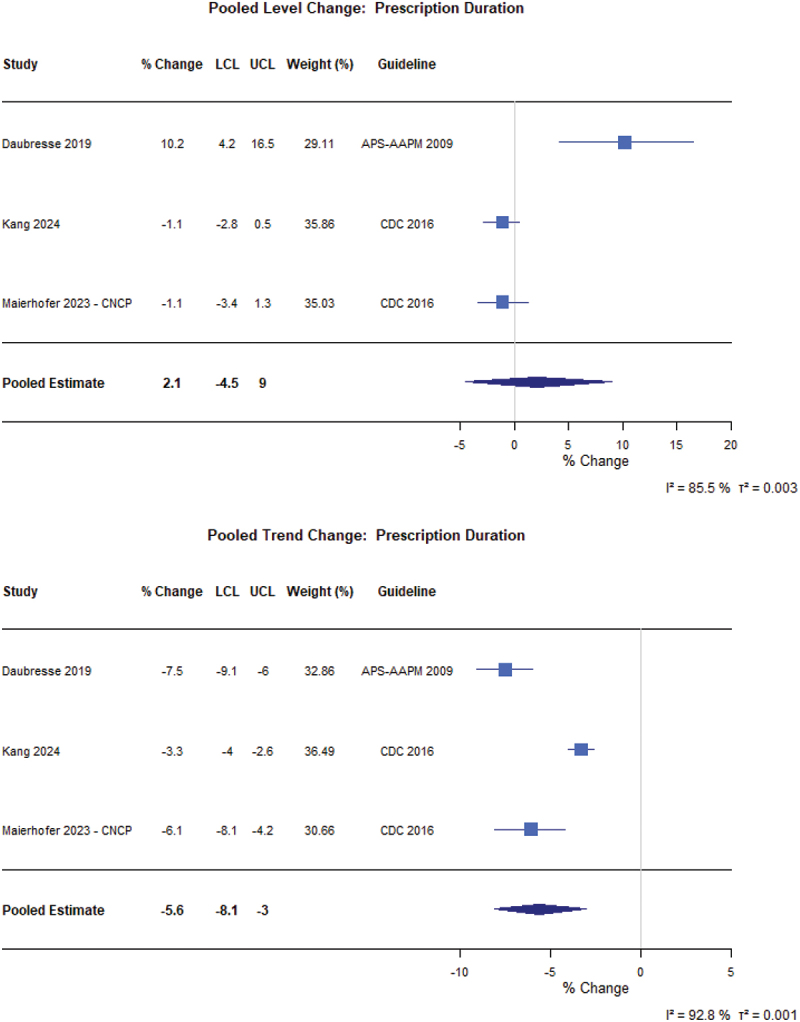
Forest plot of the percentage changes in level and trend for the average duration of opioid prescriptions in patients with CNCP following guideline implementation. APS-AAPM = American Pain Society-American Association of Pain Medicine; CDC = Centers for Disease Control and Prevention; LCL = Lower confidence limit; UCL = Upper confidence limit.

For patients with CNCP (Figure 2), guidelines did not have an immediate level effect (6.5%, 95% confidence interval [CI] −1.0% to 14.6%) or trend effect (−2.6%, 95% CI −5.8% to 0.7%) on the prevalence of people prescribed opioids (*N* = 7 from 10 different CNCP populations).^49–55^ Similarly, there was no meaningful immediate level change in MME per person (−2.3%, 95% CI −10.9% to 7.3%), but there was a 10.4% decrease in the yearly trend (95% CI −19.2% to −0.6%; *N* = 4 from 6 different patient populations; Figure 3).^23,24,56,57^ Finally, there was no level change in prescription duration (2.1%, 95% CI −4.5% to 9.0%) immediately following implementation, but there was a 5.6% decrease in prescription duration year to year (95% CI −8.1% to −3.0%; *N* = 3) following guideline implementation (Figure 4).^23,55,58^

#### General population

**Figure 5.**
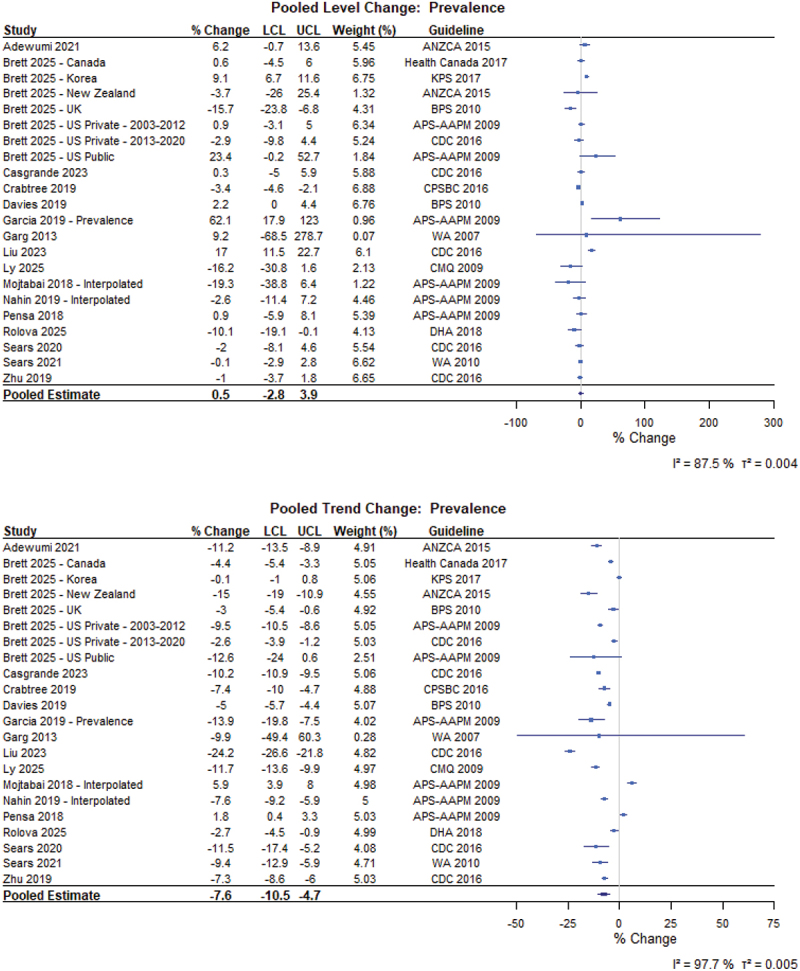
Forest plot of the percentage changes in level and trend for the prevalence of patients in the general population prescribed opioids following guideline implementation. APS-AAPM = American Pain Society-American Association of Pain Medicine; ANZCA = Australian and New Zealand College of Anaesthetists; BPS = British Pain Society; CDC = Centers for Disease Control and Prevention; CMQ = Collège des médecins du Québec; CPSBC = College of Physicians and Surgeons of British Columbia; DHA = Danish Health Authority; KPS = Korean Pain Society; LCL = Lower confidence limit; NOUGG = National Opioid Use Guideline Group; UCL = Upper confidence limit; WA = Washington state.

**Figure 6.**
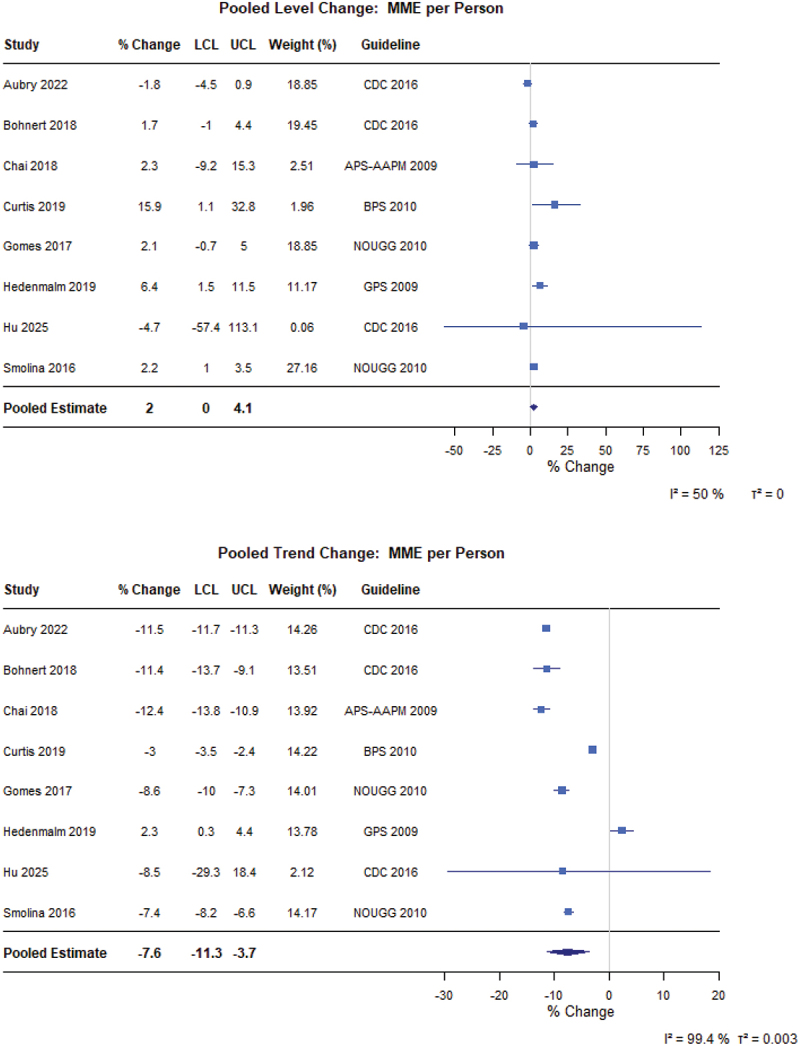
Forest plot of the percentage changes in level and trend for the MME dose per person in the general population following guideline implementation. APS-AAPM = American Pain Society-American Association of Pain Medicine; BPS = British Pain Society; CDC = Centers for Disease Control and Prevention; GPS = German Pain Society; LCL = Lower confidence limit; NOUGG = National Opioid Use Guideline Group; UCL = Upper confidence limit; WA = Washington state.

**Figure 7.**
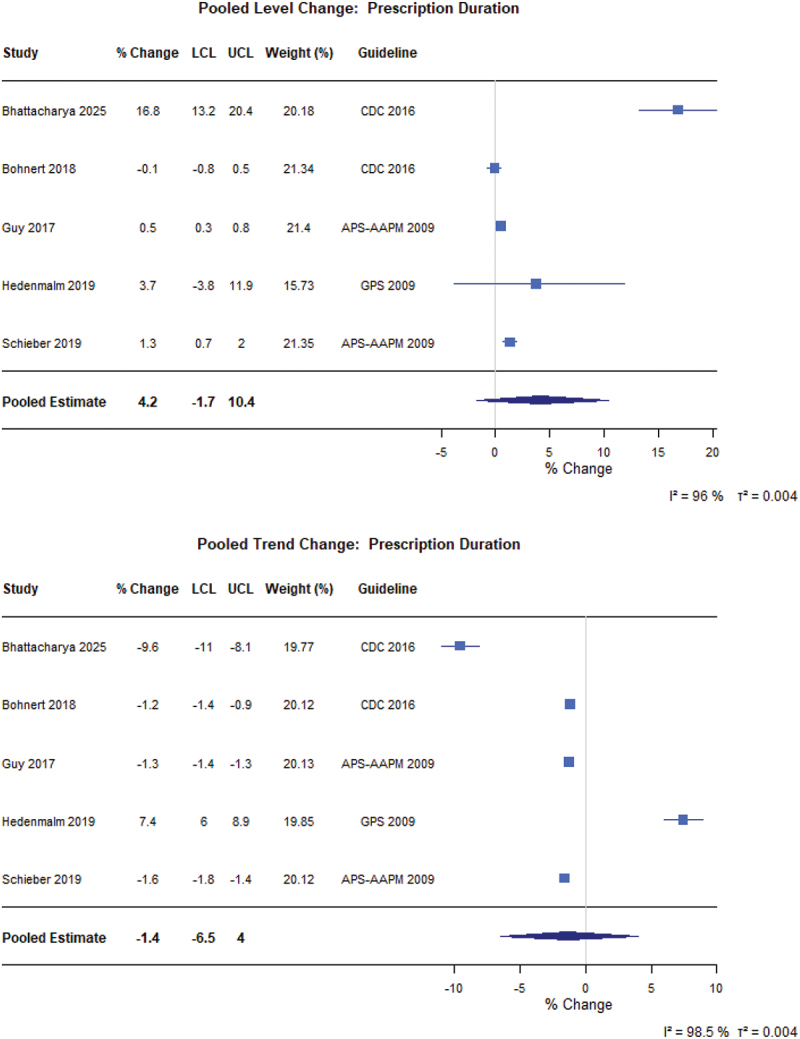
Forest plot of the percentage changes in level and trend for the average duration of opioid prescriptions in the general population following guideline implementation. APS-AAPM = American Pain Society-American Association of Pain Medicine; CDC = Centers for Disease Control and Prevention; GPS = German Pain Society; LCL = Lower confidence limit; UCL = Upper confidence limit.

For the general population (Figure 5), there was no immediate level change (0.5%, 95% CI −2.8% to 3.9%) but an 7.6% yearly trend decrease (95% CI −10.5% to −4.7%) in the prevalence of opioid use following guideline implementation (*N* = 16 from 22 different populations).^59–74^ There was limited level change (2.0%, 95% CI 0.0 to 4.1) but a 7.6% yearly trends (95% CI −11.3% to −3.7%) in MME per person (*N* = 8; Figure 6).^21,75–81^ Further, we did not find level or trend changes for the duration of opioid prescriptions following guideline implementation (*N* = 5; Figure 7).^21,79,82–84^ All other meta-analytic findings, including incident opioid use, MME per day, number of prevalent users, and number of opioid prescriptions, are presented in the Figures S2 to S7.

### Qualitative synthesis

Findings from the qualitative synthesis are presented in Table S6.

#### Chronic noncancer pain

The effect of the guidelines on prevalence remains unclear, with nine studies reporting an increasing trend^52,53,85–91^ and nine studies reporting a decreasing trend.^23,49,91–97^ Four studies reported no changes or post-guideline prevalence that varied depending on the specific guideline examined (e.g., APS-AAPM versus CDC).^50,54,96,98^ However, the guidelines’ effects on the incidence were clearer, with seven studies showing a decrease,^87,90,99–103^ two studies showing no trend changes,^104,105^ and two studies showing an increase in prescribing incidence trend.^106,107^ As for dosage, four studies described a decreasing trend,^23,24,49,108^ two studies described an increasing trend,^56,109^ and two studies described no change^58,110^ following implementation. Five studies found a decline in the prescription duration trend post-guideline implementation.^23,49,58,90,106^ Three studies showed an increasing trend for tapering and discontinuation of opioid treatment, including abrupt or rapid discontinuation.^111–113^ One study showed limited changes in opioid discontinuation among patients undergoing long-term opioid treatment,^114^ and one study showed a decreasing post-guideline trend for aggressive tapering.^24^

#### Chronic pain

For studies focusing on chronic pain patients, 13 showed decreasing trends,^115–127^ 6 showed increasing trends,^128–133^ and 4 showed no changes in the prevalence following guideline implementation.^134–137^ Two studies observed that the proportion of patients dispensed their first opioid prescription decreased after guideline implementation,^122,138^ with one study observing an increase in incident prescriptions.^128^ Related, three studies demonstrated decreasing post-guideline trends in dosage prescribed.^55,126,139^ One study demonstrated decreasing post-guideline trends in the duration of opioid prescriptions,^55^ and another post-guideline trend.^129^

#### Acute pain

In the acute pain population, 10 studies (with 11 outcome measures) showed a decreasing trend,^119,140–148^ 2 showed an increasing trend,^52,149^ and 3 showed no change^97,98,150^ in prevalence following guideline implementation. Two studies found there was a declining trend in opioid use incidence,^151,152^ and another study found no trend changes following guideline implementation.^153^ Meanwhile, 14 studies reported decreases in dosage trends following guideline rollouts,^58,141,147,148,150,152,154–161^ with one study showing an increase in total MME per prescription.^141^ With respect to prescription duration, 3 studies showed decreasing trends,^58,154,160^ whereas 2 described an increase in duration over time.^141,162^

#### General population

For studies reporting prevalence outcome measures,^22,51,59–63,65–74,77,163–213^ 38 outcome measures showed negative trends, 26 showed positive trends, 8 showed no change, and one showed differing prevalence by country and database. The guideline effect on incident opioid use remains less clear. Six studies reported a decrease,^71,84,209,214–216^ 3 studies showed no change,^61,185,217^ one study showed an increase,^218^ and one study showed incident prescribing trends varied by database.^219^ For dosage, 18 studies reported decreasing trends,^21,63,71,74,75,81,82,84,165,169,200,209,211,220–224^ whereas 7 studies reported an increasing trend in dosage.^57,77,79,80,210,225,226^ Four studies showed trend changes in dosage that varied by guideline, analgesic type, or database.^78,219,227,228^ Of the 8 studies reporting duration of prescriptions dispensed, 2 observed an increasing trend post-guideline,^71,79^ 4 observed a decreasing trend post-guideline,^21,84,169,229^ and 2 observed no changes.^82,230^ One study showed an increasing post-guideline trend in abrupt discontinuation but a decreasing post-guideline trend in tapered discontinuation.^231^

#### Cancer pain

Four studies reported a trend decrease in the proportion of individuals receiving opioid prescriptions,^49,87,232,233^ with two studies reporting no changes^188,234^ and one study indicating an increase in the mean number of opioid prescriptions per person.^49^ Two studies reported no changes in the proportion of incident opioid use post-guideline implementation.^87,235^ For dosage, three studies showed a decreasing trend,^24,49,232^ and one showed no changes.^110^ For duration, two studies reported a decrease in trend following guidelines.^49,232^

#### Palliative pain

One study showed an increasing post-guideline trend for opioid prescription claims among nononcologists,^188^ and another study showed a decreasing post-guideline trend for MME per person.^24^

## Discussion

This systematic review and meta-analysis consolidated evidence on the impact of opioid prescribing guidelines across jurisdictions and populations. Our findings reveal year-to-year declines in opioid prescription prevalence, incidence, and dosage following guideline implementation. More specifically, among patients with CNCP, the primary target group, the guidelines led to decreasing year-to-year trends in MME per person and prescription duration. Similarly, in the general population (i.e., guideline exempt), there were declining annual trends in the prevalence of opioid use and MME per person as well as MME per day and number of opioid prescriptions. The results from the qualitative synthesis demonstrate that the majority of studies across acute pain, chronic pain, and cancer pain populations reported decreasing trends in the prevalence, dosage, and duration of opioid prescriptions following guideline implementation.

Opioid prescribing guidelines contributed to shifts in opioid prescribing within clinical practice, aligning with public health goals to mitigate access to opioids. This shift likely reflects heightened awareness of opioid risks and increased use of alternative pain management strategies, such as nonopioid analgesics (e.g., gabapentinoids, paracetamol) and multimodal approaches incorporating psychosocial and personalized care.^92,236–238^ However, these changes in opioid prescribing (e.g., dose reduction, lower prevalence, and lower incidence) may also reflect the unintended consequences of the guidelines.^239,240^ In the general population, significant decreases in opioid prevalence and prescriptions were observed, but this may have been due to abrupt tapering and discontinuation of opioid treatments along with patients being denied opioids.^239^ Additionally, declining trends in opioid dosage for patients with acute pain and prevalence among patients with cancer further suggest spillover effects on guideline-exempt populations. From a policy standpoint, these findings indicate that prescribing guidelines can meaningfully influence clinical practice, but they also underscore the need for greater nuance in guideline design and implementation. Future guidelines should balance opioid stewardship with clinical flexibility by incorporating safeguards against spillover effects in guideline-exempt populations and emphasizing patient-centered outcomes beyond prescribing volume alone.

The findings from the meta-analysis warrant careful interpretation, because most of the included studies were conducted in the United States. Consequently, the effect estimates predominantly reflect the level and trend changes associated with the implementation of the CDC guidelines and the APS-AAPM guidelines.^10,12^ Importantly, pre-guideline opioid prescribing trends in the United States differed from those observed in other countries. For instance, in North America, a decline in opioid prescriptions began in the early 2010s, preceding the introduction of many guidelines in the mid-2010s.^82,140,241,242^ In contrast, countries such as Australia, the United Kingdom, and Germany experienced increasing trends in opioid dispensing and prescribing during the same period.^56,77,243–245^ As a result, the impact of guidelines on clinical practice in the United States may have been less pronounced, because the decline in opioid prescribing was already underway due to increased awareness of potential risks associated with opioids.^61,82^ However, by fostering a climate of caution, the guidelines likely reinforced or accelerated this downward trend, pushing many physicians away from opioid-based treatments. At the same time, the guidelines appear to have prompted a significant shift in opioid initiations, resulting in fewer new patients being prescribed opioids and reductions in both dosage and prescription duration. This shift reflects a broader change in clinical practice paradigms, moving away from reliance on prescription opioids.^238^

Variations in the observed effects may also reflect differences in implementation fidelity and contextual factors across jurisdictions. For instance, the APS-AAPM guideline^10^ was published before the onset of the opioid crisis in the United States and endorsed chronic opioid therapy as a potentially effective pain management strategy.^10^ In contrast, the CDC guideline,^12^ developed in response to the escalating crisis, adopted a more restrictive and cautious approach, specifying dosage and duration limits and recommending nonopioid treatments whenever possible.^12^ A similar evolution occurred in Canada: the NOUGG predated the national opioid crisis and emphasized risk management and the safe and effective use of opioids,^14^ whereas the College of Physicians and Surgeons of British Columbia (CPSBC) practice standard and Health Canada guideline emerged as crisis-driven responses and closely mirrored the CDC’s restrictive recommendations.^9,11^ Notably, the CPSBC practice standard was legally enforceable, demanding a higher level of implementation fidelity than earlier advisory guidelines. In North American contexts, therefore, guidelines from the 2009–2010 period reflected clinical frameworks for appropriate long-term opioid use, whereas those introduced from 2016 onward represented public health–driven interventions aimed at minimizing opioid exposure. These temporal, regulatory, and contextual differences along with variation in the degree to which guidelines were implemented likely contributed to the heterogeneity in observed effects across studies and populations.

### Limitations

Our study has several limitations. First, our database search focused on prescribing practices and patterns, the primary target of guidelines, capturing opioid prescription volumes rather than opioid use, potentially missing eligible studies. With guidelines’ intended audience being prescribers, we assumed a reduction in prescription volume led to a reduction in opioid use. To mitigate this concern, we consulted a librarian to validate our search strategy in January 2023 for the initial review commissioned by Health Canada, repeated the search in May 2024, and updated it in January 2025 and October 2025. Second, some studies lacked clear mention of prescribing outcomes in their titles or abstracts, complicating eligibility assessment. Internal validation helped identify and reinclude originally excluded studies. Third, most studies focused on opioid prescribing in the general population, limiting insight into how CNCP pain guidelines affected target and exempt populations, separately and specifically. Additionally, the lack of studies on certain outcomes or pain types prevented comprehensive meta-analysis across all patient groups. To address this, we qualitatively synthesized guideline effects to guide future evaluations. Fourth, plot digitization for time series data extraction may have introduced measurement bias. To minimize this bias, all plot digitized figures were independently reviewed and validated by an additional reviewer. Fifth, some papers using aggregate-level data were not clear in their included age range. Assuming a very low percentage of pediatric patients with opioid prescriptions in studies covering the entire population, we treated these studies as adult population studies. Sixth, we acknowledge the influence of concurrent policy changes and co-interventions across jurisdictions. During the study periods, some jurisdictions implemented multiple, overlapping initiatives alongside the prescribing guidelines, such as restrictions on high-dose formulations, prescription monitoring programs, and public awareness campaigns.^246–249^ Admittedly, these co-interventions make it challenging to isolate the specific causal effect attributable solely to guideline implementation. However, most interventions were not concurrent, with interventions like narcotics monitoring systems predating guideline rollouts by years. Though some spillover effects of other interventions are likely captured, we consider this study an ecologically valid measure of the real-world changes driven by a multipronged public health response. Seventh, several outcomes, such as opioid use and prescription, were indirectly measured using administrative and prescription claims data, which may not fully capture actual medication consumption or patient-level clinical outcomes. However, these measures are widely used in pharmacoepidemiological and health services research studies and provide valid and reproducible population-level indicators of prescribing behavior. Eighth, we did not systematically evaluate the robustness of our estimates using alternative ITS models (e.g., Prais-Winsten, restricted maximum likelihood, or autoregressive integrated moving average), because the combination of non-Gaussian outcomes and very short series in some included studies (i.e., only two to three pre- or postintervention time points) limited the feasibility of such sensitivity analyses. We acknowledge that some residual sensitivity to modeling assumptions may persist. However, our ITS analyses with the Newey-West heteroscedasticity- and autocorrelation-consistent standard errors are expected to provide reasonably robust estimates in this context. Ninth, because the majority of studies included in the meta-analyses were conducted in the United States and Canada, these findings may not be fully generalizable to health care systems with different regulatory or cultural contexts. However, the concentration of research in these jurisdictions enabled robust pooled analyses of prescribing outcomes that may not have been feasible only with studies from elsewhere. The concentration of evidence in these settings likely reflects the disproportionate impact of the opioid crisis and the intensity of guideline implementation and evaluation efforts in North America. Finally, we excluded abstracts and articles in languages other than English or French due to limited resources. Though we were able to screen many abstracts of articles published in neither English nor French because these abstracts were often available in English, we excluded five studies at the full-text review stage. Given the small share of these studies among those excluded (5 out of 958), we believe that the risk of language bias in our study is likely minimal.

## Conclusion

Our study provides key evidence that opioid prescribing guidelines have reduced prescriptions not only for patients with CNCP but also for those with other pain conditions. These findings suggest that the guidelines have been broadly interpreted and applied beyond their intended scope. However, changes in opioid prescribing practices likely reflect the combined influence of multiple, overlapping policy, regulatory, and educational initiatives introduced over the past decade, as well as evolving clinical attitudes and public awareness of opioid-related harms. Thus, though our results underscore the important role of guidelines, they should be interpreted within the broader policy and health care context that collectively shaped prescribing behaviors.

Analysis of a breadth of outcome measures offers the most current evidence to enhance patient safety and improve targeted aspects of opioid prescribing practices. At the same time, further evaluation of the impact of guidelines on exempt populations and the development of tailored recommendations for these groups may be necessary to strengthen and sustain evidence-informed best practices in pain management.

## Supplementary Material

Supplementary Materials_February 2026.docx

## Data Availability

The data used to run meta-analyses are available upon request. The R code used in the current study is available in the GitHub repository (https://github.com/jihoon-jay).
