# Supplementary material for: The impact of opioid prescribing guidelines on opioid dispensation: A systematic review and meta-analysis
Source: Can J Pain. 2026 May 22;10(1):2650299. doi: 10.1080/24740527.2026.2650299 (PMC13203043; doi:10.1080/24740527.2026.2650299)
Supplement: Supplementary Materials_February 2026.docx [file UCJP_A_2650299_SM7261.docx]

**The impact of opioid prescribing guidelines on opioid dispensation: A systematic review and meta-analysis**

Jihoon Lim PhD^1^*, Parker Tope MScPH^2^*, Maximilian Schaefer MScPH^1^, Isaiah Williams MScPH^1^, Imen Farhat MSc^1^, Andrea Benedetti PhD^1,3^, Dimitra Panagiotoglou PhD^1†^

^1^ Department of Epidemiology, Biostatistics and Occupational Health, McGill University, Montréal, QC, Canada

^2^ Departments of Epidemiology, Harvard T.H. Chan School of Public Health, Boston, MA, USA

^3^ Research Institute of the McGill University Health Centre, Montréal, QC, Canada

* Equally contributing authors

^†^ Corresponding author

**Corresponding Author Contact Information:**

Dimitra Panagiotoglou, PhD

McGill University

Department of Epidemiology, Biostatistics, and Occupational Health

2001 McGill College Avenue, Suite 1200

Montreal, Quebec, H3A 1G1, Canada

Phone: +1 (514) 398-8451

Email: [dimitra.panagiotoglou@mcgill.ca](mailto:dimitra.panagiotoglou@mcgill.ca)

Appendix A: Registration Information

This project builds upon an initial iteration commissioned by Health Canada (January 2023), which was conducted under a restricted timeline. Due to the proprietary nature of the original work, this review was not registered, and a formal protocol was not developed prior to its commencement.

Appendix B: Systematic Review

Figure S1. Detailed PRISMA flowchart of study selection process including all five searches conducted


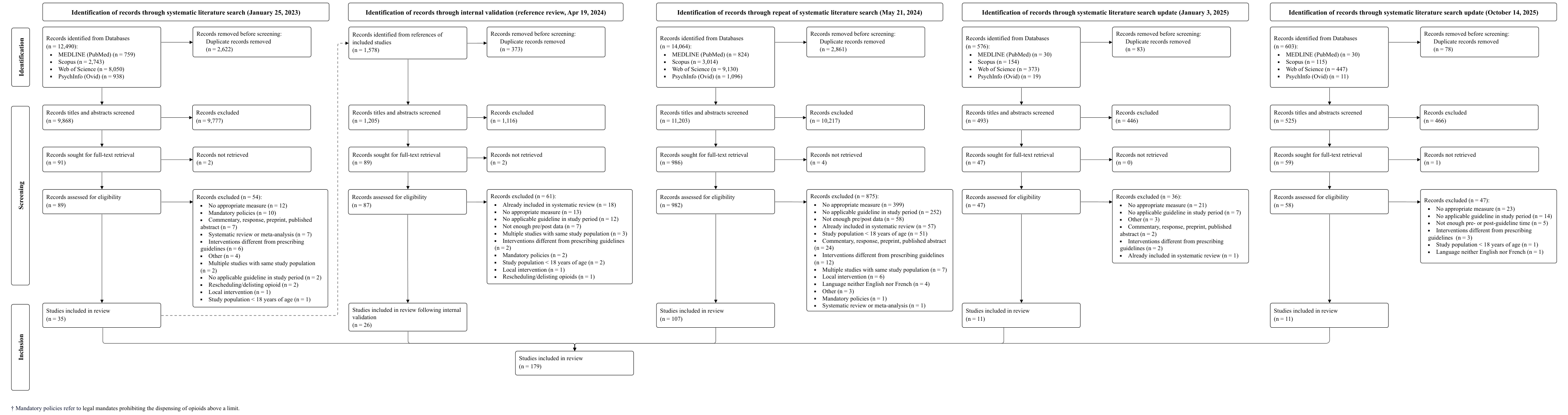


Table S1. Systematic search strategy

| **Database** | **Search Terms** |
| --- | --- |
| MEDLINE (PubMed) | ("analgesics, opioid"[MeSH Terms]) AND ("practice guidelines as topic"[MeSH Terms] OR "practice patterns, physicians’"[MeSH Terms] OR "drug prescriptions"[Mesh Terms]) AND ("chronic pain"[MeSH Terms]) |
| Scopus | (INDEXTERMS ("analgesics, opioid") OR INDEXTERMS ("narcotic analgesic agent")) AND (INDEXTERMS ("practice guidelines as topic") OR INDEXTERMS ("practice guideline") OR INDEXTERMS ("practice patterns, physicians'") OR INDEXTERMS ("drug prescriptions") OR INDEXTERMS ("prescription")) AND (INDEXTERMS ("chronic pain")) |
| Web of Science | (ALL=(analgesic*) OR ALL=(opioid*) OR ALL=(opiate*) OR ALL=(narcotic*)) AND (ALL=(physician* practice pattern*) OR ALL=(guideline*) OR ALL=(standard*) OR ALL=(prescri*)) AND (ALL=(chronic pain)) |
| PsycInfo (Ovid) | (exp analgesic drugs/ OR exp opiates/) AND (exp treatment guidelines/ OR exp drug therapy/ OR physician* practice patterns.mp. OR exp "prescribing (drugs)"/) AND (exp chronic pain/) |

Table S2. Characteristics of excluded opioid prescribing guidelines

| **Opioid prescribing guideline** | **Governing body** | **Abbreviation** | **Date of implementation/publication** | **Jurisdiction** | **Reason for exclusion** |
| --- | --- | --- | --- | --- | --- |
| ACOEM Guidelines for Chronic Use of Opioids^1^ | American College of Occupational and Environmental Medicine | ACOEM 2008 | 2008 | United States | Unable to retrieve |
| Guidelines for chronic use of opioids^2^ | American College of Occupational and Environmental Medicine | ACOEM 2011 | 2011 | United States | Unable to retrieve |
| American College of Occupational and Environmental Medicine practice guidelines: opioids for treatment of acute, subacute, chronic, and postoperative pain^3^ | American College of Occupational and Environmental Medicine | ACOEM 2014 | 2014-12-01 | United States | Includes recommendations for acute and sub-acute pain |
| Arizona opioid prescribing guidelines^4^ | Arizona Department of Health Services | ADHS 2014 | 2014-11-01 | Arizona, United States | Includes recommendations for acute pain |
| Therapeutic Guidelines: Analgesic^5^ | Analgesic Expert Group | AEG 2012 | 2012 | Australia | Unable to retrieve |
| The management of persistent pain in older persons^6^ | American Geriatrics Society Panel on Persistent Pain in Older Persons | AGS 2002 | 2002-06-01 | United States | Includes recommendations for cancer pain |
| Pharmacological management of persistent pain in older persons^7^ | American Geriatrics Society Panel on Pharmacological Management of Persistent Pain in Older Persons | AGS 2009 | 2009-07-29 | United States | No maximum dosage or duration recommendations |
| Principles regarding the use of opioid analgesics in patients with chronic noncancer pain^8^ | Australian and New Zealand College of Anaesthetists | ANZCA 2010 | 2010-08-01 | Australia & New Zealand | No mention of dosage or duration recommendations |
| The Use of Opioids for the Treatment of Chronic Pain^9^ | American Academy of Pain Medicine and the American Pain Society | APS 1997 | 1997-03-01 | United States | Policy paper |
| Practice guidelines for chronic pain management^10^ | American Society of Anesthesiologists Task Force on Pain Management, Chronic Pain Section | ASA 1997 | 1997-04-01 | United States | Does not include dosage or duration recommendations |
| Practice guidelines for chronic pain management: an updated report by the American Society of Anesthesiologists Task Force on Chronic Pain Management and the American Society of Regional Anesthesia and Pain Medicine^11^ | American Society of Anesthesiologists Task Force on Chronic Pain Management | ASA 2010 | 2010-04-01 | United States | No dosage or duration recommendations |
| Not reported^12^ | American Society of Interventional Pain Physicians | ASIPP 2000 | 2000 | United States | Unable to retrieve |
| American Society of Interventional Pain Physicians (ASIPP): Opioid Guidelines in the Management of Chronic Non-Cancer Pain^13^ | American Society of Interventional Pain Physicians | ASIPP 2006 | 2006-01-01 | United States | No dosage or duration recommendations |
| Opioid Guidelines in the Management of Chronic Non-Cancer Pain^14^ | American Society of Interventional Pain Physicians | ASIPP 2008 | 2008-03-01 | United States | No dosage or duration recommendations |
| American Society of Interventional Pain Physicians (ASIPP) guidelines for responsible opioid prescribing in chronic non-cancer pain^15^ | American Society of Interventional Pain Physicians | ASIPP 2012 | 2012-07-01 | United States | No dosage ceiling recommendations |
| Responsible, Safe, and Effective Prescription of Opioids for Chronic Non-Cancer Pain^16^ | American Society of Interventional Pain Physicians | ASIPP 2017 | 2017-02-01 | United States | No dosage ceiling recommendations |
| Pain in Residential Aged Care Facilities – Management Strategies^17^ | The Australian Pain Society | AusPS 2018 | 2018 | Australia | Unable to retrieve |
| Prescribing Controlled Substances for Pain^18^ | California Medical Board | California Medical Board 1994 | 1994 | California, United States | Unable to retrieve |
| Canadian Guidelines on Opioid Use Disorder Among Older Adults^19^ | Canadian Coalition of Seniors’ Mental Health | CCSMH 2020 | 2020-03-30 | Canada | Includes recommendations for acute pain |
| Clinical Practice Guideline for Prescribing Opioids for Pain^20^ | Center for Disease Control | CDC 2022 | 2022-11-04 | United States | Includes recommendations for acute pain |
| Policy for prescribing and dispensing opioids^21^ | Colorado Department of Regulatory Agencies | CDRA 2014 | 2014-07-01 | Colorado, United States | Unable to retrieve |
| Policy for prescribing and dispensing opioids^22^ | Colorado Department of Regulatory Agencies | CDRA 2018 | 2018-03-16 | Colorado, United States | Unable to retrieve |
| Policy for prescribing and dispensing opioids^23^ | Colorado Department of Regulatory Agencies | CDRA 2019 | 2019-03-14 | Colorado, United States | Includes recommendations for acute pain |
| Chronic Pain Disorder Medical Treatment Guidelines^24^ | Colorado Division of Workers' Compensation | Colorado DWC 2017 | 2017-11-30 | Colorado, United States | Does not differentiate between cancer pain and CNCP |
| Prescribing Guidelines for Pennsylvania. Opioid Dispensing Guidelines^25^ | Commonwealth of Pennsylvania & The Pennsylvania Pharmacists Association | COPPPA 2016 | 2016-01-14 | Pennsylvania, United States | Includes recommendations for acute pain |
| Use of opioid analgesics for the treatment of chronic noncancer pain^26^ | Canadian Pain Society | CPS 2002 | 2002-11-05 | Canada | Does not include dosage maximum or duration recommendations |
| Guidelines for managing chronic non-malignant pain. opioids and other agents^27^ | College of Physicians and Surgeons of Alberta | CPSA 1995 | 1995-01-01 | Alberta, Canada | Does not include dosage or duration recommendations |
| Kortlægning af opioidforbruget i Danmark. Med fokus på patienter med kroniske non-maligne smerter^28^ | Danish Health Authority | DHA 2016 | 2016-08-01 | Denmark | in Danish, unable to find English version |
| Aanpak van chronische pijn in de eerste lijn herziening 2017^29^ | EBM-Werkgroep Ontwikkeling Richtlijnen Eerste Lijn | EBM 2017 | 2017-02-01 | Netherlands | In Dutch, unable to find English version |
| European* clinical practice recommendations on opioids for chronic noncancer pain^30^ | European Pain Federation | EPF 2021 | 2021-03-02 | Unclear | Position paper |
| Establishing “best practices” for opioid rotation: conclusions of an expert panel^31^ | Beth Israel Medical Center & U Utah & Ad Hoc Expert Panel on Evidence Review and Guidelines for Opioid Rotation | Expert Panel 2009 | 2009-09-23 | United States | Only talks about opioid rotation |
| Reconsidering opioid therapy^32^ | Hunter Integrated Pain Service | HIPS 2013 | 2013 | New South Wales, Australia | Unable to retrieve |
| Aðgerðir til að sporna við misnotkun lyfja sem geta valdið ávana og fíkn [Means to reduce the abuse of medications that can cause dependence and addiction]^33^ | Velferðarráðuneytið / Ministry of Health | IMH 2018 | 2018-05-01 | Iceland | In Icelandic, unable to find English version |
| Guidelines for prescribing opioid analgesics for chronic noncancer pain^34^ | Committee for the Guidelines for Prescribing Opioid Analgesics for Chronic Noncancer Pain of JSPC | JSPC 2012 | 2012 | Japan | Unable to retrieve |
| Guidelines for pharmacologic management of neuropathic pain^35^ | The Japanese Society of Pain Clinicians | JSPC 2016 | 2016 | Japan | Not specific to chronic non-cancer pain |
| Recommendations for using opioids in chronic non-cancer pain^36^ | Kalso et al | Kalso 2003 | 2003 | Unclear | Jurisdiction unclear, does not include dosage or duration recommendations |
| Latin American Pain Federation position paper on appropriate opioid use in pain management^37^ | Latin American Pain Federation | LAPF 2019 | 2019-05-21 | Unclear | Position paper |
| Guidelines for prescribing controlled substances for pain^38^ | Medical Board of California | MBC 2014 | 2014-11-01 | California, United States | Includes recommendations for acute pain |
| Guidelines for prescribing controlled substances for pain^39^ | Medical Board of California | MBC 2023 | 2023-07-01 | California, United States | Includes recommendations for acute pain |
| Opioids in chronic non-cancer pain, situation and guidelines in Nordic countries^40^ | National Agency for Medicines | NAM 1999 | 1999 | Nordic countries | Unable to retrieve |
| National Guidelines: The use of opioids in the treatment of long-lasting non-malignant pain^41^ | Norwegian Directorate of Health | NDH 2014 | 2014 | Norway | Unable to retrieve |
| Nasjonal veileder for bruk av opioider ved langvarige ikke-kreftrelaterte smerter^42^ | Norwegian Directorate of Health | NDH 2015 | 2015 | Norway | Unable to retrieve |
| Nasjonal faglig veileder for bruk av opioider – ved langvarige ikke-kreftrelaterte smerter [National recommendation for opioid treatment for chronic non-cancer pain]^43^ | Norwegian Directorate of Health | NDH 2016 | 2016-09-14 | Norway | Unable to retrieve |
| Addictive Drugs, 8. Opioids^44^ | The Norwegian Directorate of Health | NDH 2021 | 2021-10-19 | Norway | In Norwegian, unable to find English version |
| NHG-standaard Pijn^45^ | NHG-Werkgroep Pijn | NHG 2015 | 2015-09-01 | Netherlands | In Dutch, unable to find English version |
| Pain^46^ | Nederlands Huisartsen Genootschap | NHG 2018 | 2018-06-01 | Netherlands | In Dutch, unable to find English version |
| Opioid deprescribing for persistent non-cancer pain^47^ | NHS | NHS 2020 | 2020-04-13 | United Kingdom | Only includes tapering recommendatons |
| Opioid deprescribing for persistent non-cancer pain^48^ | NHS | NHS 2023 | 2023-04-01 | United Kingdom | Only includes tapering recommendatons |
| Chronic pain (primary and secondary) in over 16s: assessment of all chronic pain and management of chronic primary pain (NG-193)^49^ | National Institute for Health and Care Excellence | NICE 2021 | 2021-04-07 | United Kingdom | Does not include dosage recommendations |
| Treatment recommendation: Use of opioids in chronic non-malignant pain—an update^50^ | Norwegian Medicines Agency | NMA 2008 | 2008 | Norway | Unable to retrieve |
| Pennsylvania Prescription Drug Monitoring Program (PDMP) System User and Stakeholder Training. Opioid Prescribing Guide^51^ | Pennsylvania Department of Health & Pennsylvania Department of Drug and Alcohol Problems | PDOH 2017 | 2017-09-29 | Pennsylvania, United States | Includes recommendations for acute pain |
| Prescribing drugs of dependence in general practice, Part C2: The role of opioids in pain management^52^ | The Royal Australian College of General Practitioners | RACGP 2017 | 2017-10-01 | Australia | Includes recommendations for acute pain |
| Recommendations for the use of opioids in Brazil^53^ | Brazilian Society for the Study of Pain | SBED 2013 | 2013-10-01 | Brazil | Includes recommendations for acute pain |
| Joint revised pain management guidelines | South Carolina Boards of Medical Examiners, Dentistry, and Nursing | SCBMEDN 2014 | 2014-11-01 | South Carolina, United States | Includes recommendations for acute pain |
| National guidelines for the safe prescribing of opioids 2021^54^ | Ministry of Health (Singapore) | SMH 2021 | 2021-04-01 | Singapore | Includes recommendations for acute pain |
| Användning av opioider vid långvarig icke-cancerrelaterad smärta – Rekommendationer 2002^55^ | Swedish Medical Products Agency | SMPA 2002 | 2002 | Sweden | Unable to retrieve |
| Läkemedel vid långvarig smärta hos barn och vuxna – behandlings­rekommendation [Pharmacological treatment of long-term pain in children and adults]^56^ | Swedish Medical Products Agency | SMPA 2017 | 2017 | Sweden | In Swedish, unable to find English version |
| Förskrivning av opioider i Sverige. Läkemedel, doser och diagnoser [Prescribed opioids in Sweden. Drugs, doses and diagnoses]^57^ | Läkemedelsverket / Swedish Medical Products Agency | SMPA 2020 | 2020-02-17 | Sweden | In Swedish, unable to find English version |
| Guidelines on Clinical Use of Pethidine^58^ | Taiwan Food and Drug Administration | TFDA 2011 | 2011-09-01 | Taiwan | Unable to retrieve |
| Guidelines on Clinical Use of Pethidine^59^ | Taiwan Food and Drug Administration | TFDA 2017 | 2017-11-07 | Taiwan | Unable to retrieve |
| Guidelines on Clinical Use of Pethidine^60^ | Taiwan Food and Drug Administration | TFDA 2022 | 2022-11-03 | Taiwan | In Chinese, unable to find English version |
| Utah clinical guidelines on prescribing opioids for treatment of pain^61^ | Utah Department of Health | UDH 2009 | 2009-03-01 | Utah, United States | Includes recommendations for acute pain |
| Managing Chronic Non-Terminal Pain in Adults, Including Prescribing Controlled Substances^62^ | University of Michigan Health System | UMHS 2012 | 2012-03-01 | Michigan, United States | Does not explicitly exclude cancer pain |
| Not reported^63^ | US Department of Veterans Affairs and US Department of Defense | VA/DoD 2003 | 2003 | United States/ Veterans | Unable to retrieve |
| Clinical Practice Guideline for Management of Opioid Therapy for Chronic Pain^64^ | US Department of Veterans Affairs and US Department of Defense | VA/DoD 2010 | 2010 | United States/ Veterans | Includes recommendations for cancer pain |
| Opioid therapy for chronic pain: overview of the 2017 US Department of Veterans Affairs and US Department of Defense clinical practice guideline^65^ | US Department of Veterans Affairs and US Department of Defense | VA/DoD 2017 | 2017-09-01 | United States/ Veterans | Includes recommendations for acute pain |
| 2022 Updated U.S. Department of Veterans Affairs and U.S. Department of Defense Clinical Practice Guideline^66^ | US Department of Veterans Affairs and US Department of Defense | VA/DoD 2022 | 2022-05-01 | United States | Includes recommendations for acute pain |
| Quick clinical guideline for the use of opioids in chronic non-malignant pain^67^ | Western Australia Drug and Alcohol Office | WADAO 2009 | 2009 | Western Australia, Australia | Unable to retrieve |
| Guideline for prescribing opioids to treat pain in injured workers^68^ | Washington State Department of Labor and Industries | WDLI 2013 | 2013-07-01 | Washington, United States | Includes recommendations for acute pain |
| Pain (chronic) (part of "Official Disability Guidelines")^69^ | Work Loss Data Institute | WLDI 2011 | 2011 | Unclear | Unable to retrieve |
| Statement on the use of opioids for the treatment of chronic pain^70^ | State Medical Society of Wisconsin | WMJ 2001 | 2001 | Wisconsin, United States | Unable to retrieve |
| Guidelines for the assessment and management of chronic pain.^71^ | Wisconsin Medical Society Task Force on Pain Management | WMS 2004 | 2004 | Wisconsin, United States | Unable to retrieve |
| Not reported^72^ | Washington State | WS 2011 | 2011-07-01 | Washington, United States | Enforceable law |
| Not reported^72^ | Washington State | WS 2012 | 2012-01-01 | Washington, United States | Enforceable law |

Table S3. Quality Appraisal Checklist for Case Series Studies (original document from the Institute of Health Economics, adapted for the current systematic review)

**LEGEND**

| **Study Subset** | **Colour** |
| --- | --- |
| **All studies** |  |
| **GUIDELINE-SPECIFIC STUDIES (i.e., ITS, pre-post studies)** |  |
| **NON-GUIDELINE-SPECIFIC STUDIES (i.e., descriptive studies)** |  |

| **STUDY OBJECTIVE** | | |
| --- | --- | --- |
| **GUIDELINE-SPECIFIC STUDIES (i.e., ITS, pre-post studies)** | | |
| **1A** | | **Was the hypothesis/aim/objective of the study clearly stated?**  **Yes**: The hypothesis/aim/objective of the study was clearly reported (includes population (either general or pain type specific), guideline, and outcome).  **Partial**: Only one or two components (population, guideline, or outcome) were included.  **No**: The hypothesis/aim/objective was not reported. |
| **NON-GUIDELINE-SPECIFIC STUDIES (i.e., descriptive studies)** | | |
| **1B** | | **Was the aim/objective of the study clearly stated?**  **Yes**: The aim/objective of the study was clearly reported (includes population (either general or pain type specific), and outcome).  **Partial**: Only one component (population or outcome) was included.  **No**: The aim/objective was not reported. |
| **STUDY DESIGN** | | |
| **GUIDELINE-SPECIFIC STUDIES (i.e., ITS, pre-post studies)** | | |
| **2** | | **If there were sufficient data for conduct of ITS analysis, was ITS analysis conducted?**  **Yes:** It was clearly stated that ITS was possible and conducted to ascertain impact of guideline on outcome of interest.  **Unclear**: Unclear or no information was provided.  **No:** It was not clearly stated if ITS was possible, and ITS was not conducted. |
| **STUDY POPULATION** | | |
| **ALL STUDIES** | | |
| **3** | | **Were the characteristics of the patients included in the study described and reported (e.g., Table 1)?**  **Yes**: All of the most relevant characteristics of the patients were reported (for example, number, age, gender, ethnicity, severity of disease/condition, comorbidity, or etiology).  **Partial**: Some, but not all, of the most relevant characteristics were reported.  **No**: Only the number of patients was reported. |
| **4** | | **Were study populations clearly defined (e.g., if specific or non-specific to a pain type)?**  **Yes**: If study population was the general population, inclusion and exclusion criteria were clearly defined. If study population was specific to a pain type, the pain-type (diagnosis and duration) inclusion and exclusion criteria were clearly defined.  **Partial**: Either the inclusion or exclusion criteria were reported. For pain type specific populations, only one of either diagnosis of pain type or related condition, or duration were clearly defined.  **No**: Neither inclusion nor exclusion criteria were reported. |
| **5** | | **If data source was a survey, was sampling error accounted for in weighting?**  **Yes**: Sampling error details associated with the survey were mentioned in the methods, and sampling error was accounted for through weighting.  **Unclear**: Sampling error details associated with the survey were mentioned in the methods, but sampling error was not accounted for through weighting.  **No**: Sampling error details nor weighting were mentioned. |
| **INTERVENTION AND CO-INTERVENTION** | | |
| **GUIDELINE-SPECIFIC STUDIES (i.e., ITS, pre-post studies)** | | |
| **6** | | **Was the guideline of interest clearly described?**  **Yes**: All of the most relevant characteristics of the guideline were reported (e.g., specific recommendations, the target population of the guideline, date of implementation, geographic jurisdiction, accessible citation to guideline included in references).  **Partial**: Some, but not all, of the most relevant characteristics were reported.  **No**: Only the name of the guideline was reported. |
| **OUTCOME MEASURES** | | |
| **ALL STUDIES** | | |
| **7** | | **Were relevant outcome measures established a priori?**  **Yes**: All relevant outcome measures (i.e., numerator and denominator (if applicable) were stated in the introduction or methods section.  **Partial**: Some, but not all, of the relevant outcome measures were stated in the introduction or method section (e.g., outcome measure reported, but numerator and denominator were not explicitly defined).  **No**: None of the relevant outcome measures were stated in the introduction or method section. |
| **8** | | **Were the relevant outcomes measured using appropriate objective/subjective methods?**  **Yes**: All relevant outcomes were measured with appropriate methods. These measures can be objective (e.g., gold standard tests or standardized clinical tests), subjective (e.g., self-administered questionnaires, standardized forms, or patient symptoms interview forms), or both. In the case of administrative data sources, measurements of outcomes were appropriately operationalized.  **Partial**: Some, but not all, relevant outcomes were measured with appropriate methods.  **No**: The methods used to measure the relevant outcomes were inappropriate. |
| **STATISTICAL ANALYSIS** | | |
| **GUIDELINE-SPECIFIC STUDIES (i.e., ITS, pre-post studies)** | | |
| **9** | **Were the model specifications for ITS analysis, if conducted, appropriately reported?**  **Yes**: The model terms or equation were explicitly stated, and tests for as well as accounting for serial correlation were explicitly stated.  **Unclear**: Some, not all elements listed above were stated in the methods section of the study.  **No**: No design or model specifications for conduct of ITS analysis were reported. | |
| **RESULTS AND CONCLUSIONS** | | |
| **GUIDELINE-SPECIFIC STUDIES (i.e., ITS, pre-post studies)** | | |
| **10** | | **Was there sufficient data (at least 8 measures pre- and post-guideline) for an evaluation of impact of guideline on outcome of interest?**  **Yes**: At least 8 measures pre- and post-guideline were included in time series.  **Unclear**: The length of follow-up was not clearly reported.  **No**: Less than 8 measures pre- and post-guideline were included in time series. |
| **ALL STUDIES** | | |
| **11** | | **Did the study provided estimates of random variability (measurements of uncertainty) in the data analysis of relevant outcomes?**  **Yes**: The estimates of the random variability (for example, standard error, standard deviation, confidence interval for normally distributed data or range and interquartile range for non-normally distributed data) were reported for all of the relevant outcomes or could be calculated from the raw data presented in the study.  **Partial**: The estimates of the random variability were reported for some, but not all of the relevant outcomes.  **No**: The estimates of the random variability were not reported for any of the relevant outcomes. |
| **12** | | **If data were visualized, did the study use appropriate methods for data visualization?**  **Yes**: If an ITS or pre-post study, time series were visualized appropriately. For descriptive studies, time series were visualized appropriately (e.g., scatter plots or line plots of outcome over time were reported with axes clearly labelled).  **Partial**: If an ITS or pre-post study, time series were visualized, but some aspects (e.g., axis labels, clarity of range of time series) were missing. For descriptive studies, time series were visualized, but some aspects (e.g., axis labels, clarity of range of time series) were missing, or inappropriate methods were used for data visualization (e.g., bar plots were used to visualize time series data).  **No**: Time series data of outcomes of interest were not visualized. |
| **13** | | **Were the conclusions of the study supported by the results?**  **Yes**: The conclusions of the study were supported by the evidence presented in the results and discussion sections.  **Unclear**: Unclear conclusion statement that makes it difficult to link the presented evidence to conclusions.  **No**: The conclusions were not supported by the evidence presented in the results and discussion sections. |
| **COMPETING INTERESTS AND SOURCES OF SUPPORT** | | |
| **14** | | **Were both competing interests and sources of support for the study reported?**  **Yes**: Both competing interests and sources of support (financial or other) received for the study were reported; or the absence of any competing interest and source of support was acknowledged.  **Partial**: Either the competing interest or source of support was reported.  **No**: Neither competing interests nor sources of support were reported. |

Table S4. Characteristics of studies included in the qualitative systematic review

| First author (year) | Location | Period of observation | Design | Guideline | Pain type |
| --- | --- | --- | --- | --- | --- |
| Abbott et al. (2018) | USA | 2006-2010 | Retrospective cohort | APS-AAPM, 2009 | General |
| Ackerman et al. (2018) | Australia | 2006-2016 | Ecological | ANZCA, 2015 | General |
| Adalbert et al. (2022) | Pennsylvania, USA | 2016-2020 | Ecological | COP, 2018 | General |
| Adams et al. (2019) | USA | 2008-2014 | Retrospective cohort | APS-AAPM, 2009 | General |
| Adewumi et al. (2021) | Queensland, Australia | 1997-2018 | Retrospective cohort | ANZCA, 2015 | General |
| Agarwal et al. (2020) | USA | 2013-2017 | Repeated cross-sectional | CDC, 2016 | General, Cancer |
| Ahn et al. (2025) | South Korea | 2010-2022 | Retrospective cohort | KPS, 2017 | Chronic |
| Ali et al. (2019) | USA | 2005-2015 | Retrospective cohort | APS-AAPM, 2009 | General |
| Aronowitz et al. (2021) | Pennsylvania, USA | 2013-2017 | Retrospective cohort | CDC, 2016 | General |
| Atluri et al. (2014) | USA | 2004-2011 | Ecological | APS-AAPM, 2009 | General |
| Aubry et al. (2022) | USA | 2010-2019 | Ecological | CDC, 2016 | General |
| Axeen et al. (2018)^a^ | USA | 1996-2012 | Repeated cross-sectional | APS-AAPM, 2009 | CNCP |
| Axeen et al. (2018)^b^ | USA | 2006-2012 | Retrospective cohort | APS-AAPM, 2009 | General |
| Bandara et al. (2022) | USA | 2012-2019 | Retrospective cohort | CDC, 2016 | CNCP, Cancer |
| Bao et al. (2021) | USA | 2011-2017 | Retrospective cohort | CDC, 2016 | CNCP, Cancer |
| Basham et al. (2024) | USA | 2005-2021 | Retrospective cohort | CDC, 2016 | General |
| Beaugard et al. (2022) | USA | 2015-2018 | Retrospective cohort | CDC, 2016 | General |
| Becker et al. (2016) | USA | 1999-2010 | Retrospective cohort | APS-AAPM, 2009 | General |
| Bedson et al. (2016) | UK | 2002-2012 | Retrospective cohort | BPS, 2010 | General |
| Bhargava et al. (2022) | USA | 2011-2018 | Retrospective cohort | CDC, 2016 | Acute |
| Bhattacharya et al. (2025) | USA | 2012-2020 | Retrospective cohort | CDC, 2016 | General |
| Black-Tiong et al. (2021) | Australia | 2012-2018 | Retrospective cohort | ANZCA, 2015 | CNCP |
| Bohnert et al. (2018) | USA | 2012-2017 | Interrupted time series | CDC, 2016 | General |
| Brat et al. (2018) | USA | 2008-2014 | Retrospective cohort | APS-AAPM, 2009 | General |
| Brett et al. (2025) | Australia, New Zealand, USA, Canada, UK, Denmark, South Korea | 2000-2020 | Retrospective cohort | ANZCA, 2015; APS, 2009; CDC, 2016; Health Canada, 2017; BPS, 2010; DHA, 2018; KPS, 2017 | General, CNCP |
| Bruun et al. (2024) | Denmark | 2005-2022 | Retrospective cohort | DHA, 2017 | CNCP |
| Camacho et al. (2024) | Australia | 2014-2021 | Retrospective cohort | ANZCA, 2015 | General |
| Canizares et al. (2023) | Ontario, Canada | 2004-2017 | Retrospective cohort | NOUGG, 2010 | Acute |
| Casagrande et al. (2023) | USA | 2008-2019 | Retrospective cohort | CDC, 2016 | General |
| Chai et al. (2018) | USA | 1997-2015 | Retrospective cohort | APS-AAPM, 2009 | General |
| Champagne-Langabeer et al. (2021) | USA | 2015-2017 | Ecological | CDC, 2016 | General |
| Chen et al. (2019) | USA | 2003-2014 | Retrospective cohort | APS-AAPM, 2009 | CNCP |
| Chen et al. (2022) | USA | 2013-2018 | Retrospective cohort | CDC, 2016 | Cancer |
| Chhibba et al. (2021) | USA | 2006-2017 | Repeated cross-sectional | APS-AAPM, 2009; CDC, 2016 | Chronic |
| Choi et al. (2022) | USA | 2015-2020 | Ecological | CDC, 2016 | General |
| Choudhry (2024) | USA | 2014-2019 | Retrospective cohort | CDC, 2016 | General |
| Cohen et al. (2022) | USA | 2000-2019 | Retrospective cohort | APS-AAPM, 2009 | CNCP |
| Crabtree et al. (2019) | British Columbia, Canada | 2015-2017 | Interrupted time series | CPSBC, 2016 | General |
| Curtis et al. (2017) | USA | 2007-2014 | Retrospective cohort | APS-AAPM, 2009 | Chronic |
| Curtis et al. (2019) | UK | 1998-2017 | Ecological | BPS, 2010 | General |
| Dart et al. (2015) | USA | 2002-2013 | Ecological | APS-AAPM, 2009 | General |
| Daubresse et al. (2013) | USA | 2000-2010 | Repeated cross-sectional | APS-AAPM, 2009 | CNCP |
| Daubresse et al. (2019) | USA | 2007-2014 | Retrospective cohort | APS-AAPM, 2009 | Chronic |
| Davies et al. (2019) | Wales | 2005-2015 | Repeated cross-sectional | BPS, 2010 | General |
| Day et al. (2023) | USA | 2010-2020 | Retrospective cohort | CDC, 2016 | Acute |
| Dayer et al. (2019) | USA | 2015-2017 | Retrospective cohort | CDC, 2016 | Acute |
| Denis et al. (2019) | Pennsylvania, USA | 2007-2017 | Retrospective cohort | APS-AAPM, 2009 | CNCP |
| DiPrete et al. (2024) | USA | 2010-2018 | Retrospective cohort | CDC, 2016 | General |
| Dryden et al. (2022) | USA | 2013-2017 | Retrospective cohort | CDC, 2016 | General |
| Esechie et al. (2021) | USA | 2013-2018 | Retrospective cohort | CDC, 2016 | General |
| Fenton et al. (2019) | USA | 2008-2017 | Retrospective cohort | CDC, 2016 | CNCP |
| Fischer et al. (2018) | Ontario, Canada | 2005-2016 | Retrospective cohort | NOUGG, 2010 | General |
| Foy et al. (2016) | UK | 2005-2012 | Retrospective cohort | BPS, 2010 | General |
| Franklin et al. (2012) | USA | 1996-2010 | Ecological | WA, 2007 | General |
| Fulton-Kehoe et al. (2015) | Washington, USA | 2006-2010 | Retrospective cohort | WA, 2007 | CNCP |
| Garcia et al. (2019)^a^ | Massachusetts, USA | 2002-2017 | Retrospective cohort | APS-AAPM, 2009 | General |
| Garcia et al. (2019)^b^ | USA | 2014-2017 | Ecological | CDC, 2016 | General |
| Garg et al. (2013) | Washington, USA | 2004-2010 | Retrospective cohort | WA, 2007 | Acute |
| Gibson et al. (2021) | USA | 2008-2016 | Retrospective cohort | APS-AAPM, 2009 | General |
| Gillies et al. (2024) | NSW, Australia | 2014-2020 | Retrospective cohort | ANZCA, 2015 | Chronic |
| Gleber et al. (2020) | USA | 2012-2018 | Retrospective cohort | CDC, 2016 | Acute |
| Goldstick et al. (2021) | USA | 2011-2017 | Retrospective cohort | CDC, 2016 | CNCP |
| Gomes et al. (2014) | Canada | 2006-2011 | Ecological | NOUGG, 2010 | General |
| Gomes et al. (2017) | Canada | 2008-2016 | Retrospective cohort | NOUGG, 2010 | General |
| Gorfinkel et al. (2022) | USA | 2014-2018 | Repeated cross-sectional | CDC, 2016 | General |
| Goudman et al. (2024) | USA | 2012-2022 | Retrospective cohort | CDC, 2016 | Chronic |
| Gupta et al. (2020) | USA | 2015-2017 | Interrupted time series | CDC, 2016 | General |
| Guy et al. (2017) | USA | 2006-2015 | Retrospective cohort | APS-AAPM, 2009 | General |
| Hales et al. (2020) | USA | 2009-2018 | Ecological | CDC, 2016 | General |
| Han et al. (2020) | USA | 2006-2015 | Retrospective cohort | APS-AAPM, 2009 | Chronic |
| Hauser et al. (2017) | USA, Canada, Germany | 1980-2014 | Ecological | APS-AAPM, 2009; NOUGG, 2010; GPS, 2009 | CNCP |
| Hebert et al. (2022) | UK | 2005-2020 | Interrupted time series | SIGN, 2013 | General |
| Hechter et al. (2023) | California, USA | 2013-2020 | Repeated cross-sectional | CDC, 2016 | General |
| Hedenmalm et al. (2019) | Germany | 2006-2016 | Retrospective cohort | GPS, 2009 | General |
| Hirschtritt et al. (2018) | USA | 1993-2014 | Repeated cross-sectional | APS-AAPM, 2009 | General |
| Holmer et al. (2021) | USA | 2012-2020 | Retrospective cohort | CDC, 2016 | Chronic |
| Hong et al. (2022) | USA | 2015-2019 | Repeated cross-sectional | CDC, 2016 | General |
| Hu et al. (2025) | USA | 2011-2021 | Ecological | CDC, 2016 | General |
| Imtiaz et al. (2020) | USA, Canada | 2001-2015 | Ecological | APS-AAPM, 2009; NOUGG, 2010 | General |
| Iyengar et al. (2024) | USA | 2013-2017 | Retrospective cohort | CDC, 2016 | Chronic |
| Jairam et al. (2021) | USA | 2013-2017 | Retrospective cohort | CDC, 2016 | General, Cancer, Palliative |
| Jami et al. (2021) | USA | 2011-2017 | Retrospective cohort | CDC, 2016 | CNCP |
| Janakiram et al. (2023) | USA | 2012-2019 | Retrospective cohort | CDC, 2016 | Chronic, Acute |
| Jin et al. (2022) | USA | 2014-2018 | Retrospective cohort | CDC, 2016 | Acute |
| Jones et al. (2021) | Canada | 2005-2020 | Retrospective cohort | Health Canada, 2017 | General |
| Kang et al. (2024) | USA | 2011-2019 | Interrupted time series | CDC, 2016 | CNCP |
| Karst et al. (2019) | Tennessee, USA | 2015-2018 | Retrospective cohort | CDC, 2016 | Acute |
| Kazanis et al. (2018) | USA | 2006-2013 | Retrospective cohort | APS-AAPM, 2009 | General |
| Kern et al. (2020) | USA | 2002-2018 | Retrospective cohort | APS-AAPM, 2009; CDC, 2016 | General |
| Khouja et al. (2021) | USA | 2015-2019 | Retrospective cohort | CDC, 2016 | General |
| Khouja et al. (2022) | USA | 2011-2017 | Retrospective cohort | CDC, 2016 | General |
| Kimmel et al. (2017) | USA | 2006-2010 | Retrospective cohort | APS-AAPM, 2009 | Chronic |
| Kimmel et al. (2024) | USA | 2011-2020 | Retrospective cohort | CDC, 2016 | Chronic |
| Kirubalingam et al. (2022) | Ontario, Canada | 2013-2019 | Retrospective cohort | Health Canada, 2017 | Acute |
| Kluger et al. (2022) | USA | 2009-2017 | Retrospective cohort | CDC, 2016 | General |
| Kuo et al. (2016) | USA | 2007-2012 | Retrospective cohort | APS-AAPM, 2009 | General |
| Kuo et al. (2024) | USA | 2005-2018 | Retrospective cohort | APS-AAPM, 2009; CDC, 2016 | CNCP |
| Kurdi et al. (2021) | Scotland | 2010-2019 | Repeated cross-sectional | SIGN, 2013 | General |
| Labaran et al. (2020) | USA | 2008-2015 | Retrospective cohort | APS-AAPM, 2009 | Acute |
| Ladapo et al. (2018) | USA | 2005-2015 | Repeated cross-sectional | APS-AAPM, 2009 | General |
| Lalic et al. (2018) | Australia | 2013-2017 | Retrospective cohort | ANZCA, 2015 | CNCP, Cancer |
| Larochelle et al. (2015) | USA | 2001-2010 | Repeated cross-sectional | APS-AAPM, 2009 | Acute |
| Lazar et al. (2021) | New York, USA | 2014-2019 | Retrospective cohort | CDC, 2016 | Acute |
| LeBrett et al. (2021) | USA | 2006-2016 | Repeated cross-sectional | APS-AAPM, 2009 | Acute |
| Lee et al. (2019) | USA | 2002-2015 | Retrospective cohort | APS-AAPM, 2009 | Chronic |
| Leja et al. (2022) | Michigan, USA | 2015-2018 | Retrospective cohort | CDC, 2016 | Chronic |
| Levy et al. (2015) | USA | 2007-2012 | Retrospective cohort | APS-AAPM, 2009 | General |
| Lin et al. (2023) | USA | 2010-2017 | Retrospective cohort | CDC, 2016 | CNCP |
| Liu et al. (2023) | USA | 2013-2021 | Retrospective cohort | CDC, 2016 | General |
| Liu et al. (2024) | USA | 2011-2018 | Retrospective cohort | CDC, 2016 | Acute |
| Losina et al. (2023) | USA | 2003-2016 | Repeated cross-sectional | APS-AAPM, 2009 | Chronic |
| Ly et al. (2025) | Quebec, Canada | 1997-2018 | Retrospective cohort | CMQ, 2009 | General |
| Maharjan et al. (2023) | USA | 2013-2018 | Retrospective cohort | CDC, 2016 | General |
| Maierhofer et al. (2023) | USA | 2012-2018 | Interrupted time series | CDC, 2016 | CNCP, Acute |
| Marrie et al. (2023) | Manitoba, Canada | 1998-2017 | Retrospective cohort | NOUGG, 2010 | CNCP |
| Mauck et al. (2024) | USA | 2001-2020 | Retrospective cohort | APS-AAPM, 2009 | Acute |
| May et al. (2024) | British Columbia, Canada | 2008-2018 | Retrospective cohort | NOUGG, 2010 | Chronic |
| Mazurenko et al. (2021) | USA | 2014-2018 | Interrupted time series | CDC, 2016 | CNCP |
| Meadows et al. (2020) | USA | 2011-2017 | Ecological | CDC, 2016 | General |
| Mehta et al. (2021)^a^ | USA | 2011-2017 | Retrospective cohort | CDC, 2016 | General |
| Mehta et al. (2021)^b^ | USA | 2011-2017 | Retrospective cohort | CDC, 2016 | CNCP, Acute |
| Moawad et al. (2021) | USA | 2012-2018 | Retrospective cohort | CDC, 2016 | Acute |
| Mojtabai et al. (2018) | USA | 1999-2014 | Repeated cross-sectional | APS-AAPM, 2009 | General |
| Molnar et al. (2022) | Canada | 2012-2018 | Retrospective cohort | Health Canada, 2017 | Chronic |
| Morden et al. (2014) | USA | 2007-2011 | Retrospective cohort | APS-AAPM, 2009 | General |
| Nahin et al. (2019) | USA | 1997-2014 | Repeated cross-sectional | APS-AAPM, 2009 | General |
| Nelson et al. (2019) | USA | 2007-2013 | Retrospective cohort | APS-AAPM, 2009 | Cancer |
| Neprash et al. (2021) | USA | 2011-2017 | Retrospective cohort | CDC, 2016 | CNCP |
| Nobel et al. (2019) | USA | 2014-2018 | Retrospective cohort | CDC, 2016 | Acute |
| Noh et al. (2022) | South Korea | 2012-2018 | Repeated cross-sectional | KPS, 2017 | CNCP |
| Oh et al. (2024) | South Korea | 2016-2020 | Retrospective cohort | KPS, 2017 | Chronic |
| Oliva et al. (2022) | Spain | 2016-2019 | Ecological | S-MS, 2017 | General |
| Oliva et al. (2023) | Spain | 2016-2020 | Ecological | S-MS, 2017 | General |
| Paik et al. (2021) | USA | 2013-2017 | Retrospective cohort | CDC, 2016 | Chronic |
| Panagiotoglou et al. (2025) | British Columbia, Canada | 2012-2020 | Interrupted time series | CPSBC, 2016 | CNCP, Cancer, Palliative |
| Peckham et al. (2019) | USA | 2006-2016 | Repeated cross-sectional | APS-AAPM, 2009 | General |
| Pensa et al. (2018) | USA | 2003-2013 | Repeated cross-sectional | APS-AAPM, 2009 | General |
| Piper et al. (2018) | USA | 2006-2016 | Ecological | APS-AAPM, 2009 | General |
| Premkumar et al. (2022) | USA | 2013-2017 | Retrospective cohort | CDC, 2016 | Chronic |
| Pritchard et al. (2022) | USA | 2011-2019 | Repeated cross-sectional | CDC, 2016 | CNCP, Acute |
| Rancu et al. (2025)^a^ | USA | 2011-2021 | Retrospective cohort | CDC, 2016 | Acute |
| Rancu et al. (2025)^b^ | USA | 2011-2021 | Retrospective cohort | CDC, 2016 | Acute |
| Rhee et al. (2018) | USA | 2006-2015 | Repeated cross-sectional | APS-AAPM, 2009 | General |
| Richter et al. (2017) | Minnesota, USA | 2005-2015 | Retrospective cohort | APS-AAPM, 2009 | Chronic |
| Rodin et al. (2025) | USA | 2010-2020 | Interrupted time series | CDC, 2016 | Cancer |
| Rogal et al. (2019) | USA | 2005-2014 | Retrospective cohort | APS-AAPM, 2009 | Chronic |
| Rolova et al. (2025) | Denmark | 2010-2023 | Repeated cross-sectional | DHA, 2018 | General |
| Romman et al. (2020) | USA | 2013-2017 | Ecological | CDC, 2016 | General |
| Roy et al. (2024) | USA | 2014-2019 | Retrospective cohort | CDC, 2016 | CNCP |
| Rui et al. (2020) | USA | 2006-2017 | Repeated cross-sectional | APS-AAPM, 2009 | Acute |
| Salas et al. (2021) | USA | 2014-2017 | Interrupted time series | CDC, 2016 | CNCP |
| Salvatore et al. (2022) | USA | 2015-2019 | Interrupted time series | CDC, 2016 | General |
| Scherrer et al. (2020) | USA | 2010-2018 | Retrospective cohort | CDC, 2016 | General |
| Schieber et al. (2019) | USA | 2006-2017 | Retrospective cohort | APS-AAPM, 2009 | General |
| Schieber et al. (2020) | USA | 2008-2018 | Ecological | APS-AAPM, 2009 | General |
| Scott et al. (2022) | North Staffordshire, UK | 2000-2015 | Repeated cross-sectional | BPS, 2010 | Chronic |
| Sears et al. (2020) | Washington, USA | 2012-2017 | Retrospective cohort | CDC, 2016 | General |
| Sears et al. (2021) | Washington, USA | 2008-2015 | Interrupted time series | WA, 2010 | General |
| Shen et al. (2018) | USA | 2006-2013 | Repeated cross-sectional | APS-AAPM, 2009 | CNCP |
| Sites et al. (2014) | USA | 2000-2010 | Repeated cross-sectional | APS-AAPM, 2009 | General |
| Smith et al. (2020) | Illinois, USA | 2009-2018 | Ecological | CDC, 2016 | General |
| Smolina et al. (2016) | British Columbia, Canada | 2005-2013 | Retrospective cohort | NOUGG, 2010 | General |
| Song et al. (2022) | South Korea | 2010-2019 | Retrospective cohort | KPS, 2017 | CNCP |
| Stokes et al. (2019) | USA | 1999-2016 | Repeated cross-sectional | APS-AAPM, 2009 | CNCP |
| Strong et al. (2024) | USA | 2015-2017 | Retrospective cohort | CDC, 2016 | CNCP |
| Suda et al. (2022) | USA | 2015-2018 | Ecological | CDC, 2016 | General |
| Sullivan et al. (2016) | Washington, USA | 2006-2010 | Ecological | WA, 2007 | General |
| Surbhi et al. (2025) | USA | 2004-2018 | Retrospective cohort | APS, 2009; CDC, 2016 | CNCP |
| Sutherland et al. (2021) | USA | 2013-2018 | Interrupted time series | CDC, 2016 | Acute |
| Taqi et al. (2023) | UK | 2000-2014 | Retrospective cohort | BPS, 2010 | General |
| Tehrani et al. (2018) | USA | 2005-2015 | Ecological | APS-AAPM, 2009 | General |
| Thiels et al. (2019) | USA | 2009-2018 | Retrospective cohort | CDC, 2016 | Acute |
| Thompson et al. (2018) | USA | 2004-2014 | Retrospective cohort | APS-AAPM, 2009 | Acute |
| Thompson et al. (2024) | USA | 2010-2019 | Repeated cross-sectional | CDC, 2016 | General |
| Togun et al. (2021) | USA | 2014-2018 | Interrupted time series | CDC, 2016 | CNCP |
| Torrance et al. (2018) | Scotland | 2003-2012 | Ecological | BPS, 2010 | General |
| Townsend et al. (2021) | USA | 2014-2018 | Interrupted time series | CDC, 2016 | CNCP |
| Tucker et al. (2021) | USA | 2014-2017 | Retrospective cohort | CDC, 2016 | CNCP |
| Turcotte et al. (2024) | USA | 2015-2022 | Retrospective cohort | CDC, 2016 | Chronic |
| Wei et al. (2022) | USA | 2011-2018 | Retrospective cohort | CDC, 2016 | CNCP |
| Weiner et al. (2022) | Oregon, USA | 2015-2017 | Retrospective cohort | CDC, 2016 | General |
| Wembridge et al. (2024) | Australia | 2010-2020 | Retrospective cohort | ANZCA, 2015 | Acute |
| Whitney et al. (2023) | USA | 2011-2017 | Retrospective cohort | CDC, 2016 | Chronic |
| Woods et al. (2021) | USA | 2009-2018 | Ecological | APS-AAPM, 2009; CDC, 2016 | General |
| Xie et al. (2022) | Spain | 2007-2019 | Retrospective cohort | DPS, 2018 | General |
| Zamora-Legoff et al. (2016) | Minnesota, USA | 2005-2014 | Retrospective cohort | APS-AAPM, 2009 | Chronic |
| Zaveri et al. (2020) | USA | 2014-2018 | Retrospective cohort | CDC, 2016 | Acute |
| Zeng et al. (2021) | UK | 2000-2016 | Retrospective cohort | BPS, 2010 | CNCP |
| Zhu et al. (2019) | USA | 2012-2017 | Retrospective cohort | CDC, 2016 | General |
| Zin et al. (2020) | Singapore | 2005-2014 | Repeated cross-sectional | PAS, 2013 | General |

Abbreviations: ANZCA = Australian and New Zealand College of Anaesthetists; APS-AAPM = American Pain Society-American Academy of Pain Medicine; BPS = British Pain Society; CDC = Centers for Disease Control and Prevention; CMQ = Collège des médecins du Québec; CNCP = Chronic non-cancer pain; COP = Commonwealth of Pennsylvania; DHA = Danish Health Authority; GPS = German Pain Society; KPS = Korean Pain Society; NOUGG = National Opioid Use Guideline Group; SIGN = Scottish Intercollegiate Guideline Network; S-MS = Socidrogalcohol-Ministerio de Sanidad ; UK = United Kingdom; USA = United States of America; WA = Washington state

Axeen et al. (2018)^a^: Axeen, S., Seabury, S.A. & Menchine, M. Emergency Department Contribution to the Prescription Opioid Epidemic. Ann Emerg Med 71, 659-667 e653 (2018).

Axeen et al. (2018)^b^: Axeen S., Trends in Opioid Use and Prescribing in Medicare, 2006-2012. Health Serv Res. 2018;53(5):3309-2810.1111/1475-6773.12846.

Garcia et al. (2019)^a^: Garcia, M. M., et al. (2019). "Impact of Sequential Opioid Dose Reduction Interventions in a State Medicaid Program Between 2002 and 2017." J Pain **20**(8): 876-884.

Garcia et al. (2019)^b^: Garcia, M. C., et al. (2019). "Opioid Prescribing Rates in Nonmetropolitan and Metropolitan Counties Among Primary Care Providers Using an Electronic Health Record System - United States, 2014-2017." MMWR Morb Mortal Wkly Rep **68**(2): 25-30.

Mehta et al. (2021)^a^: Mehta, H.B., et al. Time Trends in Opioid Use by Dementia Severity in Long-Term Care Nursing Home Residents. J Am Med Dir Assoc 22, 124-131.e121 (2021).

Mehta et al. (2021)^b^: Mehta, H.B., et al. State Variation in Chronic Opioid Use in Long-Term Care Nursing Home Residents. J Am Med Dir Assoc **22**(12): 2593-2599 e2594.

Rancu et al. (2025)^a^: Rancu AL, Gouzoulis MJ, Winter AD, Katsnelson BM, Ansah-Twum JK, Grauer JN. Opioid Prescribing Trends Following Lumbar Discectomy. J Am Acad Orthop Surg. 2024 Dec 19;33(18):1054-1059. doi: 10.5435/JAAOS-D-24-00908. PMID: 39706160.

Rancu et al. (2025)^b^: Rancu, Albert & Salib, Andrew & Kammien, Alexander & Lizardi, Juan & Allam, Omar & Grauer, Jonathan & Alperovich, Michael. (2024). Opioid Use Following Open Reduction and Internal Fixation of Mandibular Fractures. The Journal of craniofacial surgery. 36. 10.1097/SCS.0000000000010930.

Table S5. Quality assessment of studies included in the systematic review

| **Study** | **Type** | **1A** | **1B** | **2A** | **2B** | **3** | **4** | **5** | **6** | **7** | **8** | **9** | **10** | **11** | **12** | **13** | **14** | **15** |
| --- | --- | --- | --- | --- | --- | --- | --- | --- | --- | --- | --- | --- | --- | --- | --- | --- | --- | --- |
| Abbott et al. 2018 | NGS | NA | Yes | NA | NA | Yes | Yes | NA | NA | Yes | Yes | NA | NA | No | NA | Unclear | Yes | No |
| Ackerman et al. 2018 | NGS | NA | Yes | NA | NA | No | No | NA | NA | Partial | Yes | NA | NA | No | Yes | Yes | Yes | Yes |
| Adalbert et al. 2022 | NGS | NA | Yes | NA | NA | No | No | NA | NA | Yes | Yes | NA | NA | No | Yes | Yes | Yes | Yes |
| Adams et al. 2019 | NGS | NA | Yes | NA | NA | Yes | Yes | NA | NA | Yes | Yes | NA | NA | No | Yes | Yes | Partial | Yes |
| Adewumi et al. 2021 | NGS | NA | Yes | NA | NA | No | Yes | NA | NA | Yes | Yes | NA | NA | No | NA | Yes | Yes | Yes |
| Agarwal et al. 2020 | NGS | NA | Yes | NA | NA | Yes | Yes | NA | NA | Yes | Yes | NA | NA | Yes | Yes | Yes | Yes | Yes |
| Ahn et al. 2025 | NGS | NA | Yes | NA | NA | Yes | Yes | NA | NA | Yes | Yes | NA | NA | Yes | Yes | Yes | Yes | Yes |
| Ali et al. 2019 | NGS | NA | Partial | NA | NA | No | Yes | NA | NA | Yes | Yes | NA | NA | No | Yes | Unclear | Partial | No |
| Aronowitz et al. 2021 | NGS | NA | Yes | NA | NA | Yes | Yes | NA | NA | Yes | Yes | NA | NA | No | Yes | Yes | Yes | No |
| Atluri et al. 2014 | NGS | NA | Partial | NA | NA | No | Yes | NA | NA | Yes | Yes | NA | NA | No | NA | Unclear | Yes | No |
| Aubry et al. 2022 | NGS | NA | No | NA | NA | No | No | NA | NA | Yes | Yes | NA | NA | No | Yes | Unclear | Yes | No |
| Axeen et al. 2018a | NGS | NA | Yes | NA | NA | Yes | Yes | Yes | NA | Yes | Yes | NA | NA | No | Yes | Yes | Yes | Yes |
| Axeen et al. 2018b | NGS | NA | Partial | NA | NA | Yes | Yes | NA | NA | Yes | Yes | NA | NA | No | Yes | Yes | Yes | Yes |
| Bandara et al. 2022 | NGS | NA | Yes | NA | No | Yes | Yes | NA | NA | Yes | Yes | NA | NA | Yes | Yes | Yes | Yes | Yes |
| Bao et al. 2021 | NGS | NA | No | NA | NA | No | Partial | NA | NA | Yes | Yes | NA | NA | No | Yes | Yes | Yes | No |
| Basham et al. 2024 | NGS | NA | Yes | NA | NA | Yes | Partial | NA | NA | Yes | Yes | NA | NA | No | Yes | Yes | Yes | Yes |
| Battacharya et al. 2025 | NGS | NA | Yes | NA | NA | Yes | Yes | NA | NA | Yes | Yes | NA | NA | Yes | Yes | Partial | Yes | Yes |
| Beaugard et al. 2022 | NGS | NA | Yes | NA | NA | Yes | Yes | NA | NA | Yes | Yes | NA | NA | Yes | Yes | Yes | Yes | Yes |
| Becker et al. 2016 | NGS | NA | Yes | NA | NA | Yes | Yes | NA | NA | Yes | Yes | NA | NA | No | Yes | Yes | Partial | No |
| Bedson et al. 2016 | NGS | NA | Yes | NA | NA | No | Yes | NA | NA | Yes | Yes | NA | NA | No | Yes | Unclear | Yes | No |
| Bhargava et al. 2022 | GS | Yes | NA | Yes | No | Yes | Yes | NA | Yes | Yes | Yes | NA | Yes | Yes | Yes | Yes | Yes | Yes |
| Black-Tiong et al. 2021 | NGS | NA | Yes | NA | NA | Yes | Yes | NA | NA | Yes | Yes | NA | NA | No | Yes | Yes | Yes | Yes |
| Bohnert et al. 2018 | GS | Yes | NA | Yes | Yes | No | Yes | NA | Yes | Yes | Yes | Yes | Yes | Yes | Yes | Yes | Yes | Yes |
| Brat et al. 2018 | NGS | NA | Yes | NA | NA | Yes | Yes | NA | NA | Yes | Yes | NA | NA | No | Yes | Yes | Yes | Yes |
| Brett et al. 2025 | NGS | NA | Yes | NA | NA | Yes | Yes | NA | NA | Yes | Yes | NA | NA | Yes | Yes | Yes | Yes | Yes |
| Bruun et al. 2024 | NGS | NA | Yes | NA | NA | Yes | Yes | NA | NA | Yes | Yes | NA | NA | No | Yes | Yes | Yes | Yes |
| Camacho et al. 2024 | NGS | NA | Partial | NA | NA | Yes | Yes | NA | NA | Yes | Yes | NA | NA | No | Yes | Unclear | Yes | Yes |
| Canizares et al. 2023 | NGS | NA | Yes | NA | NA | Yes | Partial | NA | NA | Yes | Yes | NA | NA | No | Yes | Yes | Yes | Yes |
| Casagrande et al. 2023 | NGS | NA | Yes | NA | NA | Yes | Yes | NA | NA | Yes | Yes | NA | NA | No | Yes | Yes | Yes | No |
| Chai et al. 2018 | NGS | NA | Yes | NA | NA | No | Yes | NA | NA | Yes | Yes | NA | NA | No | Yes | Yes | Yes | No |
| Champagne-Langabeer et al. 2021 | NGS | NA | Yes | NA | NA | No | Yes | NA | NA | Yes | Yes | NA | NA | No | Partial | Yes | No | No |
| Chen et al. 2019 | NGS | NA | Yes | NA | NA | Yes | Yes | NA | NA | Yes | Yes | NA | NA | No | Yes | Yes | Yes | Yes |
| Chen et al. 2022 | NGS | NA | Yes | NA | NA | Yes | Yes | NA | Yes | Yes | Yes | No | Yes | No | Yes | Yes | Yes | Yes |
| Chhibba et al. 2021 | NGS | NA | Yes | NA | NA | Yes | Yes | Yes | NA | Yes | Yes | NA | NA | Yes | Yes | Yes | Partial | No |
| Choi et al. 2022 | NGS | NA | No | NA | NA | Yes | No | NA | NA | Yes | Yes | NA | NA | No | Yes | Yes | Yes | Yes |
| Choudhry 2024 | NGS | NA | Yes | NA | NA | Yes | Yes | NA | NA | Yes | Yes | NA | NA | Yes | NA | Yes | Yes | Yes |
| Cohen et al. 2022 | NGS | NA | Yes | NA | NA | Yes | No | NA | NA | Yes | Yes | NA | NA | No | Yes | Yes | Yes | Yes |
| Crabtree et al. 2019 | GS | Yes | NA | Yes | Yes | No | Yes | NA | Yes | Yes | Yes | Partial | Yes | No | Yes | Yes | Partial | Yes |
| Curtis et al. 2017 | NGS | NA | Yes | NA | NA | Yes | Partial | NA | NA | Yes | Yes | NA | NA | No | Yes | Yes | Partial | Yes |
| Curtis et al. 2019 | NGS | NA | Yes | NA | NA | Partial | Yes | NA | NA | Yes | Yes | NA | NA | No | Yes | Yes | Yes | Yes |
| Dart et al. 2015 | NGS | NA | Yes | NA | NA | Yes | Partial | NA | NA | Yes | Yes | NA | NA | No | Yes | Yes | Yes | Yes |
| Daubresse et al. 2013 | NGS | NA | Yes | NA | NA | Yes | Yes | Yes | NA | Yes | Yes | NA | NA | No | Yes | Yes | Partial | No |
| Daubresse et al. 2019 | NGS | NA | Yes | NA | NA | Yes | Yes | NA | NA | Yes | Yes | NA | NA | Partial | Yes | Yes | Yes | No |
| Davies et al. 2019 | NGS | NA | Yes | NA | NA | Yes | Yes | NA | NA | Yes | Yes | NA | NA | No | Yes | Yes | Yes | Yes |
| Day et al. 2023 | NGS | NA | Yes | NA | NA | Partial | Yes | NA | NA | Yes | Yes | NA | NA | No | Yes | Yes | Partial | Yes |
| Dayer 2019 | GS | Yes | NA | Yes | No | Yes | Yes | NA | Yes | Partial | Yes | NA | NA | No | NA | Partial | No | Yes |
| Denis et al. 2019 | NGS | NA | Yes | NA | NA | Yes | Yes | NA | NA | Yes | Yes | NA | NA | No | Partial | Yes | Yes | Yes |
| DiPrete et al. 2024 | NGS | NA | Partial | NA | NA | Yes | Yes | NA | NA | Yes | Yes | NA | NA | No | NA | Yes | Yes | Yes |
| Dryden et al. 2022 | NGS | NA | Yes | NA | NA | Partial | Partial | NA | NA | Yes | Yes | NA | NA | No | NA | Unclear | Partial | Yes |
| Esechie et al. 2021 | NGS | NA | Yes | NA | NA | No | No | NA | NA | Yes | Yes | NA | NA | No | Yes | Yes | Yes | Yes |
| Fenton et al. 2019 | GS | Yes | NA | Yes | No | Yes | Yes | NA | Yes | Yes | Yes | NA | Yes | Yes | Yes | Yes | Yes | Yes |
| Fischer et al. 2018 | NGS | NA | Yes | NA | NA | No | No | NA | NA | Yes | Yes | NA | NA | No | Yes | Yes | Yes | Yes |
| Foy et al. 2016 | NGS | NA | Yes | NA | NA | Yes | Yes | NA | NA | Yes | Yes | NA | NA | No | NA | Yes | Yes | Yes |
| Franklin et al. 2012 | GS | Yes | NA | Yes | No | No | Yes | NA | Yes | Yes | Yes | NA | Yes | Yes | Yes | Yes | Partial | Yes |
| Fulton-Kehoe et al. 2015 | GS | Yes | NA | Yes | No | Yes | Yes | NA | Yes | Yes | Yes | NA | NA | No | Yes | Yes | Partial | Yes |
| Garcia et al. 2019a | NGS | NA | Yes | NA | NA | Partial | Yes | NA | NA | Yes | Yes | NA | NA | No | Yes | Yes | Yes | Yes |
| Garcia et al. 2019b | NGS | NA | Yes | NA | NA | No | Yes | NA | NA | Yes | Yes | NA | NA | No | Yes | Yes | Partial | No |
| Garg et al. 2013 | GS | Yes | NA | Yes | No | No | Yes | NA | Yes | Yes | Yes | NA | Yes | Partial | Yes | Yes | Yes | Yes |
| Gibson et al. 2021 | NGS | NA | Yes | NA | NA | Yes | Yes | NA | NA | Yes | Yes | NA | NA | No | Yes | Yes | Yes | Yes |
| Gillies et al. 2024 | NGS | NA | Yes | NA | NA | Yes | Yes | NA | NA | Yes | Yes | NA | NA | No | Partial | Yes | Yes | Yes |
| Gleber et al. 2020 | NGS | NA | Yes | NA | NA | No | Yes | NA | NA | Yes | Yes | NA | NA | No | Yes | Yes | Partial | No |
| Goldstick et al. 2021 | GS | Yes | NA | Yes | No | Yes | Yes | NA | Yes | Yes | Yes | NA | NA | Yes | Yes | Yes | Yes | Yes |
| Gomes et al. 2014 | NGS | NA | Partial | NA | NA | No | Yes | NA | NA | Yes | Yes | NA | NA | No | Yes | Yes | Yes | No |
| Gomes et al. 2017 | NGS | NA | Partial | NA | NA | No | Yes | NA | NA | Yes | Yes | NA | NA | No | Yes | Yes | Yes | No |
| Gorfinkel et al. 2022 | NGS | NA | Yes | NA | NA | Yes | Yes | Yes | NA | Yes | Yes | NA | NA | Yes | NA | Yes | Yes | Yes |
| Goudman et al. 2024 | NGS | NA | Yes | NA | NA | Partial | Partial | NA | NA | Yes | Yes | NA | NA | No | Yes | Yes | Yes | Yes |
| Gupta et al. 2020 | GS | Partial | NA | Yes | Yes | No | Yes | NA | Yes | Partial | Yes | No | NA | Yes | Yes | Yes | Partial | Yes |
| Guy et al. 2017 | NGS | NA | Partial | NA | NA | Yes | No | NA | NA | Yes | Yes | NA | NA | No | Yes | Unclear | Partial | No |
| Hales et al. 2020 | NGS | NA | Yes | NA | NA | Partial | Yes | Yes | NA | Yes | Yes | NA | NA | No | Yes | Yes | Partial | No |
| Han et al. 2020 | NGS | NA | Yes | NA | NA | Yes | Yes | NA | NA | Yes | Yes | NA | NA | No | Yes | Yes | Yes | Yes |
| Hauser et al. 2017 | NGS | NA | Yes | NA | NA | No | No | NA | NA | Yes | Yes | NA | NA | No | Yes | Yes | Partial | No |
| Hébert et al. 2022 | GS | Yes | NA | Yes | Yes | Yes | Yes | NA | Yes | Yes | Yes | Yes | NA | Yes | Yes | Yes | Yes | Yes |
| Hechter et al. 2023 | NGS | NA | Yes | NA | NA | Partial | Yes | NA | NA | Yes | Yes | NA | NA | No | Yes | Yes | Yes | Yes |
| Hedenmalm et al. 2019 | NGS | NA | Yes | NA | NA | Yes | No | NA | NA | Yes | Yes | NA | NA | No | Yes | Yes | Partial | No |
| Hirschtritt et al. 2018 | NGS | NA | Yes | NA | NA | No | Yes | Yes | NA | Yes | Yes | NA | NA | No | Yes | Yes | Yes | Yes |
| Holmer et al. 2021 | NGS | NA | Yes | NA | NA | Yes | Yes | NA | NA | Yes | Yes | NA | NA | No | Yes | Yes | Yes | Yes |
| Hong et al. 2022 | NGS | NA | Yes | NA | NA | Yes | Yes | Yes | NA | Yes | Yes | NA | NA | No | Yes | Yes | Partial | Yes |
| Hu et al. 2025 | NGS | NA | Yes | NA | NA | Yes | Yes | No | NA | Yes | Yes | NA | NA | Yes | Yes | Yes | Yes | No |
| Imtiaz et al. 2020 | NGS | NA | Partial | NA | NA | No | No | NA | NA | Yes | Yes | NA | NA | No | Yes | Unclear | Yes | Yes |
| Iyengar et al. 2024 | NGS | NA | Yes | NA | NA | Yes | Yes | NA | NA | Yes | Yes | NA | NA | No | Yes | Yes | Yes | Yes |
| Jairam et al. 2021 | NGS | NA | Yes | NA | NA | Yes | Yes | NA | NA | Partial | Yes | NA | NA | No | Yes | Yes | Yes | Yes |
| Jami et al. 2021 | NGS | NA | Yes | NA | NA | Yes | Yes | NA | NA | Yes | Yes | NA | NA | No | Yes | Yes | Yes | Yes |
| Janakiram et al. 2023 | NGS | NA | Yes | NA | NA | Yes | Yes | NA | NA | Yes | Yes | NA | NA | No | Yes | Yes | Yes | Yes |
| Jin et al. 2022 | NGS | NA | Yes | NA | NA | Yes | Yes | NA | NA | Yes | Yes | NA | NA | Yes | Yes | Yes | Yes | Yes |
| Jones et al. 2021 | NGS | NA | No | NA | NA | No | No | NA | NA | Yes | Yes | NA | NA | No | Partial | Yes | Yes | Yes |
| Kang et al. 2024 | GS | Yes | NA | Yes | Yes | Yes | Yes | NA | Yes | Yes | Yes | Yes | Yes | No | Yes | Yes | Yes | Yes |
| Karst et al. 2019 | NGS | NA | Yes | NA | NA | Yes | Yes | NA | NA | Yes | Yes | NA | NA | No | Partial | Yes | Partial | Yes |
| Kazanis et al. 2018 | NGS | NA | Yes | NA | NA | No | No | NA | NA | Yes | Yes | NA | NA | No | Yes | Yes | Partial | Yes |
| Kern et al. 2020 | NGS | NA | Yes | NA | NA | No | Yes | NA | NA | Yes | Yes | NA | NA | No | Yes | Yes | Yes | Yes |
| Khouja et al. 2021 | NGS | NA | Yes | NA | NA | No | Yes | NA | NA | Yes | Yes | NA | NA | No | Partial | Yes | Yes | No |
| Khouja et al. 2022 | NGS | NA | Yes | NA | NA | Yes | Yes | NA | NA | Yes | Yes | NA | NA | No | NA | Unclear | Yes | Yes |
| Kimmel et al. 2017 | NGS | NA | Yes | NA | NA | Partial | Yes | NA | NA | Yes | Yes | NA | NA | No | NA | Unclear | Yes | No |
| Kimmel et al. 2024 | NGS | NA | Partial | NA | NA | Yes | Partial | NA | NA | Yes | Yes | NA | NA | No | Yes | Yes | Yes | Yes |
| Kirubalingam et al. 2022 | NGS | NA | Yes | NA | NA | Yes | Yes | NA | NA | Yes | Yes | NA | NA | Yes | Yes | Yes | Yes | Yes |
| Kluger et al. 2022 | NGS | NA | Yes | NA | NA | Yes | Yes | NA | NA | Yes | Yes | NA | NA | No | NA | Unclear | Yes | Yes |
| Kuo et al. 2016 | NGS | NA | No | NA | NA | Yes | Yes | NA | NA | Yes | Yes | NA | NA | No | Yes | Yes | Yes | No |
| Kuo et al. 2024 | NGS | NA | Yes | NA | NA | Partial | Yes | NA | NA | Yes | Yes | NA | NA | No | Yes | Yes | Yes | Yes |
| Kurdi et al. 2021 | NGS | NA | Partial | NA | NA | No | Yes | NA | NA | Yes | Yes | NA | NA | No | Yes | Unclear | Yes | Yes |
| Labaran et al. 2020 | NGS | NA | Yes | NA | NA | Yes | Yes | NA | NA | Yes | Yes | NA | NA | No | Yes | Yes | Partial | Yes |
| Ladapo et al. 2018 | NGS | NA | Yes | NA | NA | Yes | Partial | Yes | NA | Yes | Yes | NA | NA | No | Yes | Yes | Yes | Yes |
| Lalic et al. 2018 | NGS | NA | Yes | NA | NA | Yes | No | NA | NA | Yes | Yes | NA | NA | No | NA | Yes | Yes | Yes |
| Larochelle et al. 2015 | NGS | NA | Yes | NA | NA | Yes | Yes | Yes | NA | Yes | Yes | NA | NA | Yes | Yes | Yes | Yes | Yes |
| Lazar et al. 2021 | NGS | NA | Partial | NA | NA | Yes | Partial | NA | NA | Yes | Yes | NA | NA | No | NA | Yes | Yes | Yes |
| LeBrett et al. 2021 | NGS | NA | Yes | NA | NA | Yes | Yes | Yes | NA | Yes | Yes | NA | NA | No | Yes | Yes | Yes | Yes |
| Lee et al. 2019 | NGS | NA | Yes | NA | NA | Yes | Yes | NA | NA | Yes | Yes | NA | NA | Yes | Yes | Yes | Partial | Yes |
| Leja et al. 2022 | NGS | NA | Yes | NA | NA | Yes | Partial | NA | NA | Yes | Yes | NA | NA | No | Yes | Yes | Yes | Yes |
| Levy et al. 2015 | NGS | NA | Partial | NA | NA | No | No | NA | NA | Yes | Yes | NA | NA | No | NA | Unclear | Yes | No |
| Lin et al. 2023 | NGS | NA | Yes | NA | NA | Yes | Yes | NA | NA | Yes | Yes | NA | NA | No | Yes | Yes | Yes | Yes |
| Liu et al. 2023 | NGS | NA | Yes | NA | NA | Yes | Partial | NA | NA | Yes | Yes | NA | NA | No | Yes | Yes | Yes | Yes |
| Liu et al. 2024 | NGS | NA | Yes | NA | NA | Yes | Yes | NA | NA | Yes | Yes | NA | NA | No | Yes | Yes | Yes | Yes |
| Losina et al. 2023 | NGS | NA | Yes | NA | NA | Yes | Yes | Yes | NA | Yes | Yes | NA | NA | No | Yes | Yes | Yes | Yes |
| Ly et al. 2025 | NGS | NA | Yes | NA | NA | Yes | Yes | NA | NA | Yes | Yes | NA | NA | Yes | Yes | Yes | Yes | Yes |
| Maharjan et al. 2023 | NGS | NA | Yes | NA | NA | Yes | Yes | NA | NA | Yes | Yes | NA | NA | No | NA | Yes | Yes | Yes |
| Maierhofer et al. 2023 | NGS | Yes | NA | Yes | Yes | No | Partial | NA | Yes | Yes | Yes | NA | NA | No | Yes | Yes | Yes | Yes |
| Marrie et al. 2023 | NGS | NA | No | NA | NA | No | Yes | NA | NA | Yes | Yes | NA | NA | No | Yes | Unclear | Yes | Yes |
| Mauck et al. 2024 | NGS | NA | Yes | NA | NA | Yes | Yes | NA | NA | Yes | Yes | NA | NA | Yes | Yes | Unclear | Yes | Yes |
| May et al. 2024 | NGS | NA | Yes | NA | NA | Yes | Yes | NA | NA | Yes | Yes | NA | NA | No | Yes | Yes | Yes | Yes |
| Mazurenko et al. 2020 | GS | Yes | NA | NA | Yes | Yes | Yes | NA | Yes | Yes | Yes | Yes | Yes | Yes | NA | Yes | Yes | Yes |
| Meadows et al. 2020 | NGS | NA | Yes | NA | NA | No | No | NA | NA | Yes | Yes | NA | NA | No | Partial | Yes | Partial | Yes |
| Mehta et al. 2021a | NGS | NA | Yes | NA | NA | Yes | Yes | NA | NA | Yes | Yes | NA | NA | No | Yes | Yes | Yes | Yes |
| Mehta et al. 2021b | NGS | Partial | NA | NA | NA | Yes | Yes | NA | Partial | Yes | Yes | NA | NA | Partial | Yes | Yes | Yes | Yes |
| Moawad et al. 2021 | NGS | NA | Yes | NA | NA | Yes | Yes | NA | NA | Yes | Yes | NA | NA | Yes | Yes | Yes | Yes | Yes |
| Mojtabai et al. 2018 | NGS | NA | No | NA | NA | Yes | No | Yes | NA | Yes | Yes | NA | NA | No | Yes | Unclear | Yes | Yes |
| Molnar et al. 2022 | NGS | NA | Yes | NA | NA | Yes | Yes | NA | NA | Yes | Yes | NA | NA | Yes | Partial | Yes | Yes | Yes |
| Morden et al. 2014 | NGS | NA | Yes | NA | NA | Yes | Yes | NA | NA | Yes | Yes | NA | NA | No | NA | Yes | Partial | No |
| Nahin et al. 2019 | NGS | NA | Yes | NA | NA | Yes | No | Yes | NA | Yes | Yes | NA | NA | Yes | Yes | Yes | Yes | Yes |
| Nelson et al. 2019 | NGS | NA | Yes | NA | NA | Yes | Yes | NA | NA | Yes | Yes | NA | NA | No | No | Unclear | Yes | No |
| Neprash et al. 2021 | NGS | NA | Yes | NA | NA | Yes | Yes | NA | NA | Yes | Yes | NA | NA | Yes | Yes | Yes | Yes | Yes |
| Nobel et al. 2019 | NGS | NA | Yes | NA | NA | Yes | Yes | NA | NA | Yes | No | NA | NA | Partial | Partial | Yes | Yes | No |
| Noh et al. 2022 | NGS | NA | Yes | NA | NA | Yes | Yes | NA | NA | Yes | Yes | NA | NA | No | Yes | Yes | Yes | Yes |
| Oh et al. 2024 | NGS | NA | Yes | NA | NA | No | Yes | NA | NA | Yes | Yes | NA | NA | No | Yes | Yes | Yes | Yes |
| Oliva et al. 2022 | NGS | NA | Partial | NA | NA | No | No | NA | NA | Yes | Yes | NA | NA | No | Yes | Yes | Yes | Yes |
| Oliva et al. 2023 | NGS | NA | Partial | NA | NA | No | No | NA | NA | Yes | Yes | NA | NA | No | NA | Yes | Partial | No |
| Paik et al. 2022 | NGS | NA | Yes | No | No | Yes | Yes | NA | NA | Yes | Yes | NA | NA | No | Yes | Yes | Yes | Yes |
| Panagiotoglou et al. 2025 | GS | Yes | NA | Yes | Yes | No | Yes | NA | Yes | Yes | Yes | Partial | Yes | Yes | Yes | Yes | Yes | Yes |
| Peckham et al. 2019 | NGS | NA | Yes | NA | NA | Yes | Yes | Yes | NA | Yes | Yes | NA | NA | No | Yes | Unclear | Partial | No |
| Pensa et al. 2018 | NGS | NA | Yes | NA | NA | Yes | Yes | NA | NA | Yes | Yes | NA | NA | No | Yes | Yes | Yes | No |
| Piper et al. 2018 | NGS | NA | Yes | NA | NA | No | No | NA | NA | Yes | Yes | NA | NA | No | Yes | Yes | Partial | Yes |
| Premkumar et al. 2022 | NGS | NA | No | NA | NA | Yes | Yes | NA | NA | Yes | Yes | NA | NA | Yes | Yes | Yes | Yes | No |
| Pritchard et al. 2022 | GS | Yes | NA | No | No | Yes | Yes | Yes | Yes | Yes | Yes | NA | NA | Yes | Yes | Yes | Yes | Yes |
| Rancu et al. 2025a | NGS | NA | Yes | NA | NA | Yes | Yes | NA | NA | Yes | Yes | NA | NA | Yes | Yes | Yes | Partial | Yes |
| Rancu et al. 2025b | NGS | NA | Yes | NA | NA | Yes | Yes | NA | NA | Yes | Yes | NA | NA | Yes | Yes | Yes | Partial | Yes |
| Rhee et al. 2018 | NGS | NA | Yes | NA | NA | Yes | Partial | Yes | NA | Yes | Yes | NA | NA | No | Partial | Yes | Yes | Yes |
| Richter et al. 2017 | NGS | NA | Yes | NA | NA | Yes | Yes | NA | NA | Yes | Yes | NA | NA | No | Yes | Yes | Yes | Yes |
| Rodin R et al. 2025 | GS | Yes | NA | Yes | Yes | Yes | Yes | NA | Yes | Yes | Yes | Partial | Yes | Yes | Yes | Yes | Yes | Yes |
| Rogal et al. 2019 | NGS | NA | Yes | NA | NA | Yes | Yes | NA | NA | Yes | Yes | NA | NA | No | Yes | Yes | Yes | Yes |
| Rolova et al. 2025 | NGS | NA | Yes | NA | NA | No | Yes | NA | NA | Yes | Yes | NA | NA | Yes | Yes | Yes | Yes | No |
| Romman et al. 2020 | GS | Yes | NA | No | No | Yes | Yes | NA | Yes | Yes | Yes | NA | NA | No | Yes | Yes | Yes | Yes |
| Roy et al. 2024 | NGS | NA | Yes | NA | NA | Yes | Yes | NA | NA | Yes | Yes | NA | NA | No | Yes | Yes | Yes | Yes |
| Rui et al. 2020 | NGS | NA | Yes | NA | NA | Yes | Partial | Yes | NA | Yes | Yes | NA | NA | No | Yes | Yes | No | No |
| Salas et al. 2021 | GS | Yes | NA | Yes | Yes | Yes | Yes | NA | Yes | Yes | Yes | Partial | No | Yes | Yes | Yes | Yes | No |
| Salvatore et al. 2022 | GS | Yes | NA | Yes | Yes | Yes | Yes | NA | Yes | Yes | Yes | Yes | No | No | Partial | Yes | Yes | Yes |
| Scherrer et al. 2020 | NGS | NA | Yes | NA | NA | Yes | Yes | NA | NA | Yes | Yes | NA | NA | No | NA | Unclear | Yes | Yes |
| Schieber et al. 2019 | NGS | NA | Yes | NA | NA | Yes | Yes | Yes | NA | Yes | Yes | NA | NA | Yes | Yes | Yes | Yes | Yes |
| Schieber et al. 2020 | NGS | NA | Partial | NA | NA | Partial | Yes | NA | NA | Yes | Yes | NA | NA | Yes | Yes | Yes | Yes | Yes |
| Scott et al. 2022 | NGS | NA | Yes | NA | NA | No | Yes | NA | NA | Yes | Yes | NA | NA | Yes | Yes | Yes | Yes | Yes |
| Sears et al. 2020 | NGS | NA | Yes | NA | NA | No | No | NA | NA | Yes | Yes | NA | NA | No | Yes | Unclear | Yes | No |
| Sears et al. 2021 | GS | Yes | NA | Yes | Yes | Yes | Yes | NA | Yes | Yes | Yes | Yes | Yes | No | Yes | Yes | Partial | Yes |
| Shen et al. 2018 | NGS | NA | Yes | NA | NA | Yes | Yes | Yes | NA | Yes | Yes | NA | NA | No | NA | Unclear | Partial | No |
| Sites et al. 2014 | NGS | NA | Yes | NA | NA | Yes | Partial | Yes | NA | Yes | Yes | NA | NA | Yes | Yes | Yes | Yes | Yes |
| Smith et al. 2020 | NGS | NA | Yes | NA | NA | Yes | Yes | NA | NA | Yes | Yes | NA | NA | No | Yes | Yes | Yes | Yes |
| Smolina et al. 2016 | NGS | NA | Yes | NA | NA | Yes | Yes | NA | NA | Yes | Yes | NA | NA | No | Yes | Yes | Partial | Yes |
| Song et al. 2022 | NGS | NA | Yes | NA | NA | Yes | Yes | NA | NA | Yes | Yes | NA | NA | No | Yes | Yes | Yes | Yes |
| Stokes et al. 2019 | NGS | NA | Yes | NA | NA | Yes | Yes | Yes | NA | Yes | Yes | NA | NA | Yes | Yes | Yes | Yes | Yes |
| Strong et al. 2024 | NGS | NA | Yes | NA | NA | Yes | Yes | NA | NA | Yes | Yes | NA | NA | No | NA | Yes | Yes | Yes |
| Suda et al. 2022 | NGS | NA | Yes | NA | NA | Yes | No | NA | NA | Yes | Yes | NA | NA | No | NA | Yes | Yes | Yes |
| Sullivan et al. 2016 | GS | Yes | NA | Yes | No | No | Yes | NA | Yes | Yes | Yes | NA | NA | Yes | Partial | Yes | Yes | Yes |
| Surbhi et al. 2025 | NGS | NA | Yes | NA | NA | Yes | Yes | NA | NA | Yes | Yes | NA | NA | Yes | Yes | Yes | Yes | No |
| Sutherland et al. 2021 | GS | Yes | NA | Yes | Yes | Yes | Yes | NA | Yes | Yes | Yes | Partial | No | Yes | Yes | Yes | Yes | Yes |
| Taqi et al. 2023 | NGS | NA | No | NA | NA | Yes | Yes | NA | NA | Yes | Yes | NA | NA | No | Yes | Yes | Yes | No |
| Tehrani et al. 2018 | NGS | NA | Yes | NA | NA | No | Yes | NA | NA | Yes | Yes | NA | NA | No | Yes | Yes | Yes | No |
| Thiels et al. 2019 | NGS | NA | Yes | NA | NA | Yes | Yes | NA | NA | Yes | Yes | NA | NA | No | Yes | Unclear | Yes | Yes |
| Thompson et al. 2018 | NGS | NA | Yes | NA | NA | Partial | Partial | NA | NA | Yes | Yes | NA | NA | Yes | Yes | Yes | Yes | Yes |
| Thompson et al. 2024 | NGS | NA | Yes | NA | NA | Yes | Partial | Yes | NA | No | Yes | NA | NA | No | NA | Unclear | Yes | No |
| Togun et al. 2021 | GS | Yes | NA | Yes | Yes | Yes | Yes | NA | Yes | Yes | Yes | Yes | Yes | Yes | NA | Yes | Yes | Yes |
| Torrance et al. 2018 | NGS | NA | Partial | NA | NA | Yes | Partial | NA | NA | Yes | Yes | NA | NA | No | Yes | Yes | Yes | Yes |
| Townsend et al. 2022 | GS | Yes | NA | Yes | Yes | Yes | Yes | NA | Yes | Yes | Yes | Yes | No | Yes | Yes | Yes | Partial | No |
| Tucker et al. 2021 | GS | Yes | NA | Yes | No | Yes | Yes | NA | Yes | Yes | Yes | NA | NA | Partial | Yes | Yes | Partial | Yes |
| Turcotte et al. 2024 | NGS | NA | Yes | NA | NA | Yes | Yes | NA | NA | Yes | Yes | NA | NA | No | NA | Yes | Yes | Yes |
| Wei et al. 2022 | NGS | NA | No | NA | NA | Partial | Partial | NA | NA | Yes | Yes | NA | NA | Yes | Yes | Yes | Yes | Yes |
| Weiner et al. 2022 | NGS | NA | Yes | NA | NA | Yes | Yes | NA | NA | Yes | Yes | NA | NA | No | NA | Yes | Yes | Yes |
| Wembridge et al. 2024 | NGS | NA | Yes | NA | NA | Partial | Partial | NA | NA | Yes | Yes | NA | NA | No | Yes | Yes | No | No |
| Whitney et al. 2023 | NGS | NA | Yes | NA | NA | Yes | Yes | NA | NA | Yes | Yes | NA | NA | Yes | Yes | Yes | Yes | Yes |
| Woods et al. 2021 | NGS | NA | Yes | NA | NA | No | Yes | NA | NA | Yes | Yes | NA | NA | No | Partial | Yes | Yes | Yes |
| Xie et al. 2022 | NGS | NA | Partial | NA | NA | No | Partial | NA | NA | Yes | Yes | NA | NA | No | Yes | Yes | Yes | Yes |
| Zamora-Legoff et al. 2016 | NGS | NA | Yes | NA | NA | Yes | Partial | NA | NA | Yes | Yes | NA | NA | No | Yes | Yes | Yes | Yes |
| Zaveri et al. 2020 | NGS | NA | Yes | NA | NA | Yes | Yes | NA | NA | Yes | Yes | NA | NA | Partial | Partial | Unclear | Partial | Yes |
| Zeng et al. 2021 | NGS | NA | Yes | NA | NA | Yes | Yes | NA | NA | Yes | Yes | NA | NA | Yes | Yes | Yes | Yes | Yes |
| Zhu et al. 2019 | GS | Yes | NA | Yes | No | Yes | Yes | NA | Yes | Yes | Yes | NA | NA | No | Yes | Yes | Yes | No |
| Zin et al. 2020 | NGS | NA | Yes | NA | NA | No | Yes | NA | NA | Yes | Yes | NA | NA | No | Yes | Yes | Yes | No |

Abbreviations: GS = Guideline specific; NGS = Non-guideline specific

Questions:

1A: Was the hypothesis/aim/objective of the study clearly stated?

1B: Was the aim/objective of the study clearly stated?

2A: Was there sufficient data (at least 8 measures pre- and post-guideline) for an evaluation of impact of guideline on outcome of interest?

2B: Was ITS analysis conducted?

3: Were the characteristics of the patients included in the study described and reported (e.g., Table 1)?

4: Were study populations clearly defined (e.g., if specific or non-specific to a pain type)?

5: If data source was a survey, was sampling error accounted for in weighting?

6: Was the guideline of interest clearly described?

7: Were relevant outcome measures established a priori?

8: Were the relevant outcomes measured using appropriate objective/subjective methods?

9: Were the model specifications for ITS analysis, if conducted, appropriately reported?

10: Was there sufficient data (at least 8 measures pre- and post-guideline) for an evaluation of impact of guideline on outcome of interest?

11: Did the study provide estimates of random variability (measurements of uncertainty) in the data analysis of relevant outcomes?

12: Did the study use appropriate methods for data visualization?

13: Were the conclusions of the study supported by the results?

14: Were both competing interests and sources of support for the study reported?

15: Did the authors indicate whether they obtained ethics committee approval or stated an exemption from it?

Table S6. Summary of post-guideline trend changes from the qualitative systematic review

| **Outcome** | **Outcome Measure** | **Guideline** | **Post-intervention trend** | **First Author (Year)** |
| --- | --- | --- | --- | --- |
| **General Population** | | | | |
| Prevalence | Percentage of patients who had prescriptions for ≥ 90 days of an opioid, by year | APS-AAPM, 2009 | Positive | Abbott (2018) |
|  | Actual and projected number of total dispensed opioid prescriptions in Australia | ANZCA, 2015 | Positive | Ackerman (2018) |
|  | Total prescription | COP, 2018 | No change | Adalbert (2022) |
|  | Prevalence of post-deployment prescription opioid receipt | APS-AAPM, 2009 | Negative | Adams (2019) |
|  | Number of prescriptions | ANZCA 2015 | Negative | Adewumi (2021) |
|  | Opioid prescriptions per 100 Medicare beneficiaries | CDC, 2016 | No change | Agarwal (2020) |
|  | Number of users | APS-AAPM, 2009 | Positive | Ali (2019) |
|  | Percentage of beneficiaries who are prescribed opioids | APS-AAPM, 2009 | Negative | Axeen (2018)^1^ |
|  | Percentage of people with at least 1 tramadol dispensing | CDC, 2016 | Negative | Basham (2024) |
|  | Percentage of patients who received opioids | APS-AAPM, 2009 | Positive | Becker (2016) |
|  | Percentage of patients who filled opioids | APS-AAPM, 2009 | Positive | Brat (2018) |
|  | Opioid prescriptions per 1000 pregnancies | ANZCA, 2015 (Australia) | Positive | Brett (2025) |
|  |  | ANZCA, 2015 (New Zealand) | No change |  |
|  |  | APS-AAPM, 2009 (commercial) | Negative |  |
|  |  | APS-AAPM, 2009 (Medicaid) | Negative |  |
|  |  | CDC, 2016 (commercial) | No change |  |
|  |  | CDC, 2016 (Medicaid) | No change |  |
|  |  | Health Canada, 2017 | No change |  |
|  |  | BPS, 2010 | No change |  |
|  |  | DHA, 2018 | No change |  |
|  |  | KPS, 2017 | No change |  |
|  | Percentage of patients with opioid prescriptions | APS-AAPM, 2009 | No change | Casagrande (2023) |
|  | Percentage of patients with opioid prescriptions | CDC, 2016 | Negative | Casagrande (2023) |
|  | Opioid prescriptions per 100 persons | CDC, 2016 | Negative | Champagne-Langabeer (2021) |
|  | Number of opioid prescriptions | CDC, 2016 | Negative | Choi (2022) |
|  | Percentage of patients prescribed opioids | APS-AAPM, 2009 | Positive | Cohen (2022) |
|  |  | CDC, 2016 | No change |  |
|  | Number of individuals dispensed opioids | CPSBC, 2016 | Negative | Crabtree (2019) |
|  | Rate of prescriptions | BPS, 2010 | Positive | Curtis (2019) |
|  | Number of prescriptions dispensed | APS-AAPM, 2009 | Negative | Dart (2019) |
|  | Prevalence of opioid prescribing | BPS, 2010 | Positive | Davies (2019) |
|  | Number of opioid prescriptions | CDC, 2016 | Negative | Dryden (2022) |
|  | Percentage of Medicare enrollees who received opioids | CDC, 2016 | No change | Esechie (2021) |
|  | Percentage of patients prescribed any opioid at least once | BPS, 2010 | Positive | Foy (2016) |
|  | Number of opioid prescriptions (Schedule II + III + IV) | WA, 2007 | Negative | Franklin (2012) |
|  | Percentage of opioid users in population | APS-AAPM, 2009 | Positive | Garcia (2019)^3^ |
|  | Annual percent change in odds of receiving at least one opioid Rx | CDC, 2016 | Negative | Garcia (2019)^4^ |
|  | High-dose opioid dispensing rate | NOUGG, 2010 | Negative | Gomes (2014) |
|  | Prevalence of opioid prescriptions | CDC, 2016 | Positive | Gorfinkel (2022) |
|  | Number of opioid prescriptions | CDC, 2016 | Negative | Gupta (2020) |
|  | Dispensation of prescription opioids within past 30 days | CDC, 2016 | Negative | Hales (2020) |
|  | Number of opioids dispensed per 1000 population | SIGN, 2013 | Negative | Hebert (2022) |
|  | Number of prevalence chronic opioid users | CDC, 2016 | Negative | Hechter (2023) |
|  | Annual ambulatory visits involving opioid prescriptions per 10,000 visits | APS-AAPM, 2009 | Positive | Hirschtritt (2018) |
|  | Percentage of patients who use prescription opioids | CDC, 2016 | Negative | Hong (2022) |
|  | Opioid prescription claims per 100 beneficiaries among non-oncologists | CDC, 2016 | No change | Jairam (2021) |
|  | Number of prescriptions | APS-AAPM, 2009 | Negative | Kazanis (2018) |
|  | Number of patients who are persistent opioid users | CDC, 2016 | No change | Khouja (2022) |
|  | Number of opioid units dispensed | CDC, 2016 | Negative | Khouja (2021) |
|  | Number of opioid users prior to pancreatectomy | CDC, 2016 | Negative | Kluger (2022) |
|  | Percentage of patients who used opioids | APS-AAPM, 2009 | Positive | Kuo (2016) |
|  | Number of opioids dispensed per 1000 inhabitants | SIGN, 2013 | No change | Kurdi (2021) |
|  | Number of opioid prescriptions dispensed | APS-AAPM, 2009 | Positive | Levy (2015) |
|  | Opioid prescriptions per capita | APS-AAPM, 2009 | Positive | Levy (2015) |
|  | Opioid prescriptions | CMQ, 2009 | Negative | Ly (2025) |
|  | Proportion of members with any opioid prescriptions | CDC, 2016 | Negative | Liu (2023) |
|  | Number of prescriptions | CDC, 2016 | Negative | Meadows (2020) |
|  | Percentage of chronic opioid users | CDC, 2016 | Negative | Mehta (2021)^5^ |
|  | Percentage of adult population who used opioids | APS-AAPM, 2009 | Positive | Mojtabai (2018) |
|  | Percentage of beneficiaries with any opiate fill | APS-AAPM, 2009 | Negative | Morden (2014) |
|  | Percentage of opioid users | APS-AAPM, 2009 | Negative | Nahin (2019) |
|  | Percentage of office visits with opioid prescriptions | APS-AAPM, 2009 | Positive | Peckham (2019) |
|  | Proportion of workers with one or more opioid prescription | APS-AAPM, 2009 | Positive | Pensa (2018) |
|  | Proportion of older adults who are prescribed opioids | APS-AAPM, 2009 | Positive | Rhee (2019) |
|  | Opioid users | DHA, 2018 | Negative | Rolova (2025) |
|  | Opioid prescription claims per 100 providers | CDC, 2016 | Negative | Romman (2020) |
|  | Total prescriptions dispensed per 100,000 population | CDC, 2016 | Negative | Salvatore (2022) |
|  | Number of individuals dispensed opioids | CDC, 2016 | Negative | Salvatore (2022) |
|  | Number of prescriptions filled | CDC, 2016 | Negative | Scherrer (2020) |
|  | Proportion of all injured workers who are prescribed opioids | WA, 2010 | Negative | Sears (2021) |
|  | Prevalence of opioid prescriptions | CDC, 2016 | Negative | Sears (2020) |
|  | Number of opioid prescriptions | APS-AAPM, 2009 | Positive | Sites (2014) |
|  | Percentage of patients treated with opioids | CDC, 2016 | Negative | Smith (2020) |
|  | Percentage of patients who are prescribed opioids > 120 MME | CDC, 2016 | Negative | Suda (2022) |
|  | Total number of individuals dispensed opioids | WA, 2007 | Positive | Sullivan (2016) |
|  | Proportion of individuals dispensed opioids | WA, 2007 | Positive | Sullivan (2016) |
|  | Opioid units dispensed | WA, 2007 | Positive | Sullivan (2016) |
|  | Proportion of patients prescribed opioids | CDC, 2016 | Negative | Thompson (2024) |
|  | Total opioid prescriptions | BPS, 2010 | Positive | Torrance (2018) |
|  | Prevalence of chronic opioid use | CDC, 2016 | Negative | Weiner (2022) |
|  | Number of opioid units dispensed | APS-AAPM, 2009 | Negative | Woods (2021) |
|  |  | CDC, 2016 | Negative |  |
|  | Number of users per 1000 population | DPS, 2018 | Positive | Xie (2022) |
|  | Prevalence of opioid prescriptions | CDC, 2016 | Positive | Zhu (2019) |
| Incidence | Number of new long-term episodes | BPS, 2010 | Negative | Bedson (2016) |
|  | Percentage of Medicare beneficiaries with new opioid prescriptions | CDC, 2016 | Negative | Bhattacharya (2025) |
|  | Rate of people who initiate tapentadol | ANZCA, 2015 | Positive | Camacho (2024) |
|  | Number of opioid-naïve individuals dispensed an initial opioid prescription | CPSBC, 2016 | No change | Crabtree (2019) |
|  | Number of new users of extended-release opioids | CDC, 2016 | Negative | DiPrete (2024) |
|  | Number of incident chronic opioid users | CDC, 2016 | No change | Hechter (2023) |
|  | Number of opioid-naïve individuals dispensed an initial opioid prescription | APS-AAPM, 2009 | Negative (Optum, CCAE)  Positive (MDCD, MDCR) | Kern (2020) |
|  |  | CDC, 2016 | Negative (Optum, MDCD, MDCR)  Positive (CCAE) |  |
|  | Rate of new opioid prescriptions for adults not using benzodiazepine | APS-AAPM, 2009 | No change | Ladapo (2018) |
|  | Number of new LTOT beneficiaries | CDC, 2016 | Negative | Maharjan (2023) |
|  | Number of opioid-naïve individuals dispensed an initial opioid prescription | APS-AAPM, 2009 | Negative | Woods (2021) |
|  |  | CDC, 2016 | Negative |  |
|  | Number of opioid-naïve individuals dispensed an initial opioid prescription | CDC, 2016 | Negative | Zhu (2019) |
| Dosage | Prescription rate | COP, 2018 | Negative | Adalbert (2022) |
|  | MME per day | PMS, 2014 | Negative | Aronowitz (2021) |
|  | MME per day | CDC, 2016 | Negative | Aronowitz (2021) |
|  | Grams of opioids dispensed | APS-AAPM, 2009 | Positive | Atluri (2014) |
|  | MME per capita | CDC, 2016 | Negative | Aubry (2022) |
|  | Mean total MME per day | APS-AAPM, 2009 | Negative | Axeen (2018)^1^ |
|  | Average MME among Medicare beneficiaries with new opioid prescriptions | CDC, 2016 | Negative | Bhattacharya (2025) |
|  | MME per capita | CDC, 2016 | Negative | Bohnert (2018) |
|  | Oral morphine equivalent per 1000 population | BPS, 2010 | Positive | Curtis (2019) |
|  | DDD/1000 population/day | NOUGG, 2010 | Negative | Fischer (2018) |
|  | Average daily morphine equivalent dose | APS-AAPM, 2009 | Negative | Garcia (2019)^3^ |
|  | Rate of dispensing of long-acting opioids | NOUGG, 2010 | No change | Gomes (2017) |
|  | Average daily MME per prescription | APS-AAPM, 2009 | Negative | Guy (2017) |
|  | Mean dose of tramodol by year | GPS, 2009 | Positive | Hedenmalm (2019) |
|  | MME per person | CDC, 2016 | Negative | Hu (2025) |
|  | Defined daily doses of prescription opioids per million inhabitants per day | APS-AAPM, 2009 | Negative | Imtiaz (2020) |
|  |  | NOUGG, 2010 | Negative |  |
|  | DDD/1000 population/day | NOUGG, 2010 | No change | Jones (2021) |
|  |  | Health Canada, 2017 | No change |  |
|  | Average daily MME of first opioid prescription | APS-AAPM, 2009 | Negative (Optum)  Positive (MDCD, CCAE, MDCR) | Kern (2020) |
|  |  | CDC, 2016 | Negative (Optum, CCAE, MDCR)  Positive (MDCD) |  |
|  | Mean total MME prescribed in initial opioid prescription | APS-AAPM, 2009 | Positive (MDCD, CCAE, MDCR)  No change (Optum) | Kern (2020) |
|  |  | CDC, 2016 | Negative (Optum, CCAE, MDCR)  Positive (MDCD) |  |
|  | Raw number of doses | CDC, 2016 | Negative | Meadows (2020) |
|  | DDD/1000 population/day | S-MS, 2017 | Negative | Oliva (2022) |
|  | DDD/1000 population/day | S-MS, 2017 | Positive | Oliva (2023) |
|  | Total MME | APS-AAPM, 2009 | Negative | Piper (2018) |
|  | MME per 1000 inhabitants per day | DHA, 2018 | Negative | Rolova (2025) |
|  | Total MME | CDC, 2016 | Negative | Salvatore (2022) |
|  | Total MME per capita | NOUGG, 2010 | Positive | Smolina (2016) |
|  | Mean daily oral morphine equivalent dose | BPS, 2010 | Positive | Taqi (2023) |
|  | Total MME dispensed | APS-AAPM, 2009 | Negative | Woods (2021) |
|  |  | CDC, 2016 | Negative |  |
|  | Number of opioid units dispensed to opioid-naïve individuals with an initial opioid prescription | APS-AAPM, 2009 | Negative | Woods (2021) |
|  |  | CDC, 2016 | Negative |  |
|  | Oral morphine milligram equivalents per 1,000 population | DPS, 2018 | Positive | Xie (2022) |
|  | Average daily MME of initial opioid prescriptions | CDC, 2016 | Negative | Zhu (2021) |
|  | DDD/1000 population/day | PAS, 2013 | No change (oxycodone, pethidine)  Negative (morphine, fentanyl) | Zin (2020) |
| Duration | Mean total days of supply | APS-AAPM, 2009 | Negative | Axeen (2018)^1^ |
|  | Average days of supply | CDC, 2016 | Negative | Bhattacharya (2025) |
|  | Average days supplied | CDC, 2016 | Negative | Bohnert (2018) |
|  | Mean days of opioid supply | CDC, 2016 | Negative | Choudhry (2024) |
|  | Average number of days of supply per prescription | APS-AAPM, 2009 | No change | Guy (2017) |
|  | Average days of opioids supplied | APS-AAPM, 2009 | No change | Tehrani (2018) |
|  | Mean duration (in days) of tramodol by year | GPS, 2009 | Positive | Hedenmalm (2019) |
|  | Duration of initial opioid prescriptions | CDC, 2016 | Positive | Zhu (2019) |
| Tapering / Discontinuation | Abrupt discontinuation | CDC, 2016 | Positive | Beaugard (2022) |
|  | Tapered discontinuation | CDC, 2016 | Negative |  |
| **Chronic pain** | | | | |
| Prevalence | Proportion of inflammatory arthritides patients who are prescribed oral opioids | KPS, 2017 | No change | Ahn (2025) |
|  | Percentage of IBD patients receiving opioids | APS-AAPM, 2009 | Negative | Chhibba (2021) |
|  | Proportion of patients regularly on opioids | APS-AAPM, 2009 | No change | Curtis (2017) |
|  | Percentage of opioid dispensing within 7 days of discharge | ANZCA, 2015 | Negative | Gillies (2024) |
|  | Prevalence of opioid users | CDC, 2016 | Positive | Goudman (2024) |
|  | Percentage of patients prescribed opioids | APS-AAPM, 2009 | Positive | Han (2020) |
|  | Percentage of patients on long-term opioid therapy | CDC, 2016 | Negative | Holmer (2021) |
|  | Count of opioid prescriptions | CDC, 2016 | Negative | Iyengar (2024) |
|  | Number of Medicaid patients with anti-depression or anti-anxiety medications with opioid prescription | CDC, 2016 | Negative | Janakiram (2023) |
|  | Percentage of patients prescribed opioids | APS-AAPM, 2009 | No change | Kimmel (2017) |
|  | Percentage of patients with opioid prescriptions | CDC, 2016 | Negative | Kimmel (2024) |
|  | Prevalence of chronic opioid use in patients with RA | APS-AAPM, 2009 | Positive | Lee (2019) |
|  | Prevalence of opioid use in patients with knee osteoarthritis | APS-AAPM, 2009 | Negative | Losina (2023) |
|  | Percentage of chronic pain patients on long-term opioids prescribed by their family physicians | NOUGG, 2010 | Positive | May (2024) |
|  | Proportion of patients using opioids | Health Canada, 2017 | Negative | Molnar (2022) |
|  | Number of patients who have been prescribed opioids at least once | KPS, 2017 | Positive | Oh (2024) |
|  | Percentage of patients using opioids | CDC, 2016 | Negative | Paik (2021) |
|  | Prevalence of any opioid use | APS-AAPM, 2009 | No change | Richter (2017) |
|  | Percentage of veterans with cirrhosis who received opioid prescriptions | APS-AAPM, 2009 | Negative | Rogal (2019) |
|  | Annual prevalence of opioid analgesic prescriptions | BPS, 2010 | Negative | Scott (2022) |
|  | Percentage of encounters with opioids | CDC, 2016 | Negative | Turcotte (2024) |
|  | Prevalence of exposure to any opioid | CDC, 2016 | Negative | Whitney (2023) |
|  | Percentage of patients with rheumatoid arthritis who are prescribed opioids | APS-AAPM, 2009 | Positive | Zamora-Legoff (2016) |
| Incidence | Incidence of opioid users | CDC, 2016 | Positive | Goudman (2024) |
|  | Rate of new opioid prescriptions | CDC, 2016 | Negative | Leja (2022) |
|  | Proportion of patients that are opioid naïve | Health Canada, 2017 | Negative | Molnar (2022) |
| Dosage | Total MME | APS-AAPM, 2009 | Negative | Daubresse (2019) |
|  | Total oral morphine equivalents | CDC, 2016 | Negative | Premkumar (2022) |
|  | MME per prescription | CDC, 2016 | Negative | Turcotte (2024) |
| Duration | Mean days supplied | APS-AAPM, 2009 | Negative | Daubresse (2019) |
|  | Total annual days’ supply of opioids | APS-AAPM, 2009 | Positive | Han (2020) |
| **Chronic Non-Cancer Pain** | | | | |
| Prevalence | Percentage of individuals with chronic non-cancer pain having received at least one opioid prescription | CDC, 2016 | Negative | Bandara (2022) |
|  | Mean number of opioid prescriptions dispensed per person | CDC, 2016 | Negative |  |
|  | Percentage of patients with musculoskeletal condition receiving long-term opioid prescription | ANZCA, 2015 | Positive | Black-Tiong (2021) |
|  | Percent of patients with at least 1 prescription 3 months before index date | DHA, 2017 | Negative | Bruun (2024) |
|  | Percentage of patients receiving long-term opioid treatment | APS-AAPM, 2009 | No change | Chen (2019) |
|  | Proportion of pain visits | APS-AAPM, 2009 | Negative | Daubresse (2013) |
|  | Number of opioid users | APS-AAPM, 2009 | Positive | Denis (2019) |
|  |  | CDC, 2016 | Positive |  |
|  | Individuals with at least one opioid prescription dispensed | WA, 2007 | Positive | Fulton-Kehoe (2015) |
|  | Opioid dispensing rate per 100 persons | CDC, 2016 | Negative | Kang (2024) |
|  | Annual prevalence of patients receiving >= 50 MME of opioids and high-risk opioids | APS-AAPM, 2009 | Negative | Kuo (2024) |
|  |  | CDC, 2016 | No change |  |
|  | Percentage of chronic pain visits with opioids prescribed | APS-AAPM, 2009 | Negative | Larochelle (2015) |
|  | Prevalence of opioid use among people without cancer | ANZCA, 2015 | Positive | Lalic (2018) |
|  | Prevalence of chronic opioid use | CDC, 2016 | No change | Mehta (2021)^6^ |
|  | Prevalence of non-injectable opioid analgesics in noncancer patients | KPS, 2017 | Positive | Noh (2022) |
|  | Percentage of individuals with opioid prescription dispensed | CDC, 2016 | Positive | Pritchard (2022) |
|  | Proportion of patients with non-chronic opioid dispensing | CDC, 2016 | Negative | Roy (2024) |
|  | Percentage of adults aged ≥20 years who had an opioid prescription filled | APS-AAPM, 2009 | Positive | Schieber (2020) |
|  | Percentage of adults aged ≥20 years who had an opioid prescription filled | CDC, 2016 | Negative | Schieber (2020) |
|  | Percentage of elderly Medicare beneficiaries with chronic pain conditions who used opioids | APS-AAPM, 2009 | Positive | Shen (2018) |
|  | Prevalence of long-term opioid use | KPS, 2017 | Positive | Song (2022) |
|  | Prevalence of prescription opioid use | APS-AAPM, 2009 | No change | Stokes (2019) |
|  | Prevalence of opioid use among people with spinal cord injury | CDC, 2016 | Negative | Strong (2024) |
| Incidence | Percentage of patients with musculoskeletal condition receiving long-term opioid prescription | ANZCA, 2015 | Negative | Black-Tiong (2021) |
|  | Percentage of patients receiving an opioid who were opioid naïve | CDC, 2016 | Positive | Goldstick (2021) |
|  | Rate of opioid prescriptions among first-time presenters for neck pain | CDC, 2016 | Negative | Jami (2021) |
|  | Incidence of opioid use among people without cancer | ANZCA, 2015 | Negative | Lalic (2018) |
|  | Proportion of patients with opioid initiation | CDC, 2016 | Negative | Lin (2023) |
|  | Incidence rate of opioid use | NOUGG, 2010 | Positive | Marrie (2023) |
|  | Percentage of new medication users on opioid monotherapy | APS-AAPM, 2009 | No change | Surbhi (2025) |
|  |  | CDC, 2016 | No change |  |
|  | Rate of first-time extended-release opioid prescriptions | CDC, 2016 | No change | Togun (2021) |
|  | Predicted incidence rate of one or more opioid prescription fill | CDC, 2016 | Negative | Townsend (2021) |
|  | Individuals with new opioid prescription | CDC, 2016 | Negative | Tucker (2021) |
|  | Initial analgesic prescription among patients with incident osteoarthritis | BPS, 2010 | Negative | Zeng (2021) |
| Dosage | Total MME | APS-AAPM, 2009 | Positive | Axeen (2018)^2^ |
|  | Mean MME/day among opioid prescriptions per person | CDC, 2016 | Negative | Bandara (2022) |
|  | Daily MME | CDC, 2016 | No change | Bao (2021) |
|  | MME/population | APS-AAPM, 2009 | Positive | Hauser (2017) |
|  | MME/population | NOUGG, 2010 | Positive | Hauser (2017) |
|  | MME/population | GPS, 2009 | Positive | Hauser (2017) |
|  | Total MME per person | CDC, 2016 | Negative | Kang (2024) |
|  | Mean daily MME per prescription | CDC, 2016 | Negative | Kang (2024) |
|  | Mean daily MME | CDC, 2016 | No change | Maierhofer (2023) |
|  | MME per person | CPSBC, 2016 | Negative | Panagiotoglou (2025) |
|  | Average MME per day | CDC, 2016 | Negative | Salas (2021) |
| Duration | Mean number of days per year with opioid prescriptions per person | CDC, 2016 | Negative | Bandara (2022) |
|  | Average time on long-term opioid Rx among incident cases | ANZCA, 2015 | Negative | Black-Tiong (2021) |
|  | Mean prescription duration in days | CDC, 2016 | Negative | Goldstick (2021) |
|  | Days supplied per prescription | CDC, 2016 | Negative | Kang (2024) |
|  | Mean days of supply | CDC, 2016 | Negative | Maierhofer (2023) |
| Tapering / Discontinuation | Percent of patients undergoing tapering of opioids | CDC, 2016 | Positive | Fenton (2019) |
|  | Tapering in opioid prescription dose | CDC, 2016 | Positive | Mazurenko (2021) |
|  | Rapid discontinuation | CDC, 2016 | Positive | Mazurenko (2021) |
|  | Percent of long-term opioid therapy users who discontinued | CDC, 2016 | Positive | Neprash (2021) |
|  | Aggressive tapering | CPSBC, 2016 | Negative | Panagiotoglou (2025) |
|  | Opioid discontinuation among LTOT users | CDC, 2016 | No change | Wei (2022) |
| **Cancer Pain** | | | | |
| Prevalence | Percentage of individuals with cancer pain having received at least one opioid prescription | CDC, 2016 | Negative | Bandara (2022) |
|  | Mean number of opioid prescriptions per person | CDC, 2016 | Positive | Bandara (2022) |
|  | Percentage of individuals dispensed opioids | CDC, 2016 | Negative | Chen (2022) |
|  | Opioid prescription claims per 100 beneficiaries among oncologists | CDC, 2016 | No change | Jairam (2021) |
|  | Prevalence of opioid use among people with cancer | ANZCA, 2015 | Negative | Lalic (2018) |
|  | Prevalence of opioid use | APS-AAPM, 2009 | No change | Nelson (2019) |
|  | Proportion of people prescribed opioids | CDC, 2016 | Negative | Rodin (2025) |
| Incidence | Long-term opioid therapy rate | APS-AAPM, 2009 | No change | Gibson (2021) |
|  | Incidence of opioid use among people with cancer | ANZCA, 2015 | No change | Lalic (2018) |
| Dosage | Mean MME/day among opioid prescriptions per person | CDC, 2016 | Negative | Bandara (2022) |
|  | Daily MME | CDC, 2016 | No change | Bao (2021) |
|  | MME/day per person | CDC, 2016 | Negative | Chen (2022) |
|  | MME per person | CPSBC, 2016 | Negative | Panagiotoglou (2025) |
| Duration | Mean number of days per year with opioid prescriptions per person | CDC, 2016 | Negative | Bandara (2022) |
|  | Days of supply per person | CDC, 2016 | Negative | Chen (2022) |
|  | Days of supply per person | CDC, 2016 | Negative | Chen (2022) |
| **Acute Pain** | | | | |
| Prevalence | Prevalence of opioid use | CDC, 2016 | Negative | Bhargava (2022) |
|  | Proportion of ED discharges with opioid prescription | CDC, 2016 | Negative | Gleber (2020) |
|  | Number of Medicaid patients with anti-depression or anti-anxiety medications with opioid prescription | CDC, 2016 | Negative | Janakiram (2023) |
|  | Total number of prescriptions per 100 motor vehicle crashes injuries | CDC, 2016 | Negative | Jin (2022) |
|  | Number of prescriptions | CDC, 2016 | Negative | Jin (2022) |
|  | Percentage of patients with post-operative opioid use | APS-AAPM, 2009 | Negative | Labaran (2020) |
|  | Percentage of visits with opioids prescribed | APS-AAPM, 2009 | No change | Larochelle (2015) |
|  | Percentage of GI visits with opioid prescriptions | APS-AAPM, 2009 | Positive | LeBrett (2021) |
|  | Percentage of patients who are prescribed opioids after ambulatory surgery | CDC, 2016 | No change | Liu (2024) |
|  | Prevalence of chronic opioid use | CDC, 2016 | No change | Mehta (2021)^6^ |
|  | Percentage of individuals dispensed opioids with surgical pain | CDC, 2016 | Positive | Pritchard (2022) |
|  | Percentage of patients receiving opioid prescriptions | CDC, 2016 | Negative | Rancu (2025)^7^ |
|  | Percentage of patients receiving opioid prescriptions | CDC, 2016 | Negative | Rancu (2025)^8^ |
|  | Percentage of ED visits with opioid RX | APS-AAPM, 2009 | Negative | Rui (2020) |
|  | Percentage of surgery discharges that involved opioid prescriptions | CDC, 2016 | Negative | Thiels (2019) |
|  | Prevalence of persistent opioid use | CDC, 2016 | Negative | Zaveri (2020) |
| Incidence | Percentage of patients who filled 1+ opioid prescription within 1 year of surgery | NOUGG, 2010 | No change | Canizares (2023) |
|  | New persistent opioid use | APS-AAPM, 2009 | Negative | Mauck (2024) |
|  | Annual proportion of surgical patients prescribed one or more slow-release opioids on discharge | ANZCA, 2015 | Negative | Wembridge (2024) |
| Dosage | Mean 90-day post-operative MME | CDC, 2016 | Negative | Day (2023) |
|  | Average MME per day | CDC, 2016 | Negative | Dayer (2019) |
|  | Total MME per prescription | CDC, 2016 | Positive | Jin (2022) |
|  | Daily MME per prescription | CDC, 2016 | Negative | Jin (2022) |
|  | Mean morphine equivalents prescribed at discharge | CDC, 2016 | Negative | Karst (2019) |
|  | Mean MME Opioid Dose | Health Canada, 2017 | Negative | Kirubalingam (2022) |
|  | Post-operative MME prescribed | CDC, 2016 | Negative | Lazar (2021) |
|  | Mean oral morphine equivalent | CDC, 2016 | Negative | Liu (2024) |
|  | Mean daily MME | CDC, 2016 | Negative | Maierhofer (2023) |
|  | Mean post-operative MME | CDC, 2016 | Negative | Moawad (2021) |
|  | Number of post-operative opioid pills prescribed per person | CDC, 2016 | Negative | Nobel (2019) |
|  | Mean MME per patient | CDC, 2016 | Negative | Rancu (2025)^7^ |
|  | Mean MME per patient | CDC, 2016 | Negative | Rancu (2025)^8^ |
|  | First prescription amount, opioids dispensed | CDC, 2016 | Negative | Sutherland (2021) |
|  | Total opioids dispensed within 30 days of surgery | CDC, 2016 | Negative | Sutherland (2021) |
|  | Annual surgical discharge prescription oral morphine equivalent daily dose | ANZCA, 2015 | Negative | Wembridge (2024) |
| Duration | Average duration of supply | CDC, 2016 | Negative | Dayer (2019) |
|  | Prescription duration | CDC, 2016 | Positive | Jin (2022) |
|  | Mean days of supply | CDC, 2016 | Negative | Maierhofer (2023) |
|  | Duration of first prescription | CDC, 2016 | Negative | Sutherland (2021) |
|  | Mean days of opioids prescribed for patients | APS-AAPM, 2009 | Positive | Thompson (2018) |
| **Palliative Pain** | | | | |
| Prevalence | Opioid prescription claims per 100 beneficiaries among palliative care specialists (non-oncologists) | CDC, 2016 | Positive | Jairam (2021) |
| Dosage | MME per person | CPSBC, 2016 | Negative | Panagiotoglou (2025) |

^1^ Axeen S., Trends in Opioid Use and Prescribing in Medicare, 2006-2012. Health Serv Res. 2018;53(5):3309-2810.1111/1475-6773.12846.

^2^ Axeen, S., Seabury, S.A. & Menchine, M. Emergency Department Contribution to the Prescription Opioid Epidemic. Ann Emerg Med 71, 659-667 e653 (2018).

^3^ Garcia, M. M., et al. "Impact of Sequential Opioid Dose Reduction Interventions in a State Medicaid Program Between 2002 and 2017." J Pain **20**(8): 876-884.

^4^ Garcia, M. C., et al. "Opioid Prescribing Rates in Nonmetropolitan and Metropolitan Counties Among Primary Care Providers Using an Electronic Health Record System - United States, 2014-2017." MMWR Morb Mortal Wkly Rep **68**(2): 25-30.

^5^ Mehta, H.B., et al. Time Trends in Opioid Use by Dementia Severity in Long-Term Care Nursing Home Residents. J Am Med Dir Assoc 22, 124-131.e121 (2021).

^6^ Mehta, H.B., et al. State Variation in Chronic Opioid Use in Long-Term Care Nursing Home Residents. J Am Med Dir Assoc **22**(12): 2593-2599 e2594.

^7^ Rancu et al., Opioid Prescribing Trends Following Lumbar Discectomy. J Am Acad Orthop Surg. 2024 Dec 19;33(18):1054-1059.

^8^ Rancu et al., Opioid Use Following Open Reduction and Internal Fixation of Mandibular Fractures. The Journal of craniofacial surgery. 36. 10.1097/SCS.0000000000010930. (2025)

Appendix C: Meta-analysis

Chronic Pain Population

For chronic pain patients (**Figure S2**), guidelines did not have an immediate impact on the prevalence (-3.7%, 95% CI: -9.0% to 2.0%), but led to a 3.4% yearly decline (95% CI: -5.3% to -1.5%) in the prevalence of people who were prescribed opioids (N = 12 from 14 different patient populations).^73-84^

General Population

The prescribing guidelines did not have a strong effect on the level (1.4%, 95% CI: -7.0% to 10.6%) and trend changes (-8.7%, 95% CI: -24.4% to 10.2%) in the incidence of opioid use (N = 4; **Figure S3**).^85-88^ There were limited level (-0.9%, 95% CI: -4.5% to 2.8%) changes, but there was a 2.8% yearly decline (-2.8%, 95% CI: -4.8% to -0.9%) in MME per day prescribed (N = 8; **Figure S4**).^85,87-93^ Based on three studies,^86,94,95^ there were limited level changes in the number of prevalent users of prescription opioids (8.0%, 95% CI: -3.3% to 20.7%), but there was a 14.6% yearly decline (95% CI: -20.2% to -8.7%) in the number of prevalent users post-guideline implementation (**Figure S5**). Following guideline implementation, there was weak evidence of level change (2.3%, 95% CI: -0.6% to 5.2%) and a strong declining yearly trend (-10.1%, 95% CI: -14.1% to -6.0%) in the number of opioid prescriptions (N = 12; **Figure S6**).^85,89,92,96-104^

Acute Pain Population

For acute pain patients (**Figure S7**), guidelines did not have an effect on the level (-5.9%, 95% CI: -13.7% to 2.6%) and trend changes (-2.9%; 95% CI: -11.2% to 6.1%) in the prevalence of opioid use (N = 3).^105-107^

Figure S2. Forest plot of the percentage changes in level and trend for the prevalence of patients who are prescribed opioids following guideline implementation (chronic pain population)


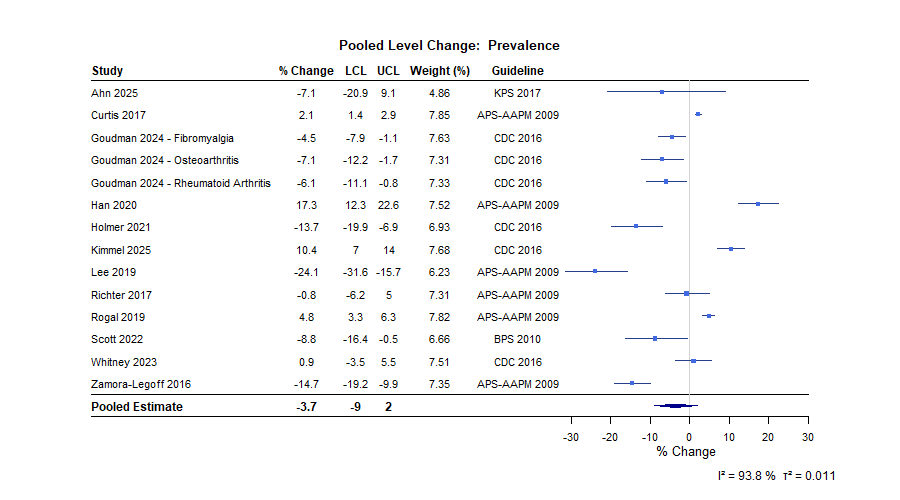

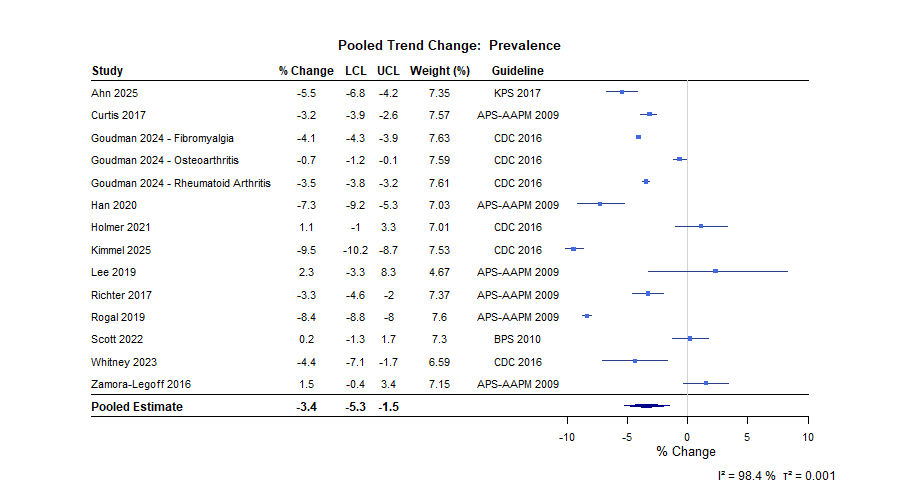


Abbreviation: APS-AAPM = American Pain Society-American Association of Pain Medicine; BPS = British Pain Society; CDC = Centers for Disease Control and Prevention; KPS = Korean Pain Society; LCL = Lower confidence limit; UCL = Upper confidence limit

Figure S3. Forest plot of the percentage changes in level and trend for the incidence proportion of patients who are prescribed opioids following guideline implementation


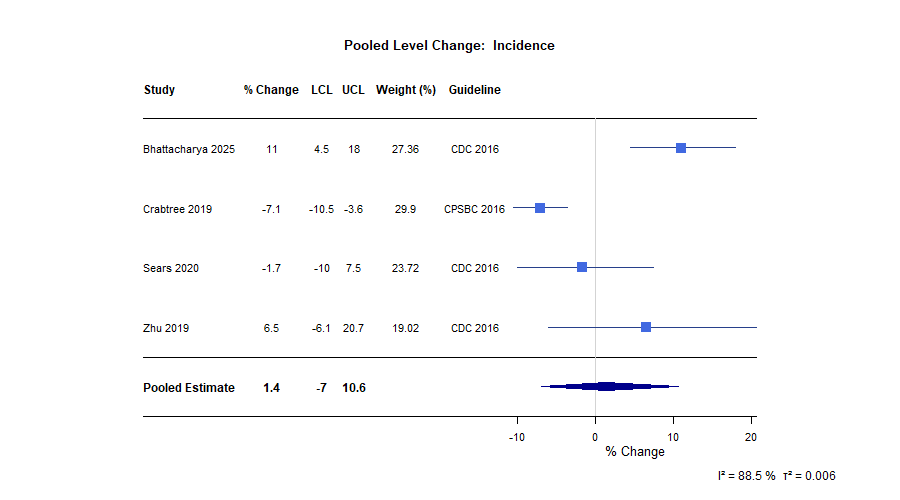

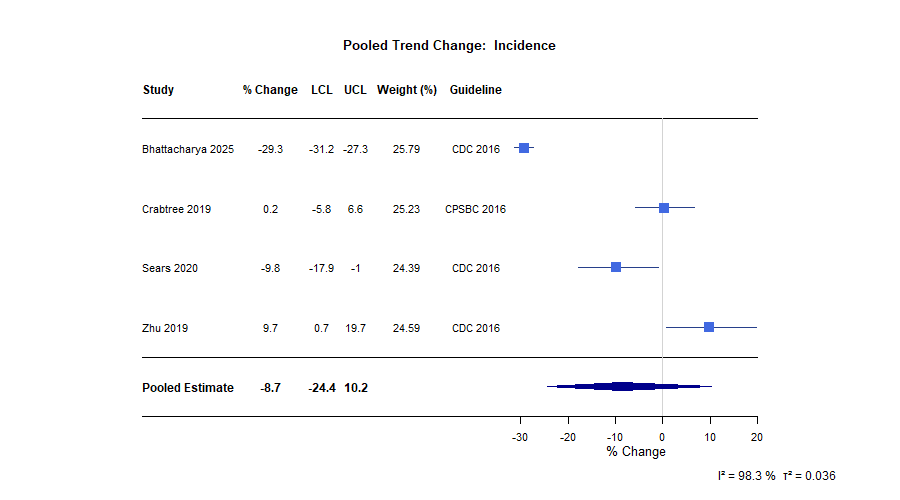


Abbreviation: CDC = Centers for Disease Control and Prevention; CPSBC = College of Physicians and Surgeons of British Columbia; LCL = Lower confidence limit; UCL = Upper confidence limit

Figure S4. Forest plot of the percentage changes in level and trend for morphine milligram equivalent dosage per day following guideline implementation


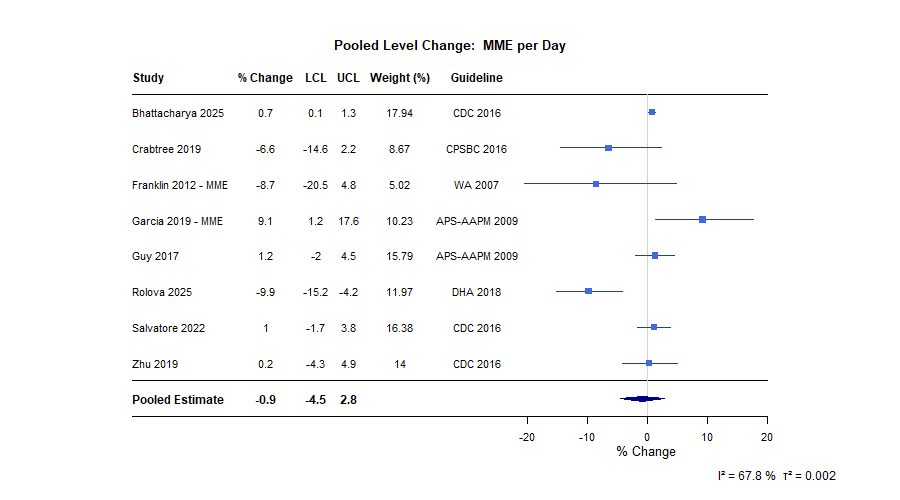

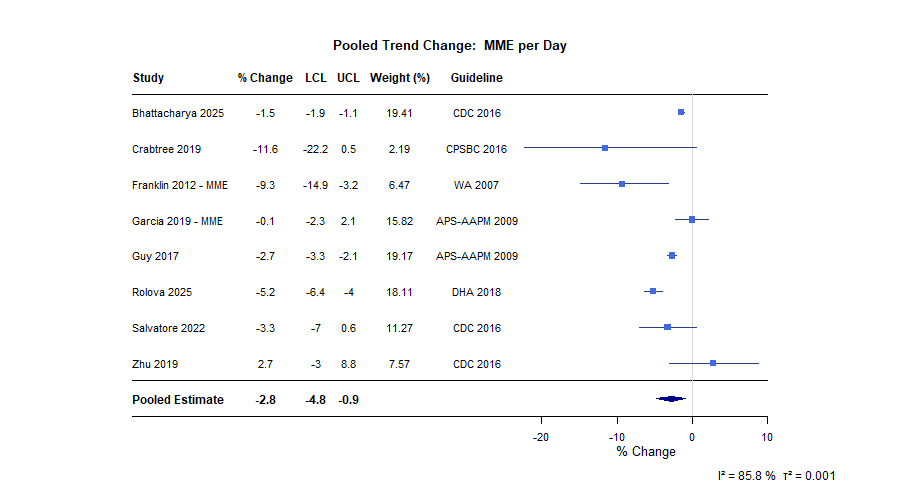


Abbreviation: APS-AAPM = American Pain Society-American Association of Pain Medicine; CDC = Centers for Disease Control and Prevention; CPSBC = College of Physicians and Surgeons of British Columbia; DHA = Danish Health Authority; LCL = Lower confidence limit; UCL = Upper confidence limit; WA = Washington state

Figure S5. Forest plot of the percentage changes in level and trend for the prevalent users of opioids following guideline implementation


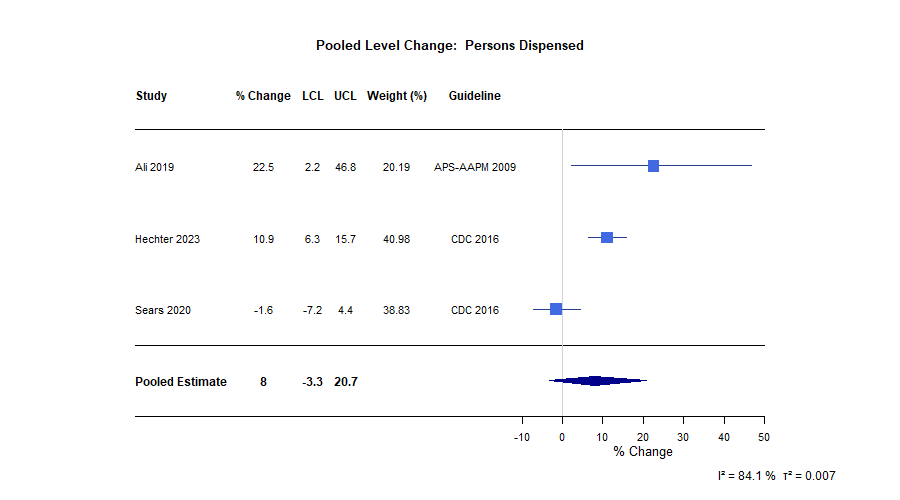

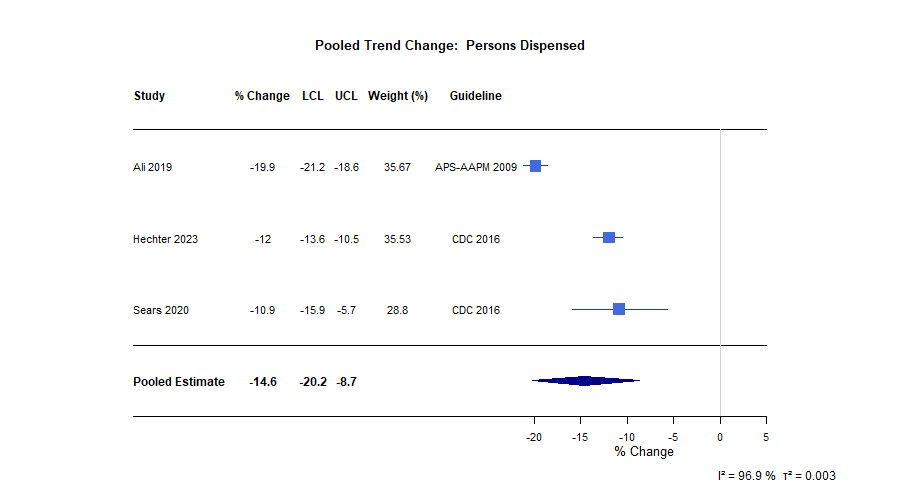


Abbreviation: APS-AAPM = American Pain Society-American Association of Pain Medicine; CDC = Centers for Disease Control and Prevention; LCL = Lower confidence limit; UCL = Upper confidence limit

Figure S6. Forest plot of the percentage changes in level and trend for the number of opioid prescriptions following guideline implementation


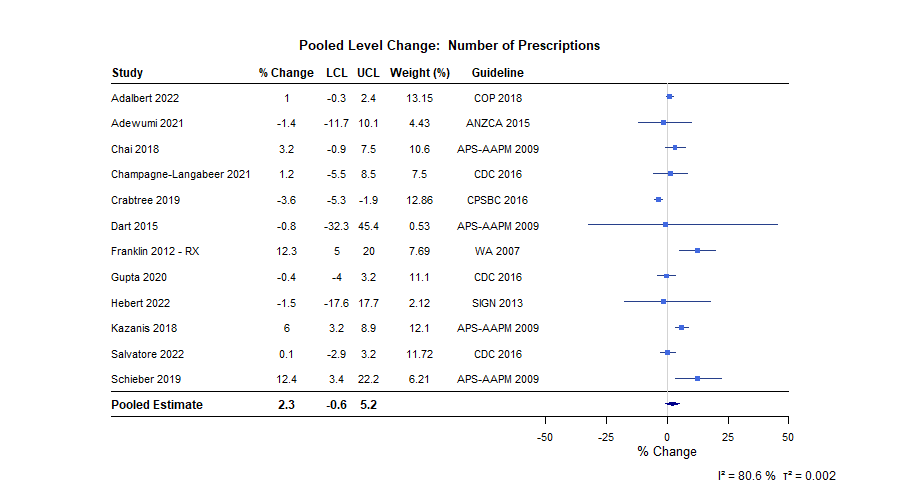

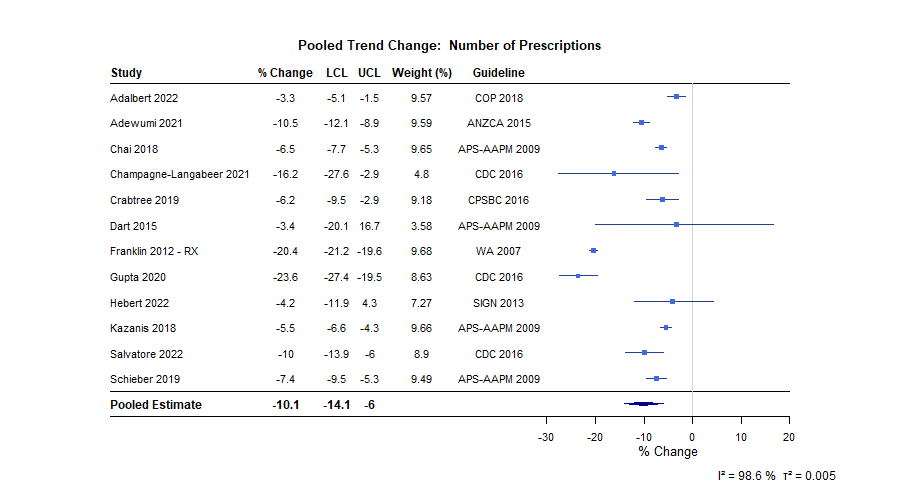


Abbreviation: APS-AAPM = American Pain Society-American Association of Pain Medicine; ANZCA = Australian and New Zealand College of Anaesthetists; CDC = Centers for Disease Control and Prevention; COP = Commonwealth of Pennsylvania; CPSBC = College of Physicians and Surgeons of British Columbia; LCL = Lower confidence limit; SIGN = Scottish Intercollegiate Guidelines Network; UCL = Upper confidence limit; WA = Washington state

Figure S7. Forest plot of the percentage changes in level and trend for the prevalence of patients who are prescribed opioids following guideline implementation (acute pain population)


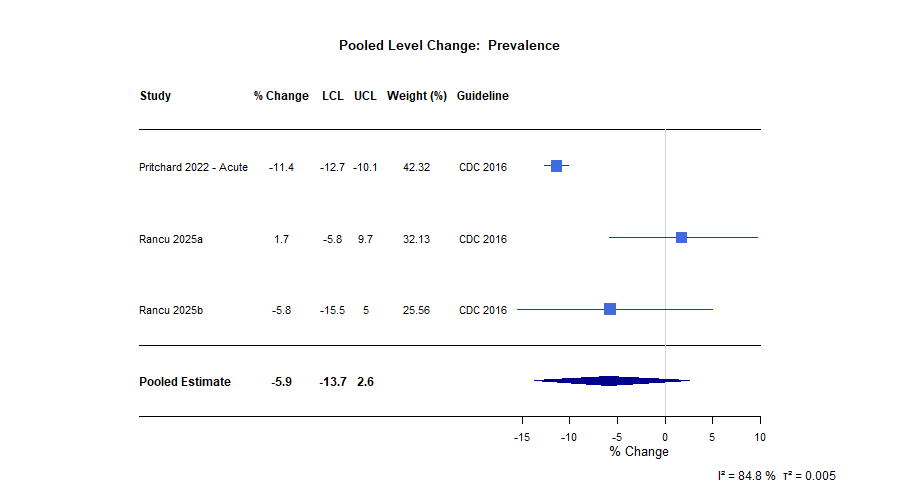

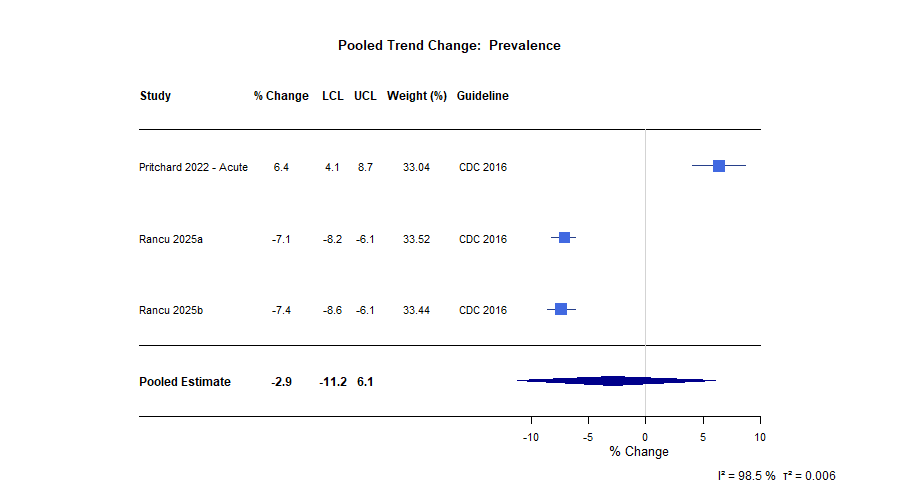


Abbreviation: CDC = Centers for Disease Control and Prevention; LCL = Lower confidence limit; UCL = Upper confidence limit

Rancu et al. (2025)^a^: Rancu AL, Gouzoulis MJ, Winter AD, Katsnelson BM, Ansah-Twum JK, Grauer JN. Opioid Prescribing Trends Following Lumbar Discectomy. J Am Acad Orthop Surg. 2024 Dec 19;33(18):1054-1059. doi: 10.5435/JAAOS-D-24-00908. PMID: 39706160.

Rancu et al. (2025)^b^: Rancu, Albert & Salib, Andrew & Kammien, Alexander & Lizardi, Juan & Allam, Omar & Grauer, Jonathan & Alperovich, Michael. (2024). Opioid Use Following Open Reduction and Internal Fixation of Mandibular Fractures. The Journal of craniofacial surgery. 36. 10.1097/SCS.0000000000010930.

REFERENCES

1 American College of Occupational and Environmental Medicine. ACOEM Guidelines for Chronic Use of Opioids. (Elk Grove Village, IL, 2008).

2 American College of Occupational and Environmental Medicine. Guidelines for chronic use of opioids. (2011).

3 Hegmann, K. T. *et al.* ACOEM practice guidelines: opioids for treatment of acute, subacute, chronic, and postoperative pain. *Journal of occupational and environmental medicine* **56**, e143-e159 (2014).

4 Arizona Department of Health Services. *Arizona opioid prescribing guidelines*, <<http://www.azdhs.gov/documents/audiences/clinicians/clinical-guidelines-recommendations/prescribing-guidelines/az-opiod-prescribing-guidelines.pdf>> (2014).

5 Analgesic Expert Group. *Therapeutic Guidelines: Analgesic*. (Therapeutic Guidelines Ltd, 2012).

6 American Geriatrics Society Panel on Persistent Pain in Older Persons. Panel on Persistent Pain in Older Persons. The management of persistent pain in older adults. *J Am Geriatr Soc* **50**, 205-224 (2002).

7 American Geriatrics Society Panel on Pharmalogical Management of Persistent Pain in Older Persons. Pharmacological management of persistent pain in older persons. *J Am Geriatr Soc* **57**, 1331-1346 (2009).

8 Australian and New Zealand College of Anaesthetists Faculty of Pain Medicine. *Principles regarding the use of opioid analgesics in patients with chronic noncancer pain*, <<https://web.archive.org/web/20120321090606/https://www.fpm.anzca.edu.au/resources/professional-documents/documents/PM1%202010.pdf>> (2010).

9 American Academy of Pain Medicine, A. P. S. The use of opioids for the treatment of chronic pain. A consensus statement from the American Academy of Pain Medicine and the American Pain Society. *Clin J Pain* **13**, 6-8 (1997).

10 Wilson, P. R. *et al.* Practice guidelines for chronic pain management: a report by the American Society of Anesthesiologists Task Force on Pain Management, Chronic Pain Section. *Anesthesiology* **86**, 995-1004 (1997).

11 American Society of Anesthesiologists Task Force on Chronic Pain Management. Practice guidelines for chronic pain management: an updated report by the American Society of Anesthesiologists Task Force on Chronic Pain Management and the American Society of Regional Anesthesia and Pain Medicine. *Anesthesiology* **112**, 810-833 (2010).

12 American Society of Interventional Pain Physicians. (2000).

13 Trescot, A. M. *et al.* Opioid guidelines in the management of chronic non-cancer pain. *Pain physician* **9**, 1 (2006).

14 Trescot, A. M. *et al.* Opioids in the management of chronic non-cancer pain: an update of American Society of the Interventional Pain Physicians' (ASIPP) Guidelines. *Pain Physician* **11**, S5-s62 (2008).

15 Manchikanti, L. *et al.* American Society of Interventional Pain Physicians (ASIPP) guidelines for responsible opioid prescribing in chronic non-cancer pain: Part 2--guidance. *Pain physician* **15** (2012).

16 Manchikanti, L. *et al.* Responsible, safe, and effective prescription of opioids for chronic non-cancer pain: American Society of Interventional Pain Physicians (ASIPP) guidelines. *Pain physician* **20**, S3 (2017).

17 Australian Pain Society. *Pain in residential aged care facilities: management strategies*. (Australian Pain Society, 2021).

18 California Medical Board. Prescribing Controlled Substances for Pain. (1994).

19 Rieb, L. M. *et al.* Canadian guidelines on opioid use disorder among older adults. *Canadian Geriatrics Journal* **23**, 123 (2020).

20 Dowell, D. CDC clinical practice guideline for prescribing opioids for pain—United States, 2022. *MMWR. Recommendations and reports* **71** (2022).

21 Colorado Department of Regulatory Affairs. Policy for prescribing and dispensing opioids. (2014).

22 Colorado Department of Regulatory Affairs. Policy for prescribing and dispensing opioids. (2018).

23 Colorado Department of Regulatory Affairs. *Policy for prescribing and dispensing opioids*, <<https://drive.google.com/file/d/19xrPqsCbaHHA9nTD1Fl3NeCn5kwK60zR/view>> (2019).

24 Colorado Division of Workers' Compensation. *Chronic Pain Disorder Medical Treatment Guidelines*, <<https://cdle.colorado.gov/sites/cdle/files/Ex9_CPD_InText_Biblio.pdf>> (2017).

25 Commonwealth of Pennsylvania & Pennsylvania Pharmacists Association. *Prescribing Guidelines for Pennsylvania. Opioid Dispensing Guidelines*, <<https://www.pa.gov/content/dam/copapwp-pagov/en/health/documents/topics/documents/opioids/PA%20Guidelines%20on%20the%20Dispensing%20of%20Opioids.pdf>> (2016).

26 Jovey, R. D. *et al.* Use of opioid analgesics for the treatment of chronic noncancer pain-a consensus statement and guidelines from the Canadian Pain Society, 2002. (2003).

27 Hagen, N., Flynne, P., Hays, H. & MacDonald, N. Guidelines for managing chronic non-malignant pain. Opioids and other agents. College of Physicians and Surgeons of Alberta. *Canadian Family Physician* **41**, 49 (1995).

28 Danish Health Authority. *Kortlægning af opioidforbruget i Danmark. Med fokus på patienter med kroniske non-maligne smerter* <<https://www.sst.dk/da/udgivelser/2016/Kortlaegning-af-opioidforbruget-i-Danmark>> (2016).

29 Henrard, G. *et al.* Aanpak van chronische pijn in de eerste lijn. (2017).

30 Häuser, W. *et al.* European* clinical practice recommendations on opioids for chronic noncancer pain–Part 1: Role of opioids in the management of chronic noncancer pain. *European Journal of Pain* **25**, 949-968 (2021).

31 Fine, P. G., Portenoy, R. K. & on Evidence, A. H. E. P. Establishing “best practices” for opioid rotation: conclusions of an expert panel. *Journal of pain and symptom management* **38**, 418-425 (2009).

32 Hunter Integrated Pain Service. Reconsidering opioid therapy. (2013).

33 Velferðarráðuneytið. *Aðgerðir til að sporna við misnotkun lyfja sem geta valdið ávana og fíkn*, <<https://www.stjornarradid.is/lisalib/getfile.aspx?itemid=3d1a8517-5f66-11e8-942c-005056bc530c>> (2018).

34 Committee for the Guidelines for Prescribing Opioid Analgesics for Chronic Noncancer Pain of JSPC. Guidelines for prescribing opioid analgesics for chronic noncancer pain. 20-23 (Sinko Trading Co.. Ltd., Tokyo, 2012).

35 Sumitani, M. *et al.* Executive summary of the Clinical Guidelines of Pharmacotherapy for Neuropathic Pain: second edition by the Japanese Society of Pain Clinicians. *Journal of Anesthesia* **32**, 463-478 (2018). <https://doi.org/10.1007/s00540-018-2501-0>

36 Kalso, E. *et al.* Recommendations for using opioids in chronic non-cancer pain. *European Journal of Pain* **7**, 381-386 (2003).

37 Garcia, J. B. S. *et al.* Latin American Pain Federation position paper on appropriate opioid use in pain management. *Pain Reports* **4**, e730 (2019).

38 Medical Board of California. *Guidelines for prescribing controlled substances for pain*, <<https://web.archive.org/web/20141127060036/http://www.mbc.ca.gov/Licensees/Prescribing/Pain_Guidelines.pdf>> (2014).

39 Medical Board of California. *Guidelines for prescribing controlled substances for pain*, <<https://www.mbc.ca.gov/Download/Publications/pain-guidelines.pdf>> (2023).

40 Kalso, E., Paakkari, P. & Stenberg, I. *Opioids in chronic non-cancer pain, situation and guidelines in Nordic countries*. (National Agency for Medicines, 1999).

41 Norwegian Directorate of Health. National Guidelines: The use of opioids in the treatment of long-lasting non-malignant pain. (2014).

42 Norwegian Directorate of Health. Nasjonal veileder for bruk av opioider ved langvarige ikke-kreftrelaterte smerter. (2015).

43 Norwegian Directorate of Health. National recommendation for opioid treatment for chronic non-cancer pain. (2016).

44 Norwegian Directorate of Health. *Addictive Drugs, 8. Opioids*, <<https://www.helsedirektoratet.no/veiledere/vanedannende-legemidler/opioider#ved-vurdering-av-behandling-med-opioider-utover-fire-uker-skal-legen-gjore-en-konkret-vurdering-av-indikasjon-nytte-og-risiko-ved-behandlingen>> (2021).

45 Pijn, N. NHG-standaard Pijn. *Huisarts Wet* **58**, 472-485 (2015).

46 Nederlands Huisartsen Genootschap. *Pain*, <<https://richtlijnen.nhg.org/standaarden/pijn#volledige-tekst-beleid-bij-chronische-pijn>> (2018).

47 NHS. *Opioid deprescribing for persistent non-cancer pain*, <<https://www.nottsapc.nhs.uk/media/1564/opioid_deprescribing.pdf>> (2020).

48 NHS. *Opioid deprescribing for persistent non-cancer pain*, <<https://www.nottsapc.nhs.uk/media/1564/opioid_deprescribing.pdf>> (2023).

49 National Institute for Health Care Excellence. *Chronic pain (primary and secondary) in over 16s: assessment of all chronic pain and management of chronic primary pain*. (National Institute for Health and Care Excellence, 2021).

50 Norwegian Medicines Agency. *Treatment recommendation: Use of opioids in chronic non-malignant pain—an, update*. (Norwegian Medicines Agency, 2008).

51 Pennsylvania Department of Health & Pennsylvania Department of Drug and Alcohol Problems. *Pennsylvania Prescription Drug Monitoring Program (PDMP) System User and Stakeholder Training. Opioid Prescribing Guide*, <<https://www.pa.gov/content/dam/copapwp-pagov/en/health/documents/topics/documents/programs/pdmp/4-PDMP_OpioidPrescribing-Guide_F.pdf>> (2017).

52 Ackermann, E., Litt, J. & Morgan, M. Prescribing drugs of dependence in general practice, Part C2: the role of opioids in pain management. (2017).

53 Kraychete, D. C., Siqueira, J. T. T. d. & Garcia, J. B. S. Recommendations for the use of opioids in Brazil: part I. *Revista Dor* **14**, 295-300 (2013).

54 Ministry of Health Singapore. *National Guidelines for the Safe Prescribing of Opioids 2021*, <<https://web.archive.org/web/20230330012819/https://www.moh.gov.sg/docs/librariesprovider5/default-document-library/national-guidelines-for-the-safe-prescribing-of-opioids-2021.pdf>> (2021).

55 Swedish Medical Products Agency. Anva﻿̈ndning av opioider vid la﻿̊ngvarig icke-cancerrelaterad sma﻿̈rta – Rekommendationer 2002. (2002).

56 Swedish Medical Products Agency. *Läkemedel vid långvarig smärta hos barn och vuxna – behandlings­rekommendation*, <<https://web.archive.org/web/20210619091916/https://www.lakemedelsverket.se/sv/behandling-och-forskrivning/behandlingsrekommendationer/sok-behandlingsrekommendationer/lakemedel-vid-langvarig-smarta-hos-barn-och-vuxna--behandlingsrekommendation>> (2017).

57 Läkemedelsverket. *Förskrivning av opioider i Sverige. Läkemedel, doser och diagnoser*, <<https://web.archive.org/web/20210802095852/https://www.lakemedelsverket.se/49e69f/globalassets/dokument/publikationer/lakemedelsprodukter-och-narkotika/forskrivning-av-opioider-i-sverige-2020-1.pdf>> (2020).

58 Taiwan Food and Drug Administration & Department of Health Taiwan. Physician Guidelines on Clinical Use of Pethidine 2011. (2011).

59 Taiwan Food and Drug Administration. Guidelines on Clinical Use of Pethidine. (2017).

60 Taiwan Food and Drug Administration. *Guidelines on Clinical Use of Pethidine*, <<https://www.fda.gov.tw/tc/lawContent.aspx?cid=183&id=2937>> (2022).

61 Rolfs, R. T., Johnson, E., Williams, N. J. & Sundwall, D. N. Utah clinical guidelines on prescribing opioids for treatment of pain. *Journal of pain & palliative care pharmacotherapy* **24**, 219-235 (2010).

62 Berland, D. *et al.* Managing Chronic Non-Terminal Pain in Adults, Including Prescribing Controlled Substances. *Ann Arbor, MI: Univ of Michigan Health System* (2012).

63 US Department of Veterans Affairs & Defense, U. D. o., (2003).

64 Department of Veterans Affairs & Department of Defense. *Clinical Practice Guideline for Management of Opioid Therapy for Chronic Pain*, <<https://web.archive.org/web/20100527093311/http://www.healthquality.va.gov/cot/cot_310_sum.pdf>> (2010).

65 Rosenberg, J. M., Bilka, B. M., Wilson, S. M. & Spevak, C. Opioid therapy for chronic pain: overview of the 2017 US Department of Veterans Affairs and US Department of Defense Clinical Practice Guideline. *Pain Medicine* **19**, 928-941 (2018).

66 Sandbrink, F. *et al.* The use of opioids in the management of chronic pain: synopsis of the 2022 updated US Department of Veterans Affairs and US Department of Defense Clinical Practice Guideline. *Annals of internal medicine* **176**, 388-397 (2023).

67 Western Australia Drug and Alcohol Office. Quick clinical guideline for the use of opioids in chronic non-malignant pain. (2009).

68 Washington State Department of Labor and Industries. *Guideline for prescribing opioids to treat pain in injured workers*, <<https://web.archive.org/web/20140730083753/https://www.lni.wa.gov/ClaimsIns/Files/OMD/MedTreat/FINALOpioidGuideline010713.pdf>> (2013).

69 Work Loss Data Institute. Pain (chronic) (Encinitas, CA, 2011).

70 State Medical Society of Wisconsin. Statement on the use of opioids for the treatment of chronic pain. *Wisconsin Medical Journal* **100**, 22-25 (2001).

71 Wisconsin Medical Society Task Force on Pain Management. Guidelines for the assessment and management of chronic pain. *Wmj* **103**, 13-42 (2004).

72 Engrossed Substitute House Bill 2876, chapter 209, Laws of 2010; effective date June 10, 2010.

73 Goudman, L., Moens, M. & Pilitsis, J. G. Incidence and Prevalence of Pain Medication Prescriptions in Pathologies with a Potential for Chronic Pain. *Anesthesiology* **140**, 524-537 (2024). <https://doi.org/10.1097/ALN.0000000000004863>

74 Han, Y. *et al.* Assessment of Prescription Analgesic Use in Older Adults With and Without Chronic Kidney Disease and Outcomes. *JAMA Netw Open* **3**, e2016839 (2020). <https://doi.org/10.1001/jamanetworkopen.2020.16839>

75 Holmer, H. K., Gilbert, T. A., Ashraf, A. J., O'Neil, M. E. & Carlson, K. F. Opioid and Sedative-Hypnotic Prescriptions Among Post-9/11 Veteran VA Users Nationwide With Traumatic Brain Injury, 2012-2020. *J Head Trauma Rehabil* **36**, 354-363 (2021). <https://doi.org/10.1097/HTR.0000000000000712>

76 Kimmel, P. L. *et al.* Opioid Prescriptions for US Patients Undergoing Long-Term Dialysis or with Kidney Transplant from 2011 to 2020. *J Am Soc Nephrol* **36**, 108-121 (2025). <https://doi.org/10.1681/ASN.0000000000000478>

77 Lee, Y. C., Kremer, J., Guan, H., Greenberg, J. & Solomon, D. H. Chronic Opioid Use in Rheumatoid Arthritis: Prevalence and Predictors. *Arthritis Rheumatol* **71**, 670-677 (2019). <https://doi.org/10.1002/art.40789>

78 Richter, M. D., Achenbach, S. J., Zamora-Legoff, J. A., Crowson, C. S. & Matteson, E. L. Opioid use in patients with polymyalgia rheumatica. *Clin Exp Rheumatol* **35**, 1014-1017 (2017).

79 Rogal, S. S. *et al.* Characteristics of Opioid Prescriptions to Veterans With Cirrhosis. *Clin Gastroenterol Hepatol* **17**, 1165-1174 e1163 (2019). <https://doi.org/10.1016/j.cgh.2018.10.021>

80 Scott, I. C., Bailey, J., White, C. R., Mallen, C. D. & Muller, S. Analgesic prescribing in patients with inflammatory arthritis in England: an observational study using electronic healthcare record data. *Rheumatology (Oxford)* **61**, 3201-3211 (2022). <https://doi.org/10.1093/rheumatology/keab870>

81 Whitney, D. G., Peterson, M. D. & Hurvitz, E. A. Population- and individual-level trajectories of opioid prescription patterns among adults with cerebral palsy: a retrospective cohort study. *Int J Clin Pharm* **45**, 669-680 (2023). <https://doi.org/10.1007/s11096-023-01553-5>

82 Curtis, J. R. *et al.* Changing Trends in Opioid Use Among Patients With Rheumatoid Arthritis in the United States. *Arthritis Rheumatol* **69**, 1733-1740 (2017). <https://doi.org/10.1002/art.40152>

83 Zamora-Legoff, J. A. *et al.* Opioid use in patients with rheumatoid arthritis 2005-2014: a population-based comparative study. *Clin Rheumatol* **35**, 1137-1144 (2016). <https://doi.org/10.1007/s10067-016-3239-4>

84 Ahn, S. S., Han, M., Jung, I. & Kim, C. Y. Association of oral opioid usage and mortality in patients with inflammatory arthritides: a Korean nationwide cohort study. *Rheumatology (Oxford)* **64**, 4181-4189 (2025). <https://doi.org/10.1093/rheumatology/keaf135>

85 Crabtree, A., Rose, C., Chong, M. & Smolina, K. Effects of the new prescribing standards in British Columbia on consumption of opioids and benzodiazepines and z drugs. *Can Fam Physician* **65**, e231-e237 (2019).

86 Sears, J. M., Edmonds, A. T. & Fulton-Kehoe, D. Tracking Opioid Prescribing Metrics in Washington State (2012-2017): Differences by County-Level Urban-Rural and Economic Distress Classifications. *J Rural Health* **36**, 152-166 (2020). <https://doi.org/10.1111/jrh.12400>

87 Zhu, W., Chernew, M. E., Sherry, T. B. & Maestas, N. Initial Opioid Prescriptions among U.S. Commercially Insured Patients, 2012-2017. *N Engl J Med* **380**, 1043-1052 (2019). <https://doi.org/10.1056/NEJMsa1807069>

88 Bhattacharya, K. *et al.* Prescription Opioid Use in Older Adults: Trends and Changes in New and Long-Term Use in the United States, 2013-2020. *Drugs Aging* **42**, 887-898 (2025). <https://doi.org/10.1007/s40266-025-01237-x>

89 Franklin, G. M. *et al.* Bending the prescription opioid dosing and mortality curves: impact of the Washington State opioid dosing guideline. *Am J Ind Med* **55**, 325-331 (2012). <https://doi.org/10.1002/ajim.21998>

90 Garcia, M. M. *et al.* Impact of Sequential Opioid Dose Reduction Interventions in a State Medicaid Program Between 2002 and 2017. *J Pain* **20**, 876-884 (2019). <https://doi.org/10.1016/j.jpain.2019.01.008>

91 Guy, G. P., Jr. *et al.* Vital Signs: Changes in Opioid Prescribing in the United States, 2006-2015. *MMWR Morb Mortal Wkly Rep* **66**, 697-704 (2017). <https://doi.org/10.15585/mmwr.mm6626a4>

92 Salvatore, P. P., Guy, G. P. & Mikosz, C. A. Changes in Opioid Dispensing by Medical Specialties After the Release of the 2016 CDC Guideline for Prescribing Opioids for Chronic Pain. *Pain Med* **23**, 1908-1914 (2022). <https://doi.org/10.1093/pm/pnac068>

93 Rolova, G. *et al.* Trends in opioid prescribing in Scandinavian countries from 2010 to 2023: Insights from multi-metric evaluation. *Br J Clin Pharmacol* (2025). <https://doi.org/10.1002/bcp.70177>

94 Ali, M. M. *et al.* Potentially Problematic Opioid Prescriptions Among Individuals With Private Insurance and Medicaid. *Psychiatr Serv* **70**, 681-688 (2019). <https://doi.org/10.1176/appi.ps.201800555>

95 Hechter, R. C. *et al.* Chronic and Sustained High-Dose Opioid Use in an Integrated Health System. *Am J Prev Med* **64**, 167-174 (2023). <https://doi.org/10.1016/j.amepre.2022.09.013>

96 Adalbert, J. R., Varshney, K., Hom, J. & Ilyas, A. M. Methadone Prescribing for Pain Management in Pennsylvania per the Prescription Drug Monitoring Program, 2016-2020. *Cureus* **14**, e28583 (2022). <https://doi.org/10.7759/cureus.28583>

97 Adewumi, A. D. *et al.* Pharmaceutical opioids utilisation by dose, formulation, and socioeconomic status in Queensland, Australia: a population study over 22 years. *Int J Clin Pharm* **43**, 328-339 (2021). <https://doi.org/10.1007/s11096-020-01155-5>

98 Chai, G. *et al.* New Opioid Analgesic Approvals and Outpatient Utilization of Opioid Analgesics in the United States, 1997 through 2015. *Anesthesiology* **128**, 953-966 (2018). <https://doi.org/10.1097/aln.0000000000002187>

99 Champagne-Langabeer, T., Madu, R., Giri, S., Stotts, A. L. & Langabeer, J. R. Opioid prescribing patterns and overdose deaths in Texas. *Subst Abus* **42**, 161-167 (2021). <https://doi.org/10.1080/08897077.2019.1675114>

100 Dart, R. C. *et al.* Trends in opioid analgesic abuse and mortality in the United States. *N Engl J Med* **372**, 241-248 (2015). <https://doi.org/10.1056/NEJMsa1406143>

101 Gupta, A., Lindstrom, S. & Shevatekar, G. Reducing Opioid Overprescribing by Educating, Monitoring and Collaborating with Clinicians: A Quality Improvement Study. *Cureus* **12**, e7778 (2020). <https://doi.org/10.7759/cureus.7778>

102 Hébert, H. L., Morales, D. R., Torrance, N., Smith, B. H. & Colvin, L. A. Assessing the impact of a national clinical guideline for the management of chronic pain on opioid prescribing rates: a controlled interrupted time series analysis. *Implementation Science* **17**, 77 (2022). <https://doi.org/10.1186/s13012-022-01251-2>

103 Kazanis, W. *et al.* Opioid Use Patterns Among Active Duty Service Members and Civilians: 2006-2014. *Mil Med* **183**, e157-e164 (2018). <https://doi.org/10.1093/milmed/usx014>

104 Schieber, L. Z. *et al.* Trends and Patterns of Geographic Variation in Opioid Prescribing Practices by State, United States, 2006-2017. *JAMA Network Open* **2**, e190665-e190665 (2019). <https://doi.org/10.1001/jamanetworkopen.2019.0665>

105 Pritchard, K. T., Baillargeon, J., Lee, W. C., Raji, M. A. & Kuo, Y. F. Trends in the Use of Opioids vs Nonpharmacologic Treatments in Adults With Pain, 2011-2019. *JAMA Netw Open* **5**, e2240612 (2022). <https://doi.org/10.1001/jamanetworkopen.2022.40612>

106 Rancu, A. L. *et al.* Opioid Prescribing Trends Following Lumbar Discectomy. *J Am Acad Orthop Surg* **33**, 1054-1059 (2024). <https://doi.org/10.5435/JAAOS-D-24-00908>

107 Rancu, A. L. *et al.* Opioid Use Following Open Reduction and Internal Fixation of Mandibular Fractures. *J Craniofac Surg* **36**, 503-507 (2025). <https://doi.org/10.1097/SCS.0000000000010930>
